# The Origin of Stereoselectivity in the Hydrogenation of Oximes Catalyzed by Iridium Complexes: A DFT Mechanistic Study

**DOI:** 10.3390/molecules27238349

**Published:** 2022-11-30

**Authors:** Qaim Ali, Yongyong Chen, Ruixue Zhang, Zhewei Li, Yanhui Tang, Min Pu, Ming Lei

**Affiliations:** 1 State Key Laboratory of Chemical Resource Engineering, Institute of Computational Chemistry, College of Chemistry, Beijing University of Chemical Technology, Beijing 100029, China; 2 School of Materials Design and Engineering, Beijing Institute of Fashion Technology, Beijing 100029, China

**Keywords:** DFT, non-covalent interactions, stereoselectivity, asymmetric hydrogenation, oxime

## Abstract

Herein the reaction mechanism and the origin of stereoselectivity of asymmetric hydrogenation of oximes to hydroxylamines catalyzed by the cyclometalated iridium (III) complexes with chiral substituted single cyclopentadienyl ligands (Ir catalysts **A1** and **B1**) under acidic condition were unveiled using DFT calculations. The catalytic cycle for this reaction consists of the dihydrogen activation step and the hydride transfer step. The calculated results indicate that the hydride transfer step is the chirality-determining step and the involvement of methanesulfonate anion (MsO^−^) in this reaction is of importance in the asymmetric hydrogenation of oximes catalyzed by **A1** and **B1**. The calculated energy barriers for the hydride transfer steps without an MsO^−^ anion are higher than those with an MsO^−^ anion. The differences in Gibbs free energies between **TSA5−1fR**/**TSA5−1fS** and **TSB5−1fR**/**TSB5−1fS** are 13.8/13.2 (ΔΔ*G*^‡^ = 0.6 kcal/mol) and 7.5/5.6 (ΔΔ*G*^‡^ = 1.9 kcal/mol) kcal/mol for the hydride transfer step of substrate protonated oximes with *E* configuration (***E*−2a−H^+^**) with MsO^−^ anion to chiral hydroxylamines product ***R*−3a**/***S*−3a** catalyzed by **A1** and **B1**, respectively. According to the Curtin–Hammet principle, the major products are hydroxylamines ***S*−3a** for the reaction catalyzed by **A1** and **B1**, which agrees well with the experimental results. This is due to the non-covalent interactions among the protonated substrate, MsO^−^ anion and catalytic species. The hydrogen bond could not only stabilize the catalytic species, but also change the preference of stereoselectivity of this reaction.

## 1. Introduction

**Scheme 1:**
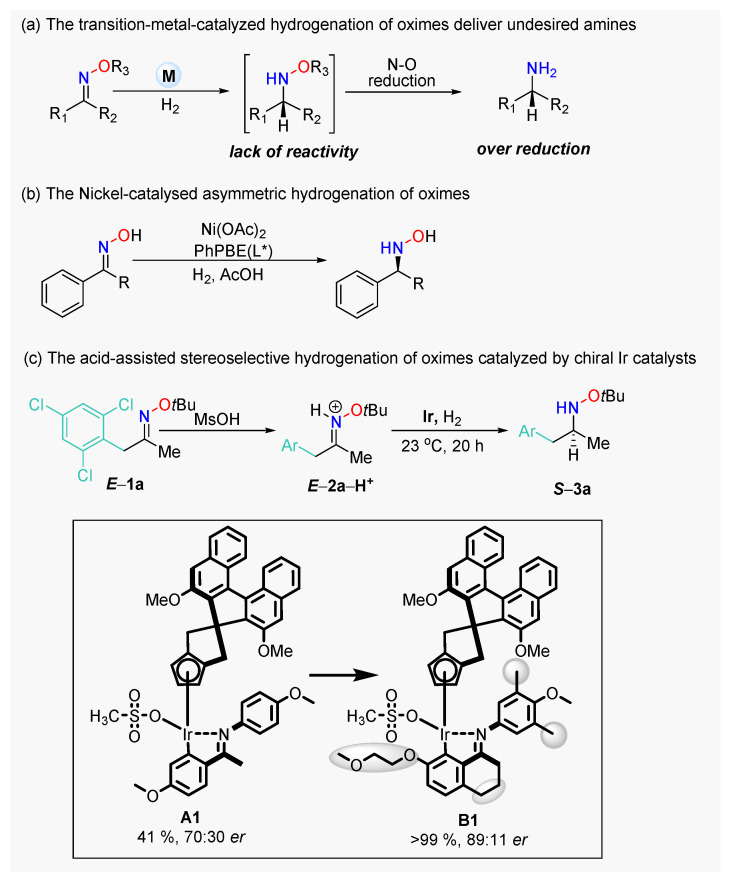
(**a**) The transition-metal-catalyzed hydrogenation of oximes deliver undesired amines [3], (**b**) the Nickel-catalyzed asymmetric hydrogenation of oximes [9] and (**c**) the acid-assisted stereoselective hydrogenation of oximes catalyzed by chiral Ir catalysts [10].

The chiral hydroxylamines are one of the important organic intermediates in pharmaceutical and agricultural industries, which could be attributed to the reactive N–O bond in the structural motif. The molecules owning the N-alkoxy amine group are very common in a wide range of bioactive and pharmaceutical products [1]. Among the syntheses of chiral amines, the asymmetric hydrogenation of the C=N double bond catalyzed by transition-metal complexes is one of the most efficient methods to prepare enantiomers at an industrial scale [2]. However, the selective reduction of oximes to the corresponding chiral hydroxylamine derivatives remains a challenge because the undesired reductive cleavage of the labile N–O bond leads to undesired primary amines (see Scheme 1a) [3]. Chiral hydroxylamine could be obtained by partial oxidation of chiral amines, but this method could either involve multiple steps or be prone to overreaction [4,5,6,7]. In 2014, Oestreich et al. reported the reductive hydrogenation of oxime ether catalyzed by B(C_6_F_5_)_3_ at room temperature [8]. Recently, Zhang et al. realized Ni-catalyzed asymmetric hydrogenation of oxides to chiral hydroxyl amines via weak attractive interactions between the catalyst and substrate (Scheme 1b) [9]. In 2020, Cramer et al. reported this reaction catalyzed by cyclometallated iridium (III) complexes with chiral substituted single cyclopentadienyl ligands and C,N-bidentate aryl imine ligand (see Scheme 1c) [10]. Despite the experimental reports above, the effective strategy of asymmetric hydrogenation of oxime to hydroxylamine is still relatively scarce. The reaction by Cramer et al. proceeded at room temperature with 98% *ee* and the turnover number (TON) reached 4000 before becoming inactive. This transformation was comprised of the quantitative protonation of the substrate and subsequent hydride transfer. The hydride could be transferred to the substrate either directly or with the help of the anion mediation by means of non-covalent contacts with the transition-metal catalytic species and the protonated substrates. Although it is obvious that the cyclometallated iridium complexes such as **A1** and **B1** owning chiral cyclopentadienyl ligand with binaphthyl as backbone and an achiral C,N-bidentate aryl imine ligand are of importance in the asymmetric hydrogenation of oximes, the reaction mechanism and the origin of stereoselectivity for this reaction are still unclear. Therefore, herein a DFT mechanistic study was performed in order to unveil the nature of this reaction catalyzed by catalysts **A1** and **B1**.

**Scheme 2:**
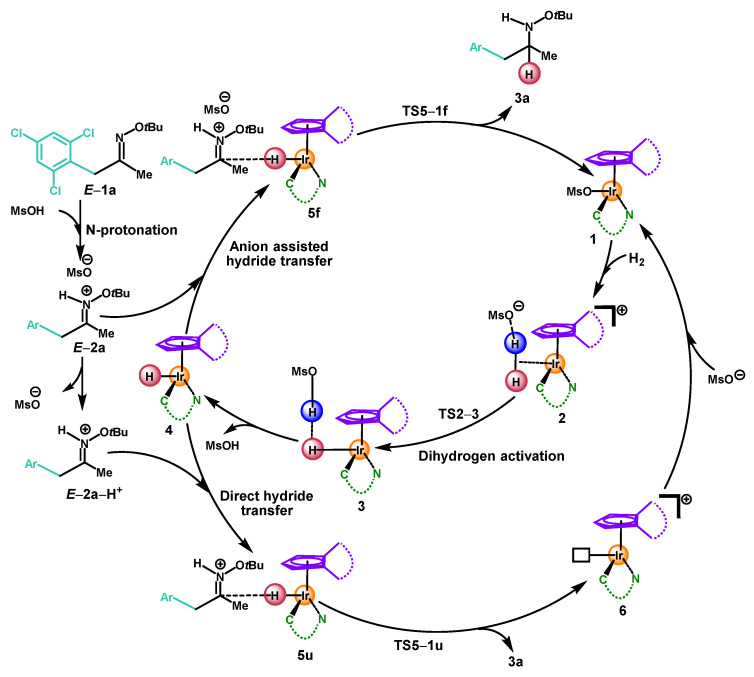
Proposed reaction mechanism for the asymmetric hydrogenation of oximes to hydroxylamines catalyzed by iridium complexes.

As shown in Scheme 2, the catalytic cycle of this reaction consists of the dihydrogen activation (DA) step and the hydride transfer (HT) step. In the dihydrogen activation step, one molecular hydrogen interacts with **1** by replacing the weakly coordinated methanesulfonate anion (MsO^−^) ligand to afford intermediate **2**, then the dihydrogen is heterolytically split with the assistance of the MsO^−^ anion via the transition state **TS2−3** to form intermediate **3.** Before the addition of oxime substates in the catalytic cycle, the original oxime substrate ***E*−1a** is protonated by MsOH to achieve protonated adduct ***E*−2a−H^+^** without MsO^−^ anion or ***E*−2a** with MsO^−^ anion. In the subsequent hydride transfer step, the methanesulfonic acid (MsOH) is released to produce intermediate **4**; the protonated substrates (***E*−2a−H^+^** or ***E*−2a**) could interact with **4** to achieve the chiral hydroxylamine product **3a** and regenerate **1** completing the catalytic cycle. In this work, the reaction mechanism of the asymmetric hydrogenation of oximes to hydroxylamines was discussed, the origin of the stereoselectivity was unveiled, and the important role of the MsO^−^ anion in this reaction was described. This might shed lights on the development of transition-metal catalysts based on the mechanism of the asymmetric hydrogenation of oximes in the future.

## 2. Results and Discussion

**Figure 1:**
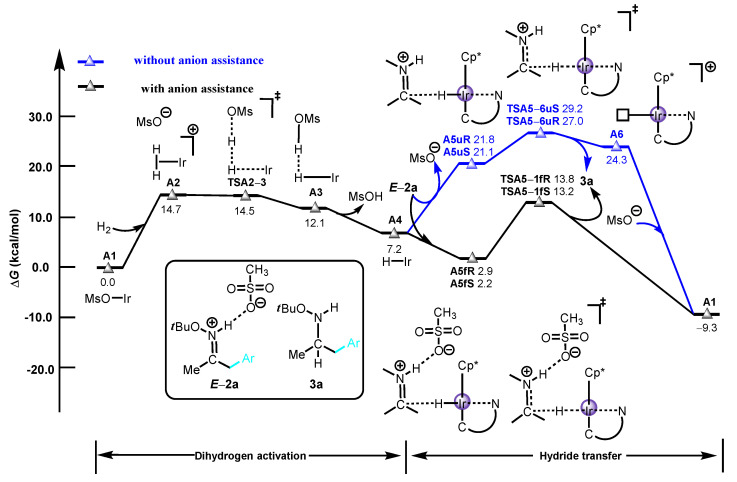
The Gibbs free energy profiles of asymmetric hydrogenation of oximes to hydroxylamines catalyzed by Ir complex **A1** (unit: kcal/mol). * represents chirality and ‡ means this is a transition state.

The catalytic cycle of the asymmetric hydrogenation of oxime to hydroxyamine catalyzed by Ir catalysts (**A1** and **B1**) includes the dihydrogen activation step and the hydride transfer step. In this work, the (*S*)-Ir catalysts bearing a chiral binaphthyl-derived cyclopentadienyl ligand was used as the catalytic species for this reaction, which was reported by experiments [10]. Figure 1 presents the Gibbs free energy profiles for the asymmetric hydrogenation of *E*-oxime (***E*−1a**) catalyzed by **A1**; the energies of the stationary points are relative to corresponding starting point **A1** (see that of hydrogenation of *Z*-oxime in Figure S1 of SM). *E*-oxime was used because it is more stable than *Z*-oxime. In the dihydrogen activation step, one molecule hydrogen coordinates with Ir center by replacing the weakly coordinated MsO^−^ anion to form intermediate **A2**, which is endergonic by 14.7 kcal/mol. Then the coordinated dihydrogen causes a heterolytic splitting with the assistance of the MsO^−^ anion. The free energy barrier from **A1** to **A3** is 14.5 kcal/mol via **TSA2−3**. **A3** forms iridium hydride intermediate **A4** with the release of one molecule of MsOH, which is exergonic by 4.9 kcal/mol. The following hydride transfer step could be the direct hydride transfer without the involvement of the MsO^−^ anion or the MsO^−^-assisted hydride transfer. In the direct hydride transfer step, the substrate tethering with one molecule MsOH, ***E*−2a**, could produce the protonated oxime ***E*−2a−H^+^** with the removal of one MsO^−^ anion. Then ***E*−2a−H^+^** approaches the Ir center of **A4** along the *si*-face direction to form intermediate **A5uR** leading to the hydroxylamine product ***R*−3a**. If it approaches the Ir center of **A4** along the *re*-face direction, the intermediate **A5uS** is generated leading to ***S*−3a** hydroxylamine. The “u” in the names of stationary points such as **A5uS** represents stationary points along an unfavorable pathway, the “f” is used for those along a favorable pathway, and “S” or “R” denotes the reaction pathways leading to S or R products, respectively. In the direct hydride transfer step from **A5uR**/**A5uS** to **A6**, the hydride is transferred from an Ir center to the carbon atom of pronated oxime moiety of **A5uR**/**A5uS** directly via **TSA5−6uR**/**TSA5−6uS**. The free energy barriers for this step are 5.2/8.1 kcal/mol from **A5uR**/**A5uS** to **A6uR**/**A6uS** and hydroxylamines, respectively.

Finally, **A6uR**/**A6uS** combine with the MsO^−^ anion to regenerate the active catalytic species **A1** and the catalytic cycle is completed. It should be noted that the energetic span [11] of generating ***R*−3a**/***S*−3a** products are 27.0/29.2 kcal/mol (**A1→TSA5−6uR**/**TSA5−6uS**), respectively. That means that ***R*−3a** is the dominant product instead of ***S*−3a**, and the energetic span is high, which is contrary with the conditions for the experiment to be carried out at room temperature.

Therefore, the direct hydride transfer mode cannot explain well the experimental observation. The non-covalent interactions have been proposed to be of importance in the asymmetric reactions, which might stabilize the transition states, accelerate chemical reaction and regulate the stereoselectivity [9,12,13]. Note that the Gibbs free energy of the oxime substrate tethering with one molecule MsOH (***E*−2a**) is 17.3 kcal/mol lower than that of protonated oxime ***E*−2a−H^+^**, which could be more stable to form ***E*−2a** for ***E*−2a−H^+^** combining with the conjugated base MsO^−^ anion. There are non-covalent interactions including hydrogen bonds formed by ***E*−2a−H^+^**, MsO^−^ and Ir catalytic species after ***E*−2a** interacts with **A4**. It is exergonic by 4.3 and 5.0 kcal/mol to generate **A5fR** and **A5fS** from the interaction of ***E*−2a** and **A4**. In the subsequent MsO^−^-assisted hydride transfer step from **A5fR**/**A5fS** to **A1**+***R*−3a**/**A1**+***S*−3a**, the hydride is transferred from the Ir center to the carbon atom of oxime moiety. The free energy barriers for this step are 10.9 and 11.0 kcal/mol for the formation of ***R*−3a** and ***S*−3a** via **TSA5−1fR**/**TSA5−1fS**, respectively. Based on the energetic span model and Curtin–Hammet principle [14], the energetic spans of the reaction pathways to achieve ***R*-3a** and ***S*-3a** products adopting the MsO^−^-assisted hydride transfer mode are 13.8 (**A1→TSA5−1fR**) and 13.2 (**A1→TSA5−1fS**) kcal/mol, respectively. It is obvious that the reaction pathways with the involvement of the MsO^−^ anion is much favorable than those without the MsO^−^ anion. The difference of the energetic spans for the reactions leading to R and S products are 0.6 kcal/mol (ΔΔ*G*^‡^ = 0.6 kcal/mol). The calculated results show that ***S*−3a** is the dominant product. The predicted enantioselectivity is 73:27 e.r., which agrees well with that obtained in the experiment (70:30 e.r.) [10]. This indicates that the non-covalent interactions including hydrogen bonds among ***E*−2a−H^+^**, MsO^−^ and Ir catalytic species are very important, as they could not only stabilize the catalyst but also turn over the stereoselectivity of the reaction. 

**Figure 2:**
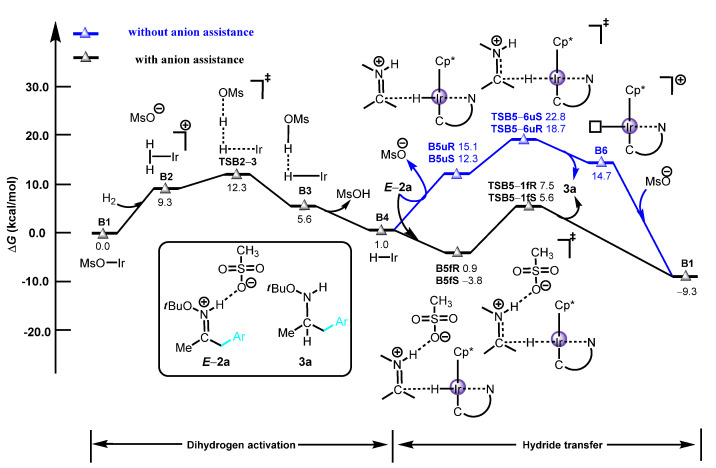
The Gibbs free energy profiles of asymmetric hydrogenation of oximes to hydroxylamines catalyzed by Ir complex **B1** (unit: kcal/mol). * represents chirality and ‡ means this is a transition state.

Meanwhile, the asymmetric hydrogenation of oxime ***E*−1a** to hydroxylamine catalyzed by Ir catalyst **B1** was also investigated using DFT method in this work. The free energy profiles for this reaction catalyzed by **B1** are shown in Figure 2. Following the similar reaction pathways as discussed for catalyst **A1** above, in the dihydrogen activation step from **B1** to **B4** molecular hydrogen replaces weakly coordinated the MsO^−^ anion of **B1** to afford **B2** at first, which is endergonic by 9.3 kcal/mol. Then **B2** proceeds with a heterolytic dihydrogen splitting to achieve **B3** via **TSB2−3** with a free energy barrier of 3.0 kcal/mol. Subsequently, **B3** releases MsOH to form iridium hydride intermediate **B4**. This step is exergonic by 4.6 kcal/mol. In the following hydride transfer step (the chirality-determining step), the hydride of metal center of **B4** could be stereoselectively transferred to the protonated substrate with or without MsO^−^ anion assistance. Similarly, MsOH tethered substrate ***E*−2a** releases the MsO^−^ anion to afford protonated substrate ***E*−2a−H^+^** before the interaction with the iridium hydride intermediate **B4**. In the situation without the participation of the MsO^−^ anion, ***E*−2a−H^+^** interacts with **B4** along its *Si*/*Re* face to form **B5uR**/**B5uS** intermediates, which could achieve the final chiral hydrogenated products. Instead, the intermediate **B5fR**/**B5fS** could be formed by the combination of **B4** and ***E*−2a** with the participation of the MsO^−^ anion. Similarly, the intermediates **B5fR**/**B5fS** are more stable than **B5uR**/**B5uS** due to the formation of hydrogen bonds among ***E*−2a−H^+^** and MsO^−^ anion in the former ones. The calculated free energy barriers of the MsO^—^assisted hydride transfer step of this reaction to achieve ***R*-3a** and ***S*-3a** products are 6.6 (**B5fR→TSB5f−1R**) and 9.4 (**B5fS→TSB5f−1S**) kcal/mol, respectively. Compared to the MsO^−^-assisted hydride transfer, those adopting the direct hydride transfer mode without the involvement of the MsO^−^ anion are 18.7 (**B1→TSB5−1uR**) and 22.8 (**B1→TSB5−1uS**) kcal/mol, respectively. It is obvious that the MsO^−^-assisted hydride transfer with MsO^−^ anion is much more favorable than the direct hydride transfer without MsO^−^ anion. In addition, according to the Curtin–Hammet principle, the difference in relative free energies between **TSB5−1fS** and **TSB5−1fR** is 1.9 kcal/mol (ΔΔ*G*^‡^ = 1.9 kcal/mol). This implies that the hydroxylamine ***S*−3a** is the favorable product and the predicted enantioselectivity is 96:4 e.r., this also agrees well with the result reported by experiment (89:11 e.r.) [10]. 

In general, the calculated results above for the asymmetric hydrogenation of oximes to hydroxylamines catalyzed by iridium complexes **A1** and **B1** could predict that the final favorable product would be ***S*−3a**, which is consistent with experimental results. The stereoselectivity of **B1** is better than that of **A1**. The hydride transfer step is the chirality-determining step and the MsO^−^ anion is proposed to be of importance in this asymmetric hydrogenation of oximes. The MsO^−^-assisted hydride transfer mode is superior to the direct hydride transfer mode.

**Figure 3:**
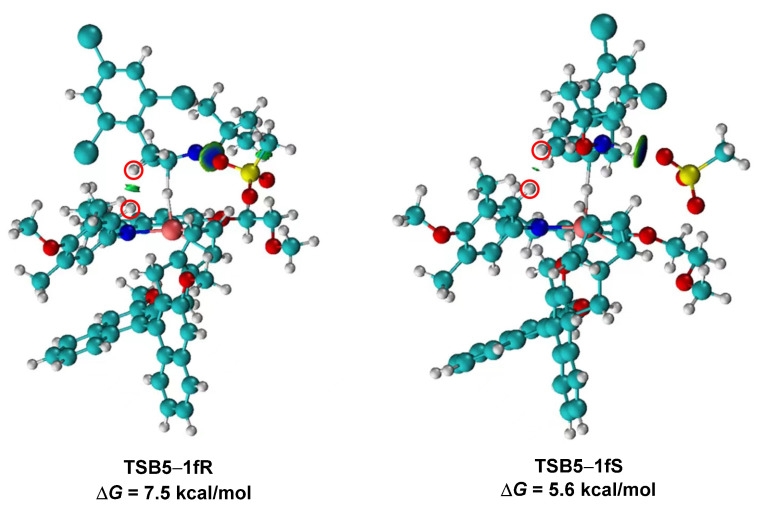
The IGMH analysis of the transition states of the MsO^−^-assisted hydride transfer step of the reaction by catalyst **B1** (*δ*g^inter^ = 0.008 a.u.).

In order to explore the origin of stereoselectivity of asymmetric hydrogenation of oximes to hydroxylamines catalyzed by chiral Ir catalysts (**A1** and **B1**), the independent gradient model based on Hirshfeld partition of molecular density (IGMH) analysis were employed using Multiwfn software and were visualized using VMD software [15]. IGMH analysis could characterize and visualize the non-covalent interactions of key structures along reaction pathways for this reaction. IGMH analysis of key transition state structures **TSB5−1fR**/**TSB5−1fS** for the hydride transfer step in the asymmetric hydrogenation of oximes to hydroxylamines catalyzed by **B1** are shown in Figure 3. It could be seen that there existed a strong hydrogen bond interaction between the MsO^−^ anion and the substrate in the anion-assisted hydride transfer step. This hydrogen bond interaction could stabilize the catalyst and make the MsO^−^ anion-assisted hydride transfer mode superior to the direct hydride transfer. In addition, we found that the distance between the hydrogen atom of benzene ring moiety of catalyst **B1** and the hydrogen atom of the substrate is close (marked by a red cycle in Figure 3). The distance between the two hydrogen atoms in **TSB5−1fR**/**TSB5−1fS** is 1.904 Å/2.191 Å, respectively. IGMH analysis visualized this van der Waals repulsion, and the distance in **TSB5−1fR** is closer than that in **TSB5−1fS**, implying that the repulsion interaction in **TSB5−1fR** is greater than that in **TSB5−1fS**. This might be the origins of stereoselectivity of this reaction and the instability of **TSB5−1fR** compared to **TSB5−1fS**. However, this recognition effect is weakened in the catalyst **A1** due to the smaller ligand and could not be visualized by IGMH analysis (see Figure S4 of SM). The distances between the two hydrogen atoms of **TSA5−1fR** and **TSA5−1fS** are 2.221 Å and 2.291 Å, respectively, which are longer than those of corresponding **TSB5−1fR** and **TSB5−1fS**.

## 3. Computational Methods

Being consistent with our previous research on the mechanisms of hydrogenation reaction catalyzed by transition-metal complexes [16,17,18,19,20,21,22,23,24], all the calculations were performed using the ωB97X-D/BSI method employing the Gaussian 09 program [25,26]. BSI indicates that the LANL2DZ basis set was used for Ir atom and 6-31G* basis set for all other non-metal atoms [27,28,29]. Additionally, single point energies were calculated with ORCA package at the ωB97M-V/def2-TZVP level using optimized geometries at the ωB97X-D/BSI level [30,31,32,33]. The solution model based on the density (SMD) solvation model using *tert*-amyl alcohol as the solvent (ε = 5.78) was employed in calculations [34]. The frequency analyses were performed to verify that all transition states have one and only one imaginary frequency. The intrinsic reaction coordinate (IRC) calculations were performed for key steps to confirm transition states connecting two desired minima [35]. The quasi-rigid-rotor harmonic oscillator was used to consider low-frequency contributions utilizing the Shermo code [36,37]. To correct the overestimations of entropy contributions due to using the ideal gas phase model in the Gaussian program, we applied a correction of (*n*-*m*)*1.9 for a process from *m* components to *n* components according to the reference [38]. The non-covalent interactions were observed by mapping the independent gradient model based on Hirshfeld partition (IGMH) surfaces using Multiwfn [39,40]. Unless otherwise stated, all energies of stationary points are the Gibbs free energies calculated at 298.15 K, 1.0 atm and compared to **A1** or **B1**. Total energies, Cartesian coordinates of all optimized structures and the calculation formula for enantioselectivity ratios (e.r.) are given in the Supplementary Materials (SM).

## 4. Conclusions

In summary, the reaction mechanism and the origin of stereoselectivity of asymmetric hydrogenation of oximes to hydroxylamines catalyzed by the cyclometalated iridium (III) complexes (**A1** and **B1**) with chiral substituted single cyclopentadienyl ligand were investigated using DFT method. The hydride transfer step is proposed to be the chirality-determining step. The reaction could proceed via the direct hydride transfer or the MsO^−^-assisted hydride transfer. The calculated results showed that the major product should be the hydroxylamines ***S*−3a** along the MsO^−^-assisted hydride transfer pathway, and that the involvement of the MsO^−^ anion be of importance in this asymmetric hydrogenation of oximes catalyzed by **A1** and **B1**. This agrees well with experimental results and implies that the non-covalent interactions including hydrogen bonds formed by the protonated substrate, MsO^−^ anion and Ir catalytic species could not only stabilize the catalyst but also turn over the stereoselectivity of the reaction. This work might provide theoretical insights for the mechanism-based development of transition-metal catalysts for the asymmetric hydrogenation of oximes in the future.

## Data Availability

All data is available in the main text or the Supplementary Materials.

## Supplementary Materials

The following supporting information can be downloaded at: https://www.mdpi.com/article/10.3390/molecules27238349/s1. Figure S1: The Gibbs free energy profiles of asymmetric hydrogenation of Z-oximes to hydroxylamines catalyzed by Ir complex **A1** (unit: kcal/mol). * represents chirality and ‡ means this is a transition state. Figure S2: The Gibbs free energy profiles of asymmetric hydrogenation of Z-oximes to hydroxylamines catalyzed by Ir complex **B1** (unit: kcal/mol). * represents chirality and ‡ means this is a transition state. Figure S3: Several possible forms of ***E*−2a**. Figure S4: The IGMH analysis of the transition states of the MsO−-assisted hydride transfer step of the reaction by catalyst **A1** and **B1** (δg^inter^ = 0.008 a.u.). Figure S5: The Gibbs free energy profiles for the hydride transfer step of the asymmetric hydrogenation of oximes to hydroxylamines catalyzed by Ir complex **A1** using different functional (unit: kcal/mol). * represents chirality and ‡ means this is a transition state. Table S1: The calculated absolute electronic energies (*E*, in a.u.), thermal free energies (*G*, in a.u.), and relative Gibbs energies (Δ*G*, in kcal/mol) (Calculated at 298.15 K and 1 atm). Table S2: Calculated imaginary frequencies of transition states at ωB97X-D/BSI level. Table S3: Atomic cartesian coordinates of intermediates and transition states (presented in Å). References [41,42,43] are cited in the Supplementary Materials.
