# Supplementary material for: The Origin of Stereoselectivity in the Hydrogenation of Oximes Catalyzed by Iridium Complexes: A DFT Mechanistic Study
Source: Molecules. 2022 Nov 30;27(23):8349. doi: 10.3390/molecules27238349 (PMC9737400; doi:10.3390/molecules27238349)
Supplement: Supplementary file 1 [file molecules-27-08349-s001.zip › molecules-1965683-supplementary.pdf]

Supporting Materials

The Origin of Stereoselectivity in the Hydrogenation of Oximes Catalyzed by Iridium Complexes: A DFT Mechanistic Study

Qaim Ali [1], Yongyong Chen [1], Ruixue Zhang [1], Zhewei Li [1], Yanhui Tang [1,2], Min Pu [1] and Ming Lei [1,*]

[1] State Key Laboratory of Chemical Resource Engineering, Institute of Computational Chemistry, College of Chemistry, Beijing University of Chemical Technology, Beijing 100029, China
[2] School of Materials Design and Engineering, Beijing Institute of Fashion Technology, Beijing 100029, China
* Correspondence: leim@mail.buct.edu.cn; Tel.: +86-10-6444-6598

1. Details of DFT calculations 2

2. Asymmetric Hydrogenation of *Z*-oximes to Hydroxylamines Catalyzed by Ir Complex A1 3

3. Asymmetric Hydrogenation of *Z*-oximes to Hydroxylamines Catalyzed by Ir Complex B1 4

4. Several Possible Forms of *E*-2a. 5

5. Independent Gradient Model Based on Hirshfeld Partition of Molecular Density (IGMH) analysis 6

6. The Hydride Transfer Step Catalyzed by Ir Complex A1 Using Different Functional 7

7. Computational Formula for *R*:*S* Ratios 8

8. Energies of All the Structures 9

9. Imaginary Frequencies of Transition States 12

10. Cartesian Coordinates of All the Structures 13

References 150

1. Details of DFT calculations

Details of DFT calculations: All the calculations were performed using ωB97X-D/BSI method employing the Gaussian 09 program [1-2]. BSI indicates that the LANL2DZ basis set was used for Ir atom and 6-31G* basis set for all other non-metal atoms [3-5]. Additionally, single point energies were calculated with ORCA package at the ωB97M-V/def2-TZVP level using optimized geometries at the ωB97X-D/BSI level [6-9]. The solution model based on the density (SMD) solvation model using *tert*-amyl alcohol as the solvent ($\varepsilon = 5.78$) was employed in calculations [10]. The frequency analyses were performed to verify that all transition states have one and only one imaginary frequency. The intrinsic reaction coordinate (IRC) calculations were performed for key steps to confirm transition states connecting two desired minima [11]. The quasi-rigid-rotor harmonic oscillator was used to consider low-frequency contributions utilizing the Shermo code [12-13]. To correct the overestimations of entropy contributions due to using the ideal gas phase model in Gaussian program, we applied a correction of $(n-m)*1.9$ for a process from m components to n components according to the reference [14]. Unless otherwise stated, all energies of stationary points are the Gibbs free energies calculated at 298.15 K, 1.0 atm and compared to **A1** or **B1**. The noncovalent interactions are observed by mapping the independent gradient model based on Hirshfeld partition (IGMH) surfaces using Multiwfn [15-16]. In order to verify the reliability of the DFT calculations used in this work, single point energies were calculated at the ωB97M-V/def2-TZVP, B3LYP-D3(BJ) [17-18]/def2-TZVP, PBE0-

D3(BJ) [19]/def2-TZVP level using optimized geometries at the ωB97X-D/BSI level to investigate the reaction mechanism of stereoselectivity of asymmetric hydrogenation of oximes to hydroxylamine.

2. Asymmetric Hydrogenation of *Z*-oximes to Hydroxylamines Catalyzed by Ir Complex A1

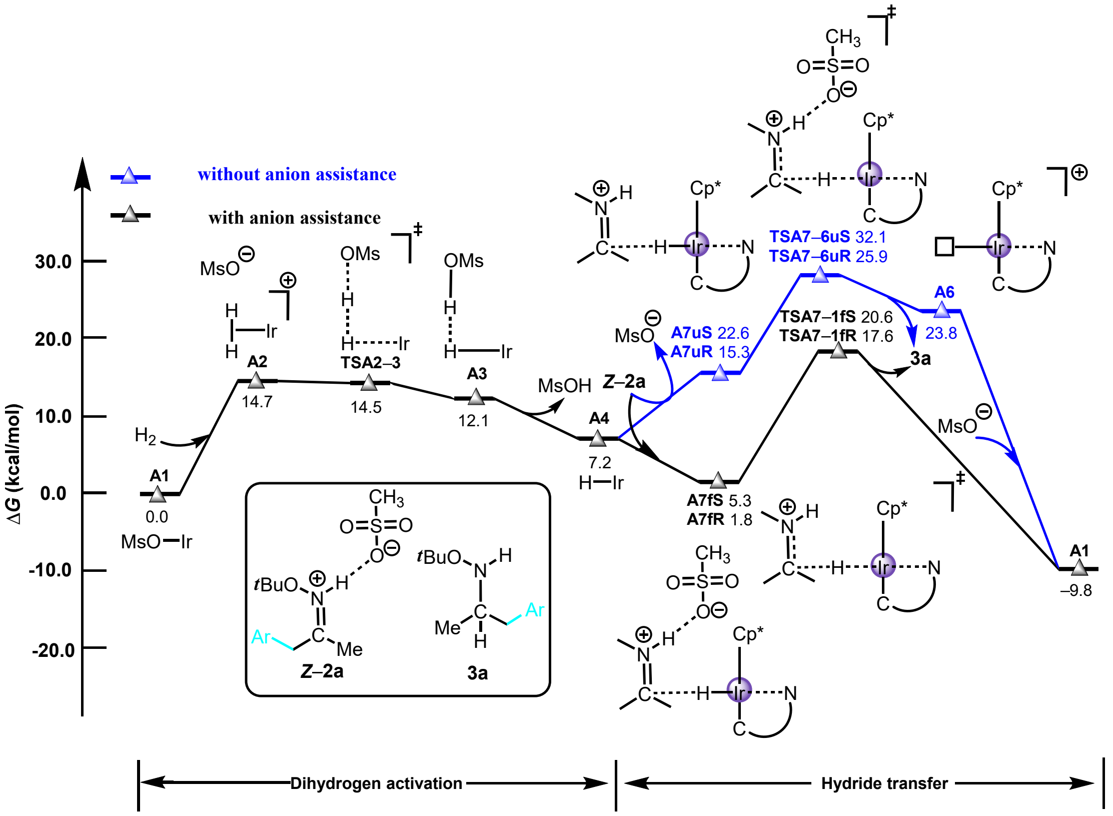

Figure S1. The Gibbs free energy profiles of asymmetric hydrogenation of *Z*-oximes to hydroxylamines catalyzed by Ir complex **A1** (unit: kcal/mol). * represents chirality and ‡ means this is a transition state.

3. Asymmetric Hydrogenation of *Z*-oximes to Hydroxylamines Catalyzed by Ir Complex B1

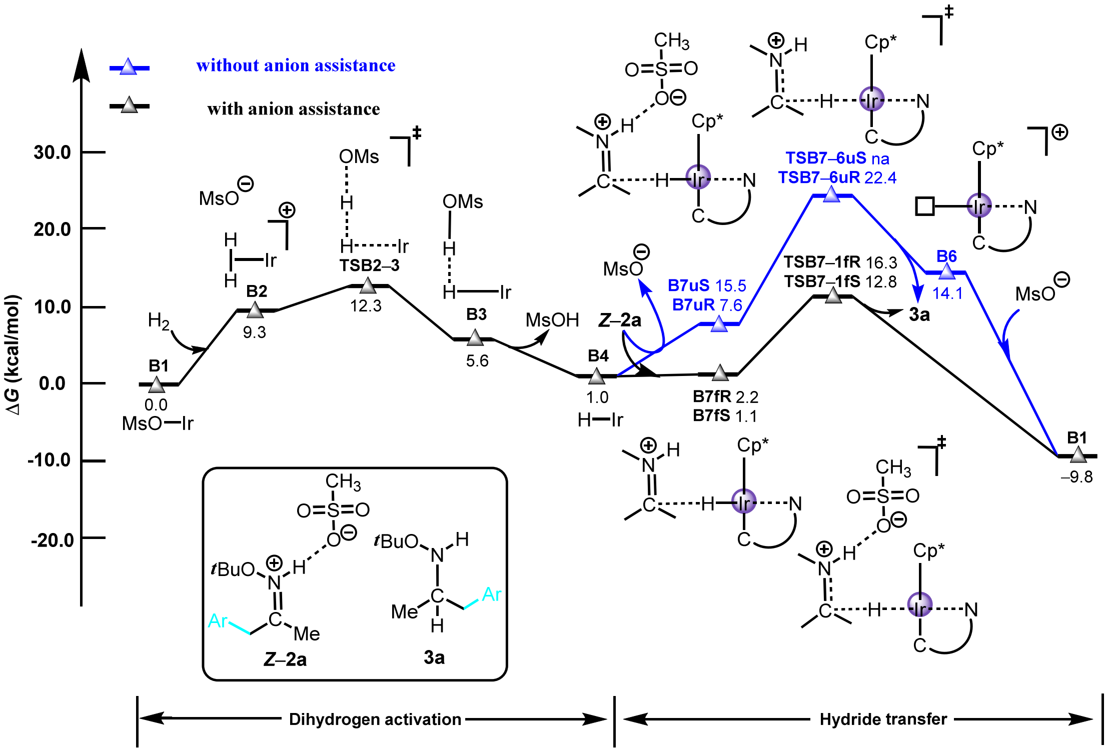

Figure S2. The Gibbs free energy profiles of asymmetric hydrogenation of *Z*-oximes to hydroxylamines catalyzed by Ir complex **B1** (unit: kcal/mol). * represents chirality and ‡ means this is a transition state.

4. Several Possible Forms of *E*−2a.

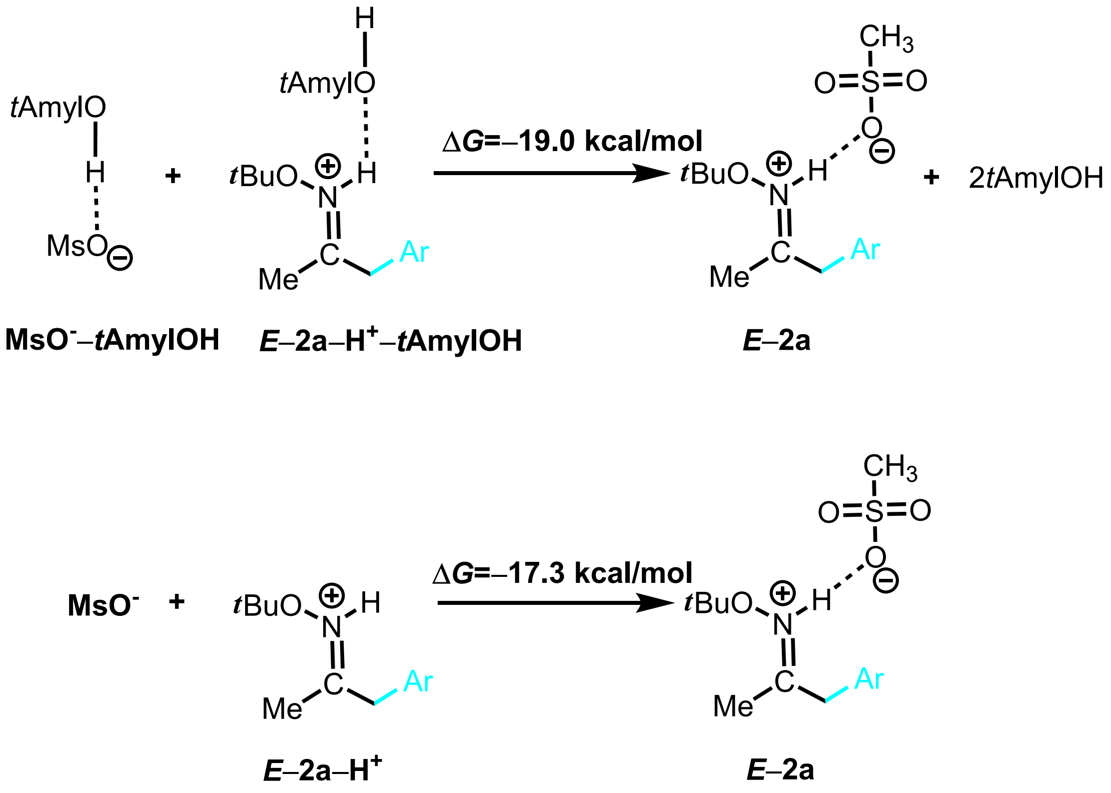

Figure S3. Several possible forms of ***E*−2a**.

5. Independent Gradient Model Based on Hirshfeld Partition of Molecular Density (IGMH) analysis

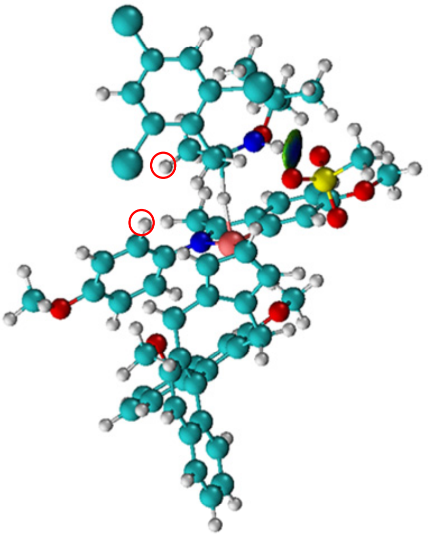

TSA5–1fR
Δ*G* = 13.8 kcal/mol

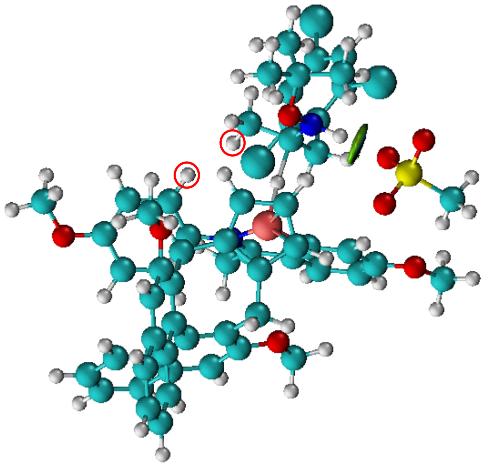

TSA5–1fS
Δ*G* = 13.2 kcal/mol

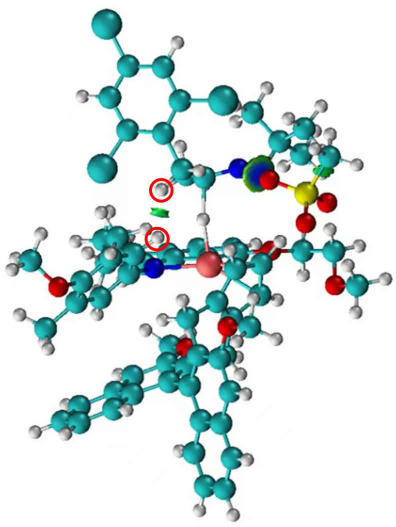

TSB5–1fR
Δ*G* = 7.5 kcal/mol

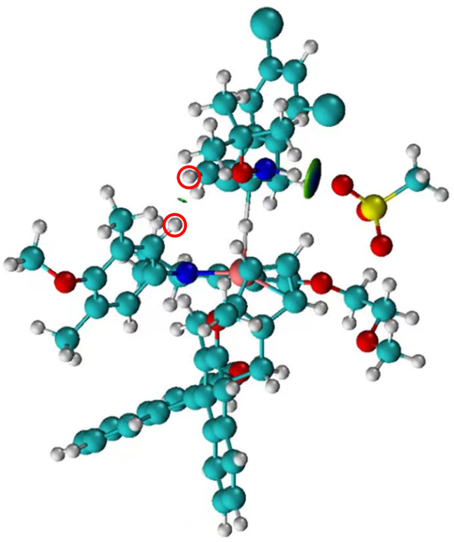

TSB5–1fS
Δ*G* = 5.6 kcal/mol

Figure S4. The IGMH analysis of the transition states of the MsO^{-}-assisted hydride transfer step of the reaction by catalyst **A1** and **B1** ($\delta g^{inter} = 0.008$ a.u.).

6. The Hydride Transfer Step Catalyzed by Ir Complex A1 Using Different Functional

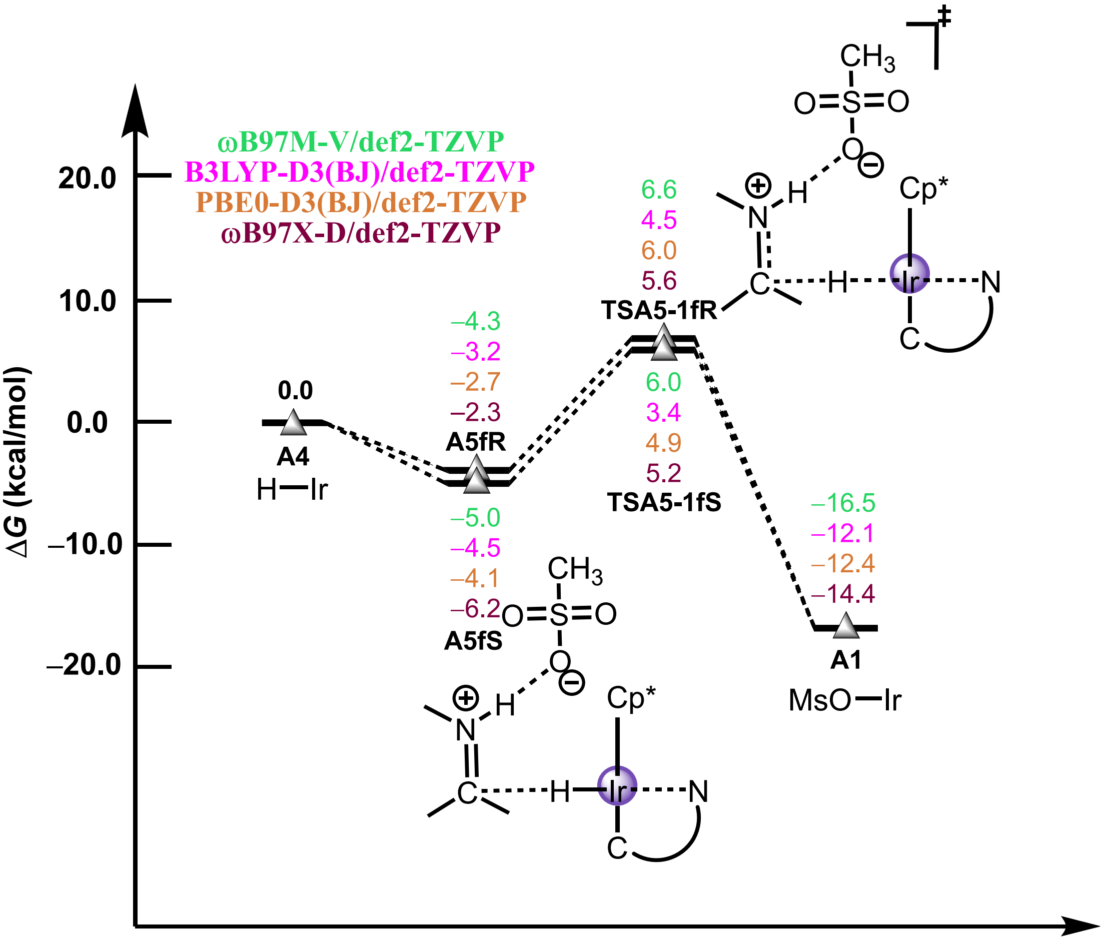

Figure S5. The Gibbs free energy profiles for the hydride transfer step of the asymmetric hydrogenation of oximes to hydroxylamines catalyzed by Ir complex **A1** using different functional (unit: kcal/mol). * represents chirality and ‡ means this is a transition state.

7. Computational Formula for *R*:*S* Ratios

$$R{:}S = [1/(1 + e^{-\Delta\Delta G/\mathrm{RT}})]{:}[e^{-\Delta\Delta G/\mathrm{RT}}/(1 + e^{-\Delta\Delta G/\mathrm{RT}})] \qquad (1)$$

* $\Delta\Delta G$ is the differences in the relative Gibbs free energy between two optimized geometries

8. Energies of All the Structures

Table S1. The calculated absolute electronic energies (*E*, in a.u.), thermal free energies (*G*, in a.u.), and relative Gibbs energies (Δ*G*, in kcal/mol) (Calculated at 298.15 K and 1 atm).

Complex	*E*	*G*	Δ*G*
**A1**	-2862.1431381	-2861.4216411	0.0
**H_2**	-1.1611025	-1.1624505	/
**A2**	-2863.2949914	-2862.5575964	14.7
**TSA2-3**	-2863.2920204	-2862.5580224	14.5
**A3**	-2863.2984835	-2862.5618085	12.1
**MsOH**	-664.4107948	-664.3773468	/
**A4**	-2198.8746592	-2198.1952972	7.2
***E*-2a**	-2679.9476123	-2679.6643653	/
**A5fR**	-4878.8578214	-4877.8634114	2.9
**A5fS**	-4878.8575130	-4877.8646250	2.2
**TSA5-1fR**	-4878.8429560	-4877.8461440	13.8
**TSA5-1fS**	-4878.8414797	-4877.8469997	13.2
***R*-3a**	-2016.7134580	-2016.4642710	/
***S*-3a**	-2016.7134580	-2016.4642950	/
**MsO^-**	-663.9640556	-663.9397076	/
**A5uR**	-4214.8417824	-4213.8966074	21.8
**A5uS**	-4214.8434338	-4213.8978378	21.1
**TSA5-6uR**	-4214.8346647	-4213.8884187	27.0
**TSA5-6uS**	-4214.8304886	-4213.8848666	29.2
**A6**	-2198.1030302	-2197.4313812	24.3
***Z*-2a**	-2679.9473710	-2679.6634950	/
**A7fR**	-4878.8563474	-4877.8643614	1.8

**A7fS**	-4878.8537967	-4877.8588337	5.3
**TSA7-1fR**	-4878.8331378	-4877.8391618	17.6
**TSA7-1fS**	-4878.8301471	-4877.8343451	20.6
**A7uR**	-4214.8533303	-4213.9062213	15.3
**A7uS**	-4214.8428416	-4213.8945416	22.6
**TSA7-6uR**	-4214.8384828	-4213.8892648	25.9
**TSA7-6uS**	-4214.8290717	-4213.8793067	32.1
**B1**	-3172.0115888	-3171.1400098	0
**B2**	-3173.1711861	-3172.2846821	9.3
**TSB2-3**	-3173.1638759	-3172.2798559	12.3
**B3**	-3173.1777169	-3172.2905779	5.6
**B4**	-2508.7540989	-2507.9235579	1.0
**B5fR**	-5188.7310825	-5187.5850415	0.9
**B5fS**	-5188.7358299	-5187.5925869	-3.9
**TSB5-1fR**	-5188.7212877	-5187.5745617	7.5
**TSB5-1fS**	-5188.7222309	-5187.5775129	5.6
**B5uR**	-4524.7216575	-4523.6257715	15.1
**B5uS**	-4524.7287542	-4523.6302152	12.3
**TSB5-6uR**	-4524.7166776	-4523.6200496	18.7
**TSB5-6uS**	-4524.7103344	-4523.6134174	22.8
**B6**	-2507.9858380	-2507.1651160	14.7
**B7fR**	-5188.7278272	-5187.5820932	2.2
**B7fS**	-5188.7303594	-5187.5837864	1.1
**TSB7-1fR**	-5188.7044267	-5187.5595377	16.3
**TSB7-1fS**	-5188.7102484	-5187.5651084	12.8
**B7uR**	-4524.7334575	-4523.6368625	7.6
**B7uS**	-4524.7218843	-4523.6242333	15.5
**TSB7-6uR**	-4524.7110613	-4523.6131103	22.5
**E-2a-H$^+$**	-2015.9388469	-2015.7000549	/

**t-AmylOH**	-272.9535732	-272.8179352	/
**E-2a-H⁺-tAmylOH**	-2288.9145460	-2288.5179930	/
**MsO⁻-tAmylOH**	-936.9299102	-936.7489352	/

9. Imaginary Frequencies of Transition States

Table S2. Calculated imaginary frequencies of transition states at ωB97X-D/BSI level.

**TSA2-3**	830.40*i*
**TSA5-1fR**	501.56*i*
**TSA5-1fS**	557.77*i*
**TSA5-6uR**	405.72*i*
**TSA5-6uS**	363.83*i*
**TSA7-1fR**	652.13*i*
**TSA7-1fS**	640.21*i*
**TSA7-8uR**	554.03*i*
**TSA7-8uS**	479.95*i*
**TSB2-3**	995.09*i*
**TSB5-1fR**	562.14*i*
**TSB5-1fS**	524.78*i*
**TSB5-6uR**	339.83*i*
**TSB5-6uS**	364.59*i*
**TSB7-1fR**	709.91*i*
**TSB7-1fS**	631.68*i*
**TSB7-8uR**	771.36*i*

10. Cartesian Coordinates of All the Structures

Table S3. Atomic cartesian coordinates of intermediates and transition states (presented in Å).

———————————————————————————————

A1

	Coordinates (Angstroms)		
	X	Y	Z
Ir	1.579866	-0.664223	-0.614455
C	2.885810	0.842289	-0.325679
N	1.332230	-0.260700	1.417340
C	3.691947	1.451099	-1.279749
C	4.617000	2.441354	-0.919030
C	4.765651	2.833074	0.417731
C	3.966720	2.230647	1.381210
C	3.033497	1.256608	1.023191
C	2.199782	0.877822	3.431308
C	2.148471	0.592741	1.964025
C	0.335122	-0.965475	2.149122
C	0.291869	-2.356312	2.101040
C	-0.743903	-3.053560	2.716294
C	-1.755543	-2.352798	3.379297
C	-1.705321	-0.956487	3.441598
C	-0.666599	-0.272100	2.830408
O	-2.822630	-2.935538	3.988549
C	-2.951915	-4.345533	3.909646
H	5.483555	3.590204	0.710347
H	1.687217	0.112444	4.016001
H	3.240878	0.931375	3.762555
H	1.067461	-2.900583	1.571245
H	-0.643266	0.811194	2.854264
H	-3.877898	-4.589605	4.433241
H	-3.025831	-4.682982	2.868645
H	-2.114409	-4.853030	4.403291
C	1.031559	-0.214715	-2.657975
C	1.310593	-1.601468	-2.594002
C	0.279281	-2.228261	-1.786530
C	-0.578559	-1.233793	-1.307622

C	-0.085593	0.062979	-1.782726
C	-0.808625	1.385786	-1.751091
C	-1.791118	-1.442263	-0.445796
C	-1.395818	1.770929	-0.412736
C	-3.016600	-0.877383	-1.124273
C	-3.726001	-1.689967	-2.061043
C	-4.836890	-1.214261	-2.709065
C	-5.278176	0.116670	-2.478841
C	-4.560339	0.956723	-1.584746
C	-3.416597	0.420231	-0.904704
C	-0.670931	2.677745	0.424715
C	-1.178672	3.086551	1.632209
C	-2.416593	2.568521	2.097233
C	-3.148464	1.658953	1.288947
C	-2.622106	1.301328	0.004668
C	-2.921368	2.913312	3.378237
C	-4.097254	2.374152	3.839026
C	-4.824198	1.464869	3.037192
C	-4.360170	1.116879	1.791866
H	1.575229	0.524796	-3.231342
H	2.115587	-2.113925	-3.103276
H	0.212427	-3.283403	-1.553061
H	-0.106943	2.156476	-2.076625
H	-1.608194	1.345687	-2.500428
H	-1.915172	-2.509074	-0.249794
H	-1.652571	-0.945411	0.516145
H	-0.643760	3.789350	2.261832
H	-2.353183	3.605638	3.994931
H	-4.470647	2.642019	4.823689
H	-5.748885	1.035268	3.411989
H	-4.917968	0.409302	1.185970
O	-3.197838	-2.928684	-2.239552
C	-3.793794	-3.775880	-3.207185
H	-3.754530	-3.322985	-4.204977
H	-3.205513	-4.694959	-3.203334
H	-4.833376	-4.010320	-2.948800
O	0.508584	3.107045	-0.086394
C	1.209242	4.125861	0.603759
H	1.511970	3.801690	1.605542
H	2.100971	4.329087	0.009325
H	0.602536	5.036718	0.679417
S	4.474367	-2.271305	-0.302135
C	5.547095	-1.209925	0.639355
H	5.341520	-0.169924	0.380894

H	5.367340	-1.379740	1.703307
H	6.581895	-1.457866	0.389151
O	4.849502	-3.657395	0.017583
O	4.609553	-1.891676	-1.716941
O	3.077441	-2.002626	0.244730
H	-0.751862	-4.136182	2.660112
H	1.733009	1.846673	3.644340
H	4.081263	2.536361	2.417656
H	3.648115	1.164888	-2.326441
O	5.336883	2.960031	-1.943793
C	6.302276	3.960850	-1.663010
H	6.754048	4.214131	-2.623448
H	5.835952	4.855686	-1.234153
H	7.078892	3.587267	-0.985233
H	-2.498161	-0.418490	3.952853
H	-5.388925	-1.826934	-3.413674
C	-6.420573	0.634609	-3.144990
C	-6.825034	1.931037	-2.944734
C	-4.993457	2.298189	-1.411754
C	-6.098796	2.773960	-2.073388
H	-7.701458	2.314778	-3.459797
H	-6.970680	-0.015566	-3.820942
H	-6.416066	3.802792	-1.928366
H	-4.439123	2.955335	-0.749178

A2

	Coordinates (Angstroms) X	Y	Z
Ir	-1.884346	0.254627	-0.632852
C	-3.100893	-1.343053	-0.292651
N	-1.591800	-0.100936	1.410914
C	-3.867534	-2.022104	-1.228444
C	-4.672465	-3.105902	-0.848006
C	-4.739672	-3.514215	0.489963
C	-3.985617	-2.831449	1.434805
C	-3.168080	-1.763757	1.057827
C	-2.288859	-1.333147	3.436153
C	-2.312439	-1.030160	1.972035
C	-0.610151	0.657509	2.114386
C	-0.642740	2.046998	2.062891
C	0.378072	2.799293	2.635474

C	1.445716	2.154544	3.265459
C	1.472077	0.757267	3.331894
C	0.451258	0.016716	2.757509
O	2.501187	2.793599	3.835733
C	2.562776	4.206737	3.728282
H	-5.364268	-4.344280	0.798237
H	-1.817039	-0.537221	4.013729
H	-1.458519	2.555814	1.559527
H	0.493801	-1.066371	2.775977
H	3.490825	4.503273	4.220017
H	2.589496	4.525525	2.679198
H	1.716957	4.684761	4.236835
C	-1.297133	-0.320364	-2.661290
C	-1.622766	1.056098	-2.692745
C	-0.634368	1.762470	-1.906506
C	0.242682	0.829250	-1.343160
C	-0.184441	-0.506544	-1.759284
C	0.614768	-1.781555	-1.684418
C	1.426147	1.159808	-0.483536
C	1.300008	-2.057545	-0.366596
C	2.684876	0.713493	-1.183782
C	3.311388	1.594340	-2.107146
C	4.424582	1.220044	-2.806128
C	4.966965	-0.079235	-2.636360
C	4.344647	-0.986444	-1.731958
C	3.187153	-0.559013	-1.003827
C	0.683484	-2.965512	0.551657
C	1.288428	-3.292997	1.739096
C	2.515179	-2.678405	2.106075
C	3.133268	-1.755472	1.220952
C	2.513594	-1.489474	-0.044637
C	3.116473	-2.934169	3.366703
C	4.273715	-2.294122	3.736480
C	4.883365	-1.366894	2.861023
C	4.326507	-1.106396	1.633076
H	-1.810198	-1.114980	-3.186889
H	-2.430700	1.512850	-3.248568
H	-0.614440	2.828608	-1.724379
H	-0.046301	-2.613155	-1.937116
H	1.373077	-1.724724	-2.475111
H	1.431034	2.234628	-0.297504
H	1.344295	0.659307	0.481397
H	0.834457	-3.994362	2.430916
H	2.638134	-3.640601	4.040884

H	4.721276	-2.493807	4.706117
H	5.792514	-0.855742	3.164202
H	4.794324	-0.383791	0.971990
H	-1.743442	-2.266166	3.619836
H	4.882909	1.914963	-3.504690
C	6.115606	-0.496039	-3.360251
C	6.626496	-1.758439	-3.198785
C	4.893231	-2.291181	-1.596240
C	6.005261	-2.665495	-2.308544
H	7.506877	-2.066854	-3.755590
H	6.581868	0.205947	-4.047195
H	6.412309	-3.665795	-2.190181
H	4.422554	-2.999011	-0.921090
H	-3.466579	0.992506	-0.218664
S	-2.667329	4.500812	-0.285512
C	-3.366789	6.133018	-0.118082
H	-2.893912	6.799416	-0.843790
H	-4.441907	6.080945	-0.307008
H	-3.184820	6.496814	0.896051
O	-3.358288	3.663860	0.731965
H	-2.924601	1.583842	0.052096
O	-1.215188	4.643670	-0.016266
O	-2.961761	4.067109	-1.672175
H	2.312036	0.264745	3.813413
H	0.329182	3.879786	2.561231
H	-4.037050	-3.150480	2.471992
H	-3.310891	-1.470842	3.801208
H	-3.877909	-1.736283	-2.276172
O	-5.357461	-3.695183	-1.856909
C	-6.196762	-4.800330	-1.557537
H	-6.997238	-4.515741	-0.865110
H	-6.634693	-5.105817	-2.509157
H	-5.622726	-5.635091	-1.139128
O	2.744720	2.833266	-2.311391
C	3.332325	3.873981	-1.536048
H	4.385557	4.019897	-1.806374
H	2.768653	4.782880	-1.756417
H	3.265734	3.657391	-0.461817
O	-0.505347	-3.469938	0.138165
C	-1.084852	-4.522391	0.888739
H	-0.394479	-5.370884	0.968022
H	-1.374839	-4.189417	1.891853
H	-1.976585	-4.824852	0.338545

A3

	Coordinates (Angstroms) X	Y	Z
Ir	-1.374252	-0.373347	-1.226696
C	-2.765591	-1.227199	-0.052754
N	-1.432435	0.920251	0.407047
C	-3.483414	-2.398011	-0.302770
C	-4.427968	-2.881461	0.606618
C	-4.693207	-2.198876	1.805090
C	-3.997331	-1.029879	2.067906
C	-3.046918	-0.542880	1.162520
C	-2.484074	1.552251	2.555478
C	-2.277902	0.663909	1.368682
C	-0.539422	2.027135	0.444201
C	-0.549942	2.961553	-0.592878
C	0.386180	3.988114	-0.607755
C	1.342763	4.072711	0.403185
C	1.352635	3.149187	1.444125
C	0.408790	2.127693	1.461228
O	2.302135	5.061376	0.349217
C	1.952857	6.236403	1.071745
H	-5.423500	-2.563600	2.517918
H	-2.077515	2.551962	2.393330
H	-3.552087	1.643633	2.773047
H	-1.282868	2.876047	-1.389030
H	0.424150	1.381740	2.247282
H	2.781610	6.937679	0.951987
H	1.035253	6.686967	0.671579
H	1.809801	6.019607	2.138229
C	-0.479635	-2.163408	-2.149898
C	-0.813696	-1.225317	-3.173505
C	0.064355	-0.088428	-3.019698
C	0.880803	-0.314095	-1.889265
C	0.564604	-1.613947	-1.338131
C	1.303099	-2.374103	-0.267595
C	1.954452	0.621171	-1.399512
C	1.681094	-1.554634	0.945250
C	3.289432	-0.084008	-1.364138
C	4.093743	-0.114006	-2.536268
C	5.289244	-0.777314	-2.568477
C	5.746005	-1.478144	-1.423314

C	4.948964	-1.481040	-0.243440
C	3.708239	-0.765997	-0.239328
C	0.832095	-1.581066	2.096883
C	1.166858	-0.895942	3.238221
C	2.336639	-0.089709	3.268827
C	3.172916	-0.016922	2.123092
C	2.829516	-0.794845	0.969491
C	2.664275	0.687556	4.410836
C	3.767192	1.506188	4.412488
C	4.594305	1.586430	3.269043
C	4.302558	0.841957	2.151919
H	-0.923303	-3.142645	-2.018703
H	-1.526898	-1.380572	-3.972083
H	0.095317	0.790196	-3.651042
H	0.680137	-3.216133	0.042560
H	2.213447	-2.799117	-0.708273
H	1.990945	1.489845	-2.060381
H	1.712191	0.984291	-0.399728
H	0.538192	-0.928952	4.121717
H	2.019502	0.630610	5.284762
H	4.004262	2.097804	5.292611
H	5.458922	2.244267	3.273297
H	4.935279	0.917332	1.272827
O	3.615888	0.513653	-3.666599
C	4.150550	1.817381	-3.873963
H	5.234814	1.777435	-4.036387
H	3.662901	2.215185	-4.766588
H	3.938386	2.473186	-3.019663
O	-0.286622	-2.335122	1.960936
C	-1.079858	-2.574783	3.109599
H	-1.511322	-1.647652	3.502297
H	-1.883443	-3.236206	2.782990
H	-0.492308	-3.068666	3.893469
H	0.392867	4.722804	-1.407536
H	-2.001066	1.126080	3.442704
H	-4.194953	-0.503711	2.998603
H	-3.335090	-2.962868	-1.219545
O	-5.051391	-4.031848	0.242405
C	-5.997926	-4.604273	1.130001
H	-6.348595	-5.514911	0.641323
H	-5.537567	-4.863615	2.090750
H	-6.848516	-3.932991	1.296306
H	2.109896	3.215611	2.220033
H	5.878144	-0.784336	-3.482013

C	6.973339	-2.193437	-1.435295
C	7.391757	-2.887251	-0.328596
C	5.406462	-2.219350	0.882394
C	6.596615	-2.901603	0.841370
H	8.332163	-3.430878	-0.348544
H	7.573627	-2.183563	-2.341835
H	6.930592	-3.459692	1.711620
H	4.803067	-2.244298	1.784625
H	-2.641211	0.430227	-1.832309
S	-5.447304	1.876080	-0.797309
C	-5.958737	1.877157	0.893592
H	-7.037019	1.699044	0.909870
H	-5.437310	1.072802	1.414832
H	-5.732322	2.850116	1.334004
O	-3.909539	2.300602	-0.667561
H	-3.319302	1.577389	-1.011139
O	-6.128784	2.951031	-1.501652
O	-5.515216	0.524306	-1.334217

A4

	Coordinates (Angstroms)		
	X	Y	Z
Ir	-1.890593	-0.104989	-1.480818
C	-3.521414	-0.281168	-0.309837
N	-1.783956	1.615038	-0.308044
C	-4.424790	-1.346524	-0.256517
C	-5.516951	-1.333392	0.615233
C	-5.756620	-0.235569	1.457503
C	-4.874189	0.832672	1.419004
C	-3.767115	0.816394	0.563416
C	-2.871720	3.101788	1.346561
C	-2.763750	1.857629	0.519428
C	-0.643158	2.457655	-0.414875
C	-0.288512	3.001549	-1.650791
C	0.882611	3.739148	-1.772958
C	1.707652	3.924485	-0.663634
C	1.356026	3.391340	0.572302
C	0.180150	2.657648	0.692482
O	2.896233	4.611599	-0.798573
C	2.756115	6.020935	-0.657704
H	-6.605066	-0.210392	2.131302

H	-2.178506	3.876854	1.016153
H	-3.889667	3.499845	1.285857
H	-0.922288	2.828397	-2.514792
H	-0.088218	2.209456	1.642494
H	3.750751	6.452048	-0.791715
H	2.077174	6.430574	-1.416829
H	2.377060	6.280960	0.339248
C	-1.366280	-2.232234	-1.821387
C	-1.398445	-1.543809	-3.073215
C	-0.295611	-0.613555	-3.080767
C	0.365921	-0.710566	-1.832900
C	-0.274558	-1.731953	-1.041538
C	0.191954	-2.315390	0.267415
C	1.581837	0.090452	-1.445340
C	0.626565	-1.288109	1.288281
C	2.724994	-0.811653	-1.048085
C	3.626022	-1.282859	-2.041723
C	4.661445	-2.120959	-1.730684
C	4.848038	-2.559469	-0.395595
C	3.946292	-2.119666	0.614684
C	2.880362	-1.232884	0.257746
C	-0.312638	-0.840673	2.271623
C	0.046868	0.072063	3.231451
C	1.352087	0.633799	3.227376
C	2.293165	0.223085	2.246069
C	1.903705	-0.773720	1.291904
C	1.723329	1.636721	4.161138
C	2.970285	2.211195	4.118826
C	3.905138	1.807578	3.138737
C	3.572743	0.836984	2.225094
H	-2.032882	-3.031227	-1.520844
H	-2.066052	-1.754913	-3.898102
H	-0.011216	0.043245	-3.892638
H	-0.618862	-2.920287	0.679375
H	1.027749	-2.997798	0.070100
H	1.867368	0.721271	-2.289463
H	1.342938	0.754288	-0.612634
H	-0.655497	0.400544	3.990095
H	0.997134	1.950655	4.907123
H	3.240116	2.980881	4.836718
H	4.885958	2.273140	3.104025
H	4.292088	0.541065	1.467897
O	3.414531	-0.895079	-3.347894
C	4.261801	0.166466	-3.776911

H	5.318405	-0.123793	-3.723301
H	3.997742	0.378175	-4.815409
H	4.105249	1.067496	-3.169915
O	-1.542211	-1.402989	2.175747
C	-2.495448	-1.117238	3.183111
H	-2.733170	-0.048701	3.219771
H	-3.391863	-1.676943	2.912512
H	-2.138132	-1.449526	4.165945
H	1.174340	4.160558	-2.730630
H	-2.665347	2.882812	2.400605
H	-5.052143	1.679898	2.076575
H	-4.304664	-2.218174	-0.895088
O	-6.308091	-2.440075	0.580368
C	-7.433361	-2.491425	1.441858
H	-7.904241	-3.459801	1.262968
H	-7.135600	-2.427736	2.495304
H	-8.150617	-1.694847	1.211475
H	2.012160	3.529571	1.426787
H	5.333879	-2.462236	-2.513468
C	5.908940	-3.437979	-0.046600
C	6.066598	-3.868661	1.245632
C	4.131086	-2.594685	1.942342
C	5.164742	-3.443253	2.249421
H	6.881243	-4.539949	1.502943
H	6.593970	-3.765138	-0.825053
H	5.291390	-3.795195	3.269497
H	3.441263	-2.280870	2.719562
H	-2.875766	0.793872	-2.387795

A5fR

	Coordinates (Angstroms) X	Y	Z
Ir	-0.019178	-0.210782	0.131227
C	0.708002	-2.039699	-0.259230
N	-0.645396	-0.525955	-1.828429
C	1.404278	-2.879989	0.613852
C	1.866506	-4.130624	0.200629
C	1.624846	-4.601527	-1.099603
C	0.950863	-3.775902	-1.985982
C	0.495369	-2.516150	-1.583248
C	-0.542615	-1.929326	-3.867177

C	-0.259720	-1.618940	-2.429079
C	-1.506793	0.422965	-2.444409
C	-1.131837	1.766991	-2.533821
C	-2.022977	2.706379	-3.025019
C	-3.310287	2.321919	-3.423014
C	-3.683543	0.980285	-3.353995
C	-2.776558	0.042881	-2.863779
O	-4.122237	3.325269	-3.855342
C	-5.450915	2.990978	-4.220832
H	1.965350	-5.578202	-1.423588
H	-0.859213	-1.044645	-4.422682
H	0.355760	-2.334910	-4.342460
H	-0.146728	2.073634	-2.196389
H	-3.076533	-0.995329	-2.775958
H	-5.932076	3.931987	-4.493094
H	-5.471600	2.312254	-5.082411
H	-5.991187	2.534463	-3.382523
C	-0.363270	-0.369461	2.311058
C	0.410011	0.799466	2.033219
C	-0.447698	1.719406	1.323255
C	-1.704417	1.094593	1.135788
C	-1.675785	-0.203954	1.764121
C	-2.832058	-1.138816	2.000739
C	-2.880273	1.713264	0.422896
C	-3.685162	-1.384243	0.778224
C	-4.119618	1.720167	1.285851
C	-4.366700	2.828662	2.142080
C	-5.472202	2.881935	2.945956
C	-6.392812	1.804186	2.962991
C	-6.153098	0.669000	2.138034
C	-4.998023	0.653810	1.291368
C	-3.405669	-2.524874	-0.039707
C	-4.167973	-2.805679	-1.145406
C	-5.220560	-1.930199	-1.524912
C	-5.498544	-0.776580	-0.744251
C	-4.715496	-0.538058	0.433733
C	-5.977195	-2.164498	-2.702911
C	-6.965529	-1.293308	-3.091235
C	-7.240548	-0.142893	-2.317499
C	-6.521640	0.109048	-1.173356
H	-0.022667	-1.231185	2.870738
H	1.416702	0.987283	2.383527
H	-0.189032	2.716458	0.990450
H	-2.435736	-2.089046	2.366043

H	-3.460515	-0.727925	2.800277
H	-2.615314	2.732480	0.134197
H	-3.090338	1.163039	-0.496696
H	-3.974958	-3.679000	-1.759319
H	-5.755720	-3.047259	-3.298314
H	-7.535209	-1.483936	-3.996737
H	-8.019640	0.546198	-2.631312
H	-6.729919	1.002835	-0.593073
O	-3.437978	3.846968	2.162201
C	-3.827779	5.003827	1.430318
H	-4.730865	5.457991	1.856453
H	-3.000234	5.713141	1.499239
H	-4.012713	4.761745	0.375584
O	-2.365201	-3.283338	0.381609
C	-2.079572	-4.481437	-0.316361
H	-1.832388	-4.284775	-1.364848
H	-1.212367	-4.916706	0.181870
H	-2.924309	-5.179641	-0.261549
H	-1.744463	3.753883	-3.093508
H	-1.330705	-2.686660	-3.950880
H	0.772109	-4.131377	-2.997837
H	1.633594	-2.571497	1.629117
O	2.570243	-4.829233	1.132851
C	3.019040	-6.131827	0.803695
H	3.531125	-6.503314	1.693069
H	2.179298	-6.796450	0.566809
H	3.725135	-6.116949	-0.035506
H	-4.670710	0.646930	-3.653472
H	-5.635272	3.744448	3.586938
C	-7.540044	1.826272	3.801010
C	-8.410979	0.767327	3.826012
C	-7.068563	-0.417586	2.198536
C	-8.168430	-0.368234	3.017624
H	-9.285306	0.794000	4.470641
H	-7.713564	2.700264	4.424032
H	-8.858801	-1.206620	3.049105
H	-6.889913	-1.298950	1.590712
H	1.313313	0.259733	-0.618818
C	6.163863	2.180663	0.295146
C	7.117920	3.161369	0.057386
C	6.693056	4.374300	-0.465138
C	5.355153	4.619674	-0.735147
C	4.434594	3.609620	-0.483032
C	4.799000	2.356667	0.028482

H	8.163537	2.981125	0.277589
H	5.031256	5.574929	-1.131045
Cl	2.757492	3.979925	-0.819874
Cl	7.868094	5.623783	-0.782543
Cl	6.726603	0.674035	0.977850
C	3.797851	1.255448	0.289005
H	2.787248	1.670776	0.327953
H	3.977751	0.787468	1.261084
N	3.960843	-0.999033	-0.477783
C	3.784969	0.230447	-0.810690
C	3.581375	0.628860	-2.225924
H	2.895478	1.477197	-2.272704
H	4.543302	0.951200	-2.644000
H	3.193678	-0.197489	-2.821386
O	3.854687	-1.987238	-1.407593
C	5.128735	-2.690566	-1.675124
C	6.128948	-1.709162	-2.270863
H	5.743674	-1.270993	-3.197145
H	6.374198	-0.904949	-1.569567
H	7.060085	-2.237035	-2.501064
C	4.700965	-3.731994	-2.697583
H	5.586311	-4.251983	-3.076035
H	4.027866	-4.468201	-2.250522
H	4.190694	-3.256969	-3.542559
C	5.640555	-3.332284	-0.391869
H	6.033874	-2.589650	0.309716
H	4.841924	-3.890088	0.108245
H	6.454055	-4.023980	-0.633912
H	4.031480	-1.313039	0.522371
S	3.717662	-1.235500	3.390023
C	4.887702	-1.794216	4.611291
H	4.883626	-2.886845	4.627643
H	5.881276	-1.427495	4.342764
H	4.595495	-1.403823	5.589098
O	3.777247	0.245277	3.388908
O	2.399239	-1.768069	3.794086
O	4.212154	-1.825553	2.108196

A5fS

Coordinates (Angstroms)

X Y Z

Ir	0.257158	0.239781	0.293573
C	-0.963370	-0.367602	-1.172833
N	0.571707	-1.816802	0.281712
C	-1.761692	0.436445	-1.990187
C	-2.572764	-0.118150	-2.982077
C	-2.615203	-1.506203	-3.192255
C	-1.833434	-2.321708	-2.386957
C	-1.014526	-1.772828	-1.393961
C	-0.062826	-4.038220	-0.609967
C	-0.134323	-2.543710	-0.541450
C	1.558929	-2.370845	1.144963
C	1.488528	-2.151749	2.517820
C	2.500868	-2.599457	3.362923
C	3.606324	-3.264602	2.826359
C	3.677803	-3.492593	1.448902
C	2.660025	-3.049755	0.618456
O	4.658112	-3.723342	3.557403
C	4.661878	-3.476902	4.953130
H	-3.248622	-1.945955	-3.953960
H	0.401554	-4.468748	0.279041
H	-1.066908	-4.459890	-0.714296
H	0.641290	-1.613795	2.932017
H	2.731265	-3.202178	-0.452515
H	5.594287	-3.902288	5.328657
H	4.642542	-2.401494	5.168234
H	3.816314	-3.968273	5.450017
C	0.830729	2.235044	-0.488212
C	0.343457	2.367613	0.847523
C	1.306490	1.735668	1.717866
C	2.328648	1.180818	0.912962
C	2.058666	1.504889	-0.467380
C	2.968762	1.318791	-1.652164
C	3.531516	0.427143	1.416662
C	3.635394	-0.035455	-1.735972
C	4.804570	1.104650	0.970740
C	5.330143	2.172538	1.760580
C	6.471692	2.833077	1.387257
C	7.132245	2.480048	0.179768
C	6.605602	1.446244	-0.642249
C	5.421295	0.760490	-0.211132
C	3.033248	-1.057709	-2.536502
C	3.640095	-2.277637	-2.700377
C	4.842655	-2.573260	-2.003941
C	5.427467	-1.593746	-1.157578

C	4.810431	-0.303022	-1.067828
C	5.447895	-3.853943	-2.097721
C	6.576601	-4.153764	-1.374704
C	7.149659	-3.185093	-0.519644
C	6.587842	-1.935789	-0.415062
H	0.360979	2.646103	-1.372757
H	-0.532991	2.924097	1.146213
H	1.257125	1.678973	2.797941
H	2.386843	1.493395	-2.560107
H	3.745529	2.092933	-1.620406
H	3.488518	0.367807	2.506353
H	3.518009	-0.595551	1.035243
H	3.205109	-3.047308	-3.329105
H	4.996870	-4.598903	-2.749113
H	7.029812	-5.138083	-1.454714
H	8.035609	-3.432173	0.058621
H	7.026979	-1.200939	0.252734
O	4.602866	2.460364	2.871867
C	5.015418	3.554794	3.671418
H	5.021160	4.487406	3.094416
H	4.280272	3.632012	4.474074
H	6.008053	3.383132	4.104901
O	1.854574	-0.709501	-3.108767
C	1.229162	-1.630850	-3.983048
H	0.987332	-2.569338	-3.471952
H	0.306804	-1.148931	-4.310084
H	1.864389	-1.838487	-4.853245
H	2.417784	-2.413701	4.427920
H	0.517398	-4.353650	-1.485468
H	-1.864840	-3.396202	-2.548922
H	-1.791817	1.512411	-1.846605
O	-3.322231	0.769668	-3.684595
C	-4.195076	0.284867	-4.689626
H	-4.690629	1.165608	-5.101862
H	-3.644577	-0.226552	-5.488476
H	-4.950972	-0.390472	-4.271194
H	4.546269	-4.001834	1.040939
H	6.882898	3.638350	1.986842
C	8.307583	3.160212	-0.234417
C	8.928304	2.838494	-1.415654
C	7.262177	1.146032	-1.865169
C	8.396419	1.823229	-2.242478
H	9.826896	3.367445	-1.721163
H	8.706580	3.946799	0.401590

H	8.886127	1.580673	-3.181407
H	6.857303	0.371838	-2.509878
H	-0.977750	-0.141230	1.240962
C	-5.967858	-2.512878	0.153865
C	-7.153032	-3.166532	0.469254
C	-8.284662	-2.401731	0.709754
C	-8.252357	-1.017196	0.634465
C	-7.047482	-0.404813	0.314124
C	-5.866073	-1.117410	0.064212
H	-7.188400	-4.248208	0.525868
H	-9.141009	-0.425022	0.819638
Cl	-7.049344	1.338993	0.216287
Cl	-9.781247	-3.200084	1.114709
Cl	-4.574338	-3.526651	-0.142217
C	-4.574407	-0.410036	-0.282487
H	-4.751822	0.395290	-0.999868
H	-3.884127	-1.112532	-0.760048
N	-3.549865	1.359407	0.943900
C	-3.858575	0.111316	0.934086
C	-3.515000	-0.805221	2.047913
H	-2.959488	-1.656317	1.641247
H	-2.920814	-0.315467	2.816323
H	-4.440861	-1.194484	2.488006
O	-2.784908	1.879469	1.939355
C	-3.490442	2.835975	2.822204
C	-3.966383	4.033845	2.013772
H	-3.136334	4.480203	1.459038
H	-4.758340	3.766973	1.306749
H	-4.373774	4.788233	2.694959
C	-2.378194	3.215500	3.788601
H	-2.768426	3.904767	4.543804
H	-1.985664	2.328272	4.296634
H	-1.556859	3.712263	3.261930
C	-4.634997	2.123474	3.529680
H	-5.392706	1.771996	2.821114
H	-4.269976	1.272042	4.112800
H	-5.124903	2.822962	4.214628
H	-3.754855	2.001004	0.131533
S	-3.329033	4.035194	-1.551743
C	-3.132992	4.008803	-3.320302
H	-2.646068	3.078067	-3.616308
H	-4.117750	4.078811	-3.789204
H	-2.522113	4.866918	-3.611921
O	-3.983117	5.315866	-1.217584

O	-1.975227	3.885579	-0.970756
O	-4.193495	2.852001	-1.227894

A5uR

	Coordinates (Angstroms) X	Y	Z
Ir	0.477976	0.782094	-0.904802
C	1.239728	2.630753	-0.699131
N	0.426422	1.056442	1.159773
C	1.619658	3.511999	-1.715190
C	2.182023	4.758468	-1.427399
C	2.397366	5.164195	-0.100044
C	2.026213	4.305394	0.923217
C	1.447559	3.062832	0.640523
C	1.110371	2.418094	3.111639
C	0.972972	2.138986	1.646146
C	-0.260254	0.126631	1.988160
C	0.131715	-1.212508	2.033276
C	-0.601882	-2.129932	2.767027
C	-1.752724	-1.725714	3.455235
C	-2.147193	-0.388577	3.420057
C	-1.392992	0.526169	2.688552
O	-2.415707	-2.707252	4.122665
C	-3.625412	-2.362631	4.778906
H	2.845515	6.122542	0.134423
H	0.962779	1.520685	3.715030
H	2.107701	2.818210	3.319233
H	1.007695	-1.522660	1.474674
H	-1.711564	1.561457	2.635678
H	-4.002996	-3.288318	5.216512
H	-3.455252	-1.629343	5.576590
H	-4.361902	-1.968237	4.068801
C	-0.477992	0.932427	-2.902986
C	0.391594	-0.200241	-2.869762
C	-0.188067	-1.157600	-1.958555
C	-1.363890	-0.585545	-1.411650
C	-1.576377	0.705400	-2.015443
C	-2.796274	1.581461	-1.922508
C	-2.262315	-1.252915	-0.401087
C	-3.337979	1.756331	-0.523832
C	-3.669968	-1.381752	-0.927910

C	-4.032680	-2.543308	-1.663653
C	-5.279265	-2.686538	-2.207108
C	-6.245354	-1.660152	-2.053919
C	-5.907113	-0.486731	-1.322044
C	-4.597489	-0.371896	-0.754585
C	-2.925695	2.891551	0.243387
C	-3.442401	3.124996	1.492727
C	-4.346296	2.195347	2.073581
C	-4.724645	1.031283	1.352273
C	-4.214298	0.846868	0.024504
C	-4.847999	2.386275	3.388104
C	-5.684077	1.461357	3.964528
C	-6.053192	0.298134	3.251642
C	-5.581441	0.088405	1.978093
H	-0.350455	1.810173	-3.524729
H	1.266979	-0.347546	-3.488012
H	0.206282	-2.136293	-1.722373
H	-2.551897	2.557829	-2.346903
H	-3.583020	1.151143	-2.554450
H	-1.851637	-2.236959	-0.167848
H	-2.274918	-0.676605	0.525946
H	-3.153311	3.995761	2.071371
H	-4.555879	3.280339	3.933957
H	-6.062722	1.620600	4.970462
H	-6.712980	-0.431285	3.713014
H	-5.863675	-0.812708	1.442633
O	-3.086013	-3.533936	-1.833532
C	-3.222578	-4.611317	-0.910775
H	-4.203844	-5.091567	-1.010559
H	-2.442401	-5.334658	-1.154260
H	-3.097280	-4.264475	0.123419
O	-2.014029	3.686025	-0.369406
C	-1.536322	4.826180	0.321768
H	-1.073963	4.548345	1.275495
H	-0.784226	5.274231	-0.329350
H	-2.344027	5.547219	0.499159
H	-0.311141	-3.174877	2.814067
H	0.380696	3.170642	3.432434
H	2.186312	4.618894	1.951677
H	1.498212	3.247898	-2.762753
O	2.500933	5.517152	-2.509721
C	3.100238	6.785093	-2.299529
H	3.247226	7.213605	-3.292697
H	2.448080	7.445752	-1.716153

H	4.071185	6.688717	-1.799362
H	-3.037242	-0.043163	3.934370
H	-5.525559	-3.586635	-2.764373
C	-7.542758	-1.777659	-2.620578
C	-8.465560	-0.773825	-2.473625
C	-6.883719	0.539339	-1.197099
C	-8.128897	0.398576	-1.756625
H	-9.454797	-0.873247	-2.911552
H	-7.790366	-2.679784	-3.174796
H	-8.862680	1.192972	-1.652322
H	-6.635168	1.447262	-0.656210
H	2.005241	0.389282	-0.625257
C	6.383818	-2.013606	0.622903
C	7.731308	-1.703831	0.503282
C	8.087182	-0.363590	0.437175
C	7.136749	0.644814	0.494810
C	5.797682	0.290813	0.614640
C	5.374123	-1.043988	0.677356
H	8.479766	-2.486987	0.465354
H	7.427238	1.687768	0.446829
Cl	4.640990	1.597605	0.698720
Cl	9.770310	0.063940	0.282260
Cl	5.954804	-3.707470	0.713990
C	3.922735	-1.430198	0.819036
H	3.310847	-0.530386	0.949496
H	3.771287	-2.042220	1.714829
N	2.498234	-3.054451	-0.164757
C	3.352196	-2.118071	-0.387881
C	3.707760	-1.723766	-1.770713
H	3.909039	-0.651414	-1.814438
H	4.628585	-2.252547	-2.052073
H	2.926564	-1.989057	-2.482464
O	1.800685	-3.626678	-1.169687
C	1.188529	-4.933366	-0.830976
C	2.303683	-5.940540	-0.599684
H	2.975822	-5.978414	-1.462749
H	2.888116	-5.691441	0.293347
H	1.871735	-6.934443	-0.446804
C	0.396423	-5.217305	-2.097844
H	-0.075531	-6.200445	-2.013343
H	-0.385808	-4.465156	-2.246624
H	1.054222	-5.223742	-2.972932
C	0.269120	-4.778671	0.375347
H	0.813839	-4.668349	1.321308

H	-0.404519	-3.926472	0.242550
H	-0.335876	-5.685218	0.473208
H	2.244367	-3.338000	0.782591

A5uS

	Coordinates (Angstroms) X	Y	Z
Ir	-0.181372	-0.611013	-0.478677
C	-1.184457	-1.186417	1.157119
N	-0.115655	1.099704	0.723059
C	-1.683667	-2.463394	1.446833
C	-2.424998	-2.718770	2.605924
C	-2.701288	-1.692025	3.523256
C	-2.189299	-0.427429	3.272586
C	-1.432472	-0.171794	2.124192
C	-0.947035	2.285491	2.732823
C	-0.803275	1.098969	1.830976
C	0.692067	2.199546	0.323532
C	0.490596	2.803620	-0.918726
C	1.349857	3.807444	-1.348207
C	2.421426	4.197260	-0.544064
C	2.622191	3.603308	0.698028
C	1.756341	2.603352	1.128729
O	3.313840	5.147806	-0.991706
C	2.863549	6.486422	-0.814233
H	-3.280722	-1.872105	4.421067
H	-0.621459	3.210004	2.253465
H	-1.993602	2.399871	3.032253
H	-0.322599	2.468231	-1.554963
H	1.919812	2.110819	2.081000
H	3.645541	7.136675	-1.212389
H	1.928091	6.667823	-1.358567
H	2.708918	6.708856	0.249541
C	0.668611	-2.544265	-1.132886
C	0.055566	-1.896294	-2.248303
C	0.844471	-0.727949	-2.551969
C	1.895701	-0.650375	-1.607197
C	1.817095	-1.788181	-0.725320
C	2.822065	-2.234033	0.305402
C	2.938983	0.434606	-1.566120
C	3.319824	-1.127804	1.208499

C	4.328967	-0.147920	-1.639588
C	4.919982	-0.385863	-2.910442
C	6.170200	-0.927423	-3.028619
C	6.898488	-1.295247	-1.868622
C	6.314591	-1.097615	-0.585149
C	5.012477	-0.509177	-0.495872
C	2.686120	-0.925792	2.476156
C	3.113123	0.058004	3.332289
C	4.169179	0.927549	2.948747
C	4.798294	0.759636	1.686688
C	4.362764	-0.308983	0.835773
C	4.582993	1.994027	3.789487
C	5.573084	2.859425	3.392794
C	6.193864	2.698192	2.133375
C	5.813972	1.673135	1.301304
H	0.348143	-3.479607	-0.690911
H	-0.784536	-2.267458	-2.818771
H	0.675678	-0.027778	-3.359929
H	2.365013	-3.021612	0.908748
H	3.677685	-2.686097	-0.211029
H	2.764878	1.121401	-2.396680
H	2.839471	1.011662	-0.644758
H	2.648395	0.206969	4.301105
H	4.095530	2.119447	4.753429
H	5.878355	3.672992	4.045247
H	6.969152	3.392031	1.821205
H	6.288931	1.563775	0.331288
O	4.177911	-0.088668	-4.033754
C	4.508975	1.159877	-4.633334
H	5.537298	1.155253	-5.015228
H	3.813323	1.301878	-5.463215
H	4.395695	1.983550	-3.916759
O	1.670677	-1.782833	2.745260
C	1.047787	-1.713471	4.015198
H	0.578871	-0.737042	4.177704
H	0.278736	-2.487120	4.010004
H	1.768447	-1.916005	4.817158
H	1.208338	4.277532	-2.317040
H	-0.358350	2.144475	3.646632
H	-2.381300	0.362736	3.993793
H	-1.507994	-3.296063	0.770287
O	-2.852013	-3.998488	2.750329
C	-3.625632	-4.336911	3.891058
H	-3.850271	-5.399948	3.790153

H	-3.064188	-4.172035	4.817800
H	-4.563094	-3.769070	3.920463
H	3.465060	3.909087	1.310989
H	6.599290	-1.093618	-4.013211
C	8.191439	-1.876295	-1.962680
C	8.874849	-2.252386	-0.834568
C	7.045394	-1.508290	0.563393
C	8.292107	-2.069010	0.441403
H	9.862299	-2.697770	-0.917688
H	8.629092	-2.019143	-2.947697
H	8.835858	-2.376969	1.330142
H	6.604835	-1.379565	1.547200
H	-1.587430	0.011632	-0.939257
C	-5.022061	2.593385	-0.229639
C	-5.981176	3.576536	-0.441426
C	-7.315391	3.198629	-0.485262
C	-7.703697	1.875816	-0.322518
C	-6.712813	0.927397	-0.112052
C	-5.349257	1.242170	-0.057219
H	-5.690741	4.612608	-0.570152
H	-8.748320	1.589377	-0.356919
Cl	-7.216143	-0.735391	0.089895
Cl	-8.534849	4.415979	-0.750814
Cl	-3.352402	3.114628	-0.182267
C	-4.310928	0.180354	0.191568
H	-4.559772	-0.405571	1.083507
H	-3.341748	0.647838	0.397651
N	-3.734558	-1.934301	-0.707805
C	-4.057569	-0.719908	-0.983483
C	-4.120064	-0.225944	-2.377035
H	-5.174290	-0.152184	-2.674468
H	-3.694727	0.780348	-2.427142
H	-3.598619	-0.889755	-3.065050
O	-3.232000	-2.780151	-1.636030
C	-4.002627	-4.040983	-1.769899
C	-4.146930	-4.711358	-0.409328
H	-3.171975	-4.847307	0.068188
H	-4.803462	-4.149155	0.264321
H	-4.599516	-5.697982	-0.549076
C	-3.105880	-4.843766	-2.697960
H	-3.569149	-5.814009	-2.900329
H	-2.965143	-4.324230	-3.651356
H	-2.127322	-5.015318	-2.237537
C	-5.347063	-3.718542	-2.403794

H	-5.954800	-3.077734	-1.755517
H	-5.213393	-3.223076	-3.370678
H	-5.903513	-4.647509	-2.565676
H	-3.587628	-2.237060	0.260676

A6

	Coordinates (Angstroms) X	Y	Z
Ir	-1.823187	-0.282188	-1.418303
C	-3.488197	-0.349893	-0.248046
N	-1.775572	1.605234	-0.510097
C	-4.366305	-1.408017	-0.086141
C	-5.519300	-1.276008	0.711671
C	-5.805518	-0.068752	1.354756
C	-4.931695	1.002768	1.193370
C	-3.788600	0.871582	0.408406
C	-2.961478	3.265039	0.877539
C	-2.799456	1.928837	0.232823
C	-0.631002	2.441056	-0.656085
C	-0.224413	2.850166	-1.925904
C	0.955365	3.569090	-2.074398
C	1.733116	3.867062	-0.954987
C	1.325353	3.468225	0.314475
C	0.141948	2.753014	0.461067
O	2.933674	4.524472	-1.102079
C	2.808148	5.938130	-1.214310
H	-6.690252	0.050259	1.968885
H	-2.271448	4.006265	0.472828
H	-3.986493	3.620431	0.737269
H	-0.821917	2.590948	-2.795548
H	-0.172336	2.408775	1.440598
H	3.819416	6.333579	-1.331771
H	2.205858	6.217563	-2.088163
H	2.353228	6.364251	-0.310879
C	-1.403745	-2.391883	-1.640604
C	-1.420906	-1.830141	-2.933983
C	-0.298660	-0.912936	-3.049859
C	0.361759	-0.852222	-1.821002
C	-0.363716	-1.724256	-0.883821
C	0.078701	-2.164451	0.484233
C	1.578920	-0.033188	-1.498627

C	0.571879	-1.061734	1.391362
C	2.688246	-0.923507	-0.992603
C	3.568114	-1.534054	-1.927128
C	4.569820	-2.372108	-1.521450
C	4.734931	-2.669870	-0.145088
C	3.849383	-2.090206	0.807765
C	2.822530	-1.202303	0.352234
C	-0.333671	-0.503728	2.348769
C	0.075646	0.472519	3.221468
C	1.396772	0.987617	3.145107
C	2.305473	0.465328	2.186805
C	1.868015	-0.599425	1.332321
C	1.817159	2.049708	3.988043
C	3.079945	2.577465	3.876623
C	3.981330	2.066297	2.915148
C	3.602602	1.034210	2.091571
H	-2.069409	-3.152309	-1.254424
H	-2.117741	-2.073644	-3.725686
H	-0.047484	-0.334541	-3.929968
H	-0.755703	-2.686898	0.956406
H	0.875343	-2.903425	0.334724
H	1.888560	0.502667	-2.397764
H	1.336495	0.712510	-0.739001
H	-0.597979	0.886573	3.963941
H	1.116136	2.443615	4.720141
H	3.388663	3.392154	4.525841
H	4.974627	2.496540	2.824780
H	4.298356	0.654716	1.349638
O	3.358986	-1.284874	-3.265726
C	4.263842	-0.338701	-3.828321
H	5.298274	-0.698511	-3.771621
H	3.978573	-0.218345	-4.875609
H	4.190033	0.628715	-3.315646
O	-1.582478	-1.028788	2.321991
C	-2.529668	-0.568182	3.268905
H	-2.708062	0.507865	3.164857
H	-3.452047	-1.109828	3.053977
H	-2.204224	-0.790994	4.292325
H	1.290445	3.885071	-3.058019
H	-2.787152	3.179404	1.956615
H	-5.158622	1.937754	1.697741
H	-4.201382	-2.367161	-0.568902
O	-6.293291	-2.380801	0.789328
C	-7.458573	-2.340089	1.601539

H	-7.912507	-3.328559	1.517494
H	-7.206468	-2.143021	2.649773
H	-8.167273	-1.585616	1.241056
H	1.944170	3.700036	1.176384
H	5.228915	-2.824740	-2.257613
C	5.755853	-3.551490	0.301027
C	5.890225	-3.851938	1.632200
C	4.009178	-2.431721	2.178863
C	5.003288	-3.288469	2.579505
H	6.673288	-4.528303	1.963157
H	6.429051	-3.984735	-0.434545
H	5.111494	-3.537362	3.631429
H	3.330985	-2.012932	2.915733

A7fR

	Coordinates (Angstroms) X	Y	Z
Ir	0.134969	0.113874	0.529147
C	0.943447	-0.902994	2.061452
N	-0.189297	-1.866589	-0.030979
C	1.527886	-0.378143	3.218504
C	2.069084	-1.209726	4.202222
C	2.058964	-2.606337	4.057341
C	1.492001	-3.147065	2.913684
C	0.935859	-2.320843	1.930951
C	0.189520	-4.272445	0.421191
C	0.287327	-2.810020	0.734237
C	-0.948883	-2.138692	-1.202275
C	-0.554476	-1.607926	-2.434338
C	-1.356203	-1.773138	-3.551374
C	-2.575173	-2.457202	-3.453596
C	-2.965430	-3.004769	-2.232179
C	-2.145422	-2.841083	-1.117224
O	-3.309667	-2.527931	-4.597273
C	-4.584996	-3.143461	-4.526581
H	2.482232	-3.260036	4.811110
H	-0.035318	-4.455291	-0.631381
H	1.128421	-4.776286	0.668299
H	0.372404	-1.047352	-2.506426
H	-2.462772	-3.237971	-0.159476
H	-5.021754	-3.043502	-5.521825

H	-4.503333	-4.207607	-4.273630
H	-5.229816	-2.640831	-3.795586
C	-0.583308	1.944034	1.554990
C	0.216505	2.308007	0.430318
C	-0.504282	1.919157	-0.757231
C	-1.707845	1.293664	-0.349852
C	-1.783137	1.317729	1.090306
C	-2.958342	0.949182	1.957393
C	-2.761487	0.744460	-1.275077
C	-3.630579	-0.353751	1.586699
C	-4.098164	1.390378	-1.001772
C	-4.407155	2.632194	-1.636762
C	-5.603211	3.263137	-1.411117
C	-6.543002	2.699781	-0.506226
C	-6.238792	1.486256	0.169017
C	-4.988113	0.840402	-0.106787
C	-3.239348	-1.552600	2.263563
C	-3.856285	-2.749136	1.996394
C	-4.853345	-2.826969	0.987562
C	-5.227698	-1.657975	0.272587
C	-4.608360	-0.411890	0.617576
C	-5.452717	-4.067328	0.643880
C	-6.380965	-4.145915	-0.365394
C	-6.750382	-2.984322	-1.081205
C	-6.184352	-1.771244	-0.770157
H	-0.338109	2.141302	2.590987
H	1.135314	2.870272	0.484080
H	-0.192900	2.072260	-1.782089
H	-2.616656	0.898908	2.993931
H	-3.699874	1.756227	1.907494
H	-2.458425	0.917865	-2.309769
H	-2.851814	-0.334471	-1.139132
H	-3.582682	-3.657905	2.521992
H	-5.159148	-4.959271	1.192583
H	-6.832940	-5.101458	-0.616855
H	-7.484142	-3.052095	-1.879565
H	-6.465232	-0.885845	-1.332433
O	-3.425689	3.105327	-2.448178
C	-3.622098	4.358300	-3.079560
H	-3.770276	5.155374	-2.341094
H	-2.707400	4.553046	-3.642267
H	-4.473924	4.329596	-3.769886
O	-2.244884	-1.393524	3.170898
C	-1.840913	-2.518341	3.930193

H	-1.474781	-3.325107	3.285955
H	-1.028004	-2.171229	4.569390
H	-2.665034	-2.887673	4.553027
H	-1.063227	-1.358311	-4.511322
H	-0.600238	-4.739079	1.022091
H	1.478499	-4.228092	2.799528
H	1.599830	0.694263	3.374197
O	2.600774	-0.568792	5.279145
C	3.173041	-1.344732	6.317793
H	3.517356	-0.629834	7.067279
H	2.434442	-2.016498	6.771801
H	4.028897	-1.928192	5.957363
H	-3.903862	-3.536329	-2.118894
H	-5.850203	4.201073	-1.896993
C	-7.783113	3.340793	-0.246985
C	-8.677251	2.809235	0.648973
C	-7.177408	0.968678	1.100548
C	-8.368640	1.611982	1.333771
H	-9.622740	3.310583	0.837348
H	-8.011167	4.266958	-0.769238
H	-9.076113	1.202217	2.049343
H	-6.946167	0.052007	1.634738
H	1.573811	-0.135105	-0.116130
C	6.290080	-0.696863	-1.911664
C	7.317172	-1.624040	-1.803834
C	6.977153	-2.949633	-1.573231
C	5.654446	-3.350453	-1.459036
C	4.658684	-2.386984	-1.573066
C	4.934064	-1.032440	-1.798362
H	8.352646	-1.318218	-1.898637
H	5.400127	-4.389394	-1.285002
Cl	3.008214	-2.941173	-1.434096
Cl	8.242926	-4.141025	-1.426713
Cl	6.741743	0.967590	-2.204290
C	3.858006	0.013419	-1.935922
H	2.869292	-0.454152	-2.005756
H	3.986725	0.579259	-2.863415
N	3.270790	2.129348	-0.964554
C	3.796763	0.971548	-0.783329
C	4.278404	0.626101	0.584429
H	4.082727	-0.431995	0.778295
H	5.360156	0.789736	0.654482
H	3.768130	1.220394	1.345094
O	2.659451	2.407267	-2.142963

C	2.792113	3.806743	-2.609107
C	2.305978	3.686411	-4.045845
H	2.310318	4.674750	-4.515140
H	2.955545	3.023307	-4.626618
H	1.283635	3.295074	-4.078059
C	1.874747	4.702956	-1.786024
H	2.188045	4.765029	-0.739969
H	1.905426	5.716609	-2.199546
H	0.841265	4.345019	-1.831148
C	4.250447	4.235431	-2.537212
H	4.353283	5.228020	-2.987089
H	4.606379	4.303089	-1.503700
H	4.889084	3.541602	-3.093556
H	3.244954	2.848809	-0.199025
S	3.301501	4.010339	2.404520
C	4.835746	3.768012	3.275558
H	5.524457	4.575686	3.016663
H	4.638449	3.775879	4.350327
H	5.261512	2.805156	2.983310
O	2.431542	2.870729	2.766586
O	3.679897	3.976263	0.952502
O	2.777236	5.327896	2.807511

A7fS

	Coordinates (Angstroms)		
	X	Y	Z
Ir	-0.141913	0.407611	-0.142245
C	0.827642	0.411883	1.616185
N	-0.403627	-1.551658	0.514769
C	1.460144	1.493903	2.233594
C	2.108831	1.350623	3.461388
C	2.171392	0.108265	4.111480
C	1.556326	-0.979990	3.511976
C	0.882250	-0.839042	2.293790
C	0.121764	-3.303025	2.182652
C	0.174775	-1.911050	1.628812
C	-1.226599	-2.434496	-0.238355
C	-0.965745	-2.653197	-1.588005
C	-1.825929	-3.425587	-2.363932
C	-2.970009	-3.981418	-1.785184
C	-3.229016	-3.775547	-0.426647

C	-2.363396	-3.007768	0.336676
O	-3.886300	-4.729210	-2.458769
C	-3.706618	-4.905838	-3.853890
H	2.681822	-0.011947	5.060052
H	-0.115096	-4.040563	1.413440
H	1.078570	-3.572334	2.638496
H	-0.091036	-2.201173	-2.045177
H	-2.584546	-2.820714	1.381193
H	-4.562363	-5.491656	-4.193855
H	-3.696353	-3.943292	-4.379848
H	-2.783401	-5.455730	-4.073536
C	-0.844829	2.508268	-0.252885
C	-0.220488	2.157276	-1.486922
C	-1.052195	1.167499	-2.124402
C	-2.150309	0.901056	-1.267942
C	-2.048197	1.744306	-0.103678
C	-3.061893	1.938272	0.995330
C	-3.256334	-0.077534	-1.566128
C	-3.637894	0.651503	1.543028
C	-4.612235	0.579769	-1.483955
C	-5.162567	1.203262	-2.636970
C	-6.381538	1.822832	-2.604098
C	-7.118551	1.880703	-1.393746
C	-6.574356	1.291941	-0.217165
C	-5.305436	0.633027	-0.291350
C	-3.063111	0.073094	2.720493
C	-3.563264	-1.088404	3.254445
C	-4.642638	-1.759360	2.619225
C	-5.218506	-1.212145	1.441898
C	-4.702776	0.025478	0.934164
C	-5.135793	-2.993679	3.118213
C	-6.151880	-3.657715	2.475408
C	-6.721732	-3.116838	1.300611
C	-6.263855	-1.923377	0.796814
H	-0.487472	3.259044	0.440419
H	0.657197	2.630479	-1.903941
H	-0.892200	0.715645	-3.094964
H	-2.582632	2.495302	1.803548
H	-3.879432	2.565143	0.618815
H	-3.092370	-0.500114	-2.559383
H	-3.221381	-0.905730	-0.856470
H	-3.141049	-1.524811	4.153421
H	-4.689106	-3.410543	4.017866
H	-6.518517	-4.602569	2.867498

H	-7.520832	-3.649854	0.792970
H	-6.699838	-1.521398	-0.112535
O	-4.412109	1.204428	-3.793535
C	-4.789131	0.206902	-4.736315
H	-5.814851	0.364163	-5.092184
H	-4.094482	0.295963	-5.574620
H	-4.709810	-0.798353	-4.301988
O	-2.030791	0.771761	3.251650
C	-1.514242	0.363217	4.505055
H	-1.079855	-0.640343	4.451956
H	-0.730849	1.081498	4.750191
H	-2.293944	0.388910	5.276220
H	-1.596717	-3.575178	-3.412974
H	-0.640892	-3.371067	2.967550
H	1.598272	-1.942619	4.015027
H	1.488472	2.470723	1.759704
O	2.674569	2.485841	3.956020
C	3.517687	2.387022	5.090468
H	3.934858	3.384544	5.240562
H	2.956102	2.093642	5.985767
H	4.336087	1.676641	4.920833
H	-4.124611	-4.204274	0.013595
H	-6.775796	2.288914	-3.503343
C	-8.379001	2.532546	-1.324982
C	-9.067552	2.605828	-0.140985
C	-7.308535	1.395593	0.996231
C	-8.522730	2.034095	1.032697
H	-10.030013	3.107929	-0.098416
H	-8.787231	2.976976	-2.229428
H	-9.068033	2.105892	1.969642
H	-6.896942	0.967101	1.904833
H	1.267808	-0.141802	-0.655553
C	6.129275	-1.554198	-2.089048
C	7.313991	-2.165422	-1.699098
C	7.294485	-2.956812	-0.560107
C	6.130217	-3.147381	0.170064
C	4.967665	-2.518032	-0.258964
C	4.924190	-1.694848	-1.391448
H	8.226154	-2.018716	-2.265481
H	6.123151	-3.769907	1.057124
Cl	3.532334	-2.787423	0.694640
Cl	8.766816	-3.730273	-0.033164
Cl	6.186011	-0.546446	-3.516617
C	3.652425	-1.046923	-1.880936

H	2.799650	-1.387623	-1.284286
H	3.453172	-1.363822	-2.909023
N	3.944146	1.143613	-0.834082
C	3.592611	0.454335	-1.860222
C	2.993302	1.161055	-3.022776
H	3.548894	0.912053	-3.932092
H	1.972499	0.779110	-3.146582
H	2.961505	2.243358	-2.892321
O	4.289606	0.521407	0.318558
C	5.426818	1.134752	1.044313
C	5.055198	2.530391	1.531549
H	5.801657	2.854361	2.264419
H	5.047591	3.263051	0.720514
H	4.076349	2.530344	2.015332
C	5.591369	0.166083	2.205958
H	5.886658	-0.826002	1.850203
H	6.371156	0.535035	2.879446
H	4.655274	0.077062	2.767044
C	6.651920	1.158647	0.142741
H	7.486593	1.616026	0.684189
H	6.948185	0.147781	-0.150756
H	6.474557	1.758512	-0.757113
H	3.871305	2.192466	-0.829938
S	2.745039	4.680881	-1.097287
C	3.359969	6.328124	-0.819683
H	3.989320	6.621641	-1.663154
H	2.509705	7.009423	-0.734434
H	3.941301	6.340156	0.105336
O	1.964768	4.306357	0.100176
O	3.972302	3.832590	-1.257921
O	1.948068	4.729107	-2.341729

A7uR

	Coordinates (Angstroms)		
	X	Y	Z
Ir	0.373966	-0.395941	0.094269
C	1.058190	-1.842479	-1.110587
N	-0.048278	0.357003	-1.800254
C	1.544573	-3.103507	-0.742748
C	2.043778	-4.005752	-1.689812
C	2.078392	-3.670145	-3.052316

C	1.567598	-2.439977	-3.442460
C	1.051919	-1.543009	-2.501760
C	0.281552	0.190851	-4.248401
C	0.407243	-0.290956	-2.836474
C	-0.859276	1.519958	-1.921816
C	-0.470833	2.721264	-1.323878
C	-1.318486	3.816053	-1.349014
C	-2.576293	3.723191	-1.959453
C	-2.963591	2.530612	-2.570377
C	-2.098232	1.438636	-2.546990
O	-3.346112	4.843462	-1.903569
C	-4.651630	4.777856	-2.453812
H	2.472229	-4.352963	-3.796092
H	0.005528	1.245585	-4.297675
H	1.234222	0.056401	-4.770614
H	0.492734	2.787322	-0.829554
H	-2.408263	0.500845	-2.994652
H	-5.100289	5.756976	-2.277319
H	-4.621831	4.583725	-3.532900
H	-5.253192	4.006553	-1.957747
C	-0.186607	-1.569969	1.878120
C	0.615915	-0.459675	2.283996
C	-0.158561	0.736245	2.061904
C	-1.395423	0.355063	1.488924
C	-1.441121	-1.081012	1.385598
C	-2.624417	-1.943393	1.028623
C	-2.498434	1.300533	1.092646
C	-3.401338	-1.480303	-0.184106
C	-3.781918	0.949601	1.805354
C	-4.018750	1.490400	3.106172
C	-5.164989	1.192875	3.796831
C	-6.122302	0.306113	3.234631
C	-5.889164	-0.274215	1.958009
C	-4.691415	0.077246	1.251388
C	-3.096604	-2.043947	-1.464086
C	-3.805386	-1.683538	-2.582626
C	-4.817788	-0.690860	-2.495734
C	-5.112169	-0.090401	-1.242881
C	-4.394889	-0.530791	-0.082357
C	-5.515459	-0.252428	-3.652021
C	-6.457820	0.743420	-3.570876
C	-6.744885	1.348313	-2.325878
C	-6.086455	0.940950	-1.190554
H	0.082277	-2.614779	1.974166

H	1.581836	-0.519127	2.766730
H	0.139786	1.751509	2.288717
H	-2.266979	-2.962812	0.865723
H	-3.302959	-1.980768	1.889799
H	-2.197208	2.322704	1.330593
H	-2.659852	1.252324	0.014052
H	-3.594482	-2.121722	-3.552108
H	-5.286787	-0.717556	-4.608000
H	-6.983211	1.070573	-4.463925
H	-7.489677	2.137153	-2.268585
H	-6.305628	1.418212	-0.240397
O	-3.025551	2.293494	3.568065
C	-3.175028	2.875575	4.851476
H	-3.257994	2.106476	5.628795
H	-2.269794	3.461576	5.018511
H	-4.049518	3.536481	4.888806
O	-2.082319	-2.944091	-1.462380
C	-1.753303	-3.592520	-2.677872
H	-1.444597	-2.871996	-3.442953
H	-0.916894	-4.253189	-2.444818
H	-2.597761	-4.186421	-3.048437
H	-1.028557	4.753268	-0.883590
H	-0.475238	-0.388788	-4.789572
H	1.567373	-2.188707	-4.500160
H	1.526168	-3.423651	0.295986
O	2.486807	-5.186169	-1.188334
C	2.985280	-6.165531	-2.086738
H	3.259580	-7.022001	-1.468363
H	2.219303	-6.470175	-2.808755
H	3.873049	-5.803935	-2.618375
H	-3.930331	2.422931	-3.049625
H	-5.358653	1.608522	4.779944
C	-7.309932	-0.027377	3.937751
C	-8.220974	-0.905006	3.404509
C	-6.843451	-1.188685	1.438687
C	-7.982488	-1.495690	2.142810
H	-9.126181	-1.151363	3.952963
H	-7.483573	0.424827	4.911349
H	-8.702582	-2.197493	1.731285
H	-6.666773	-1.651882	0.472621
H	1.797768	0.278351	-0.184787
C	6.748213	1.515389	0.446912
C	7.533726	2.553940	-0.032939
C	6.924385	3.779592	-0.263031

C	5.571795	3.974653	-0.025993
C	4.822875	2.904888	0.451223
C	5.376883	1.643888	0.704754
H	8.591220	2.407941	-0.219464
H	5.105957	4.936401	-0.205954
Cl	3.125634	3.201970	0.742319
Cl	7.882980	5.106286	-0.863397
Cl	7.533906	-0.022434	0.725406
C	4.573763	0.490676	1.247108
H	3.595811	0.832750	1.607070
H	5.067405	0.047239	2.116986
N	3.863125	-1.727080	0.674115
C	4.277042	-0.585088	0.249305
C	4.370661	-0.384215	-1.218306
H	3.810011	0.516954	-1.488372
H	5.415886	-0.218711	-1.501272
H	3.977169	-1.239298	-1.772649
O	3.576005	-1.934267	1.980387
C	4.277273	-3.099482	2.580482
C	5.770599	-2.813707	2.605574
H	6.288134	-3.643221	3.098270
H	6.178399	-2.724853	1.592821
H	5.985619	-1.896003	3.162006
C	3.670658	-3.120984	3.973670
H	2.592109	-3.303668	3.923966
H	4.131918	-3.922303	4.558610
H	3.846469	-2.170226	4.487694
C	3.948616	-4.365200	1.801798
H	4.396944	-5.219934	2.317952
H	2.868535	-4.529511	1.749184
H	4.361175	-4.350759	0.786975
H	3.492264	-2.433784	0.028321

A7uS

	Coordinates (Angstroms)		
	X	Y	Z
Ir	0.460948	0.511122	-0.287311
C	1.175840	2.381799	-0.113939
N	-0.094350	1.027127	1.656544
C	1.795328	3.147906	-1.105303
C	2.287045	4.428275	-0.837282

C	2.177046	4.989141	0.445339
C	1.556867	4.249850	1.440962
C	1.052797	2.971634	1.175286
C	0.070677	2.630897	3.534642
C	0.321046	2.169717	2.131845
C	-0.973542	0.172914	2.378285
C	-0.624409	-1.156764	2.628240
C	-1.533940	-2.019754	3.215864
C	-2.818405	-1.571619	3.552574
C	-3.166906	-0.240192	3.326074
C	-2.239228	0.620085	2.741582
O	-3.650258	-2.506519	4.085178
C	-4.975147	-2.110789	4.402536
H	2.560991	5.978164	0.665943
H	-0.249019	1.814442	4.184726
H	0.983488	3.068302	3.950515
H	0.359472	-1.512780	2.342785
H	-2.520120	1.646437	2.532398
H	-5.471962	-3.005775	4.780610
H	-4.984724	-1.335251	5.177753
H	-5.503990	-1.746940	3.513455
C	0.018990	0.429098	-2.454140
C	0.778045	-0.721524	-2.078266
C	-0.064993	-1.533794	-1.236802
C	-1.298107	-0.861459	-1.070018
C	-1.274932	0.352800	-1.844518
C	-2.428405	1.271090	-2.151620
C	-2.455357	-1.378867	-0.259116
C	-3.287848	1.619686	-0.957748
C	-3.684441	-1.522562	-1.121359
C	-3.889426	-2.730613	-1.842218
C	-4.967972	-2.897812	-2.665313
C	-5.901364	-1.843960	-2.838134
C	-5.703990	-0.614309	-2.147287
C	-4.574436	-0.478337	-1.277884
C	-3.012336	2.822484	-0.232365
C	-3.790876	3.199846	0.833020
C	-4.847958	2.360080	1.275261
C	-5.114195	1.141611	0.595276
C	-4.321369	0.804555	-0.551182
C	-5.619257	2.694127	2.419812
C	-6.606353	1.853927	2.873937
C	-6.866846	0.637482	2.202819
C	-6.136484	0.290572	1.091576

H	0.345521	1.211257	-3.128450
H	1.761235	-0.983758	-2.445781
H	0.192847	-2.484903	-0.795914
H	-2.030434	2.186242	-2.595922
H	-3.056450	0.796376	-2.915603
H	-2.175670	-2.338769	0.178098
H	-2.669807	-0.698447	0.566562
H	-3.603314	4.122093	1.372373
H	-5.410691	3.628062	2.936505
H	-7.187479	2.120701	3.752549
H	-7.645923	-0.025449	2.568682
H	-6.336958	-0.650916	0.589773
O	-2.944680	-3.729276	-1.723760
C	-3.279051	-4.731913	-0.769703
H	-4.197779	-5.258250	-1.056981
H	-2.446088	-5.438562	-0.753646
H	-3.411915	-4.300063	0.231044
O	-1.951399	3.530232	-0.690818
C	-1.630150	4.755925	-0.057893
H	-1.414720	4.611222	1.006190
H	-0.734649	5.122719	-0.561419
H	-2.442641	5.483708	-0.174581
H	-1.275335	-3.056950	3.409010
H	-0.703170	3.407056	3.552161
H	1.459008	4.688629	2.430803
H	1.925111	2.761828	-2.113087
O	2.872127	5.060607	-1.889351
C	3.424254	6.350909	-1.684342
H	3.847772	6.650459	-2.644546
H	2.654946	7.073876	-1.388308
H	4.219161	6.329308	-0.929584
H	-4.152219	0.141682	3.570796
H	-5.098985	-3.834512	-3.200665
C	-7.019053	-1.983654	-3.703379
C	-7.902316	-0.949626	-3.881091
C	-6.632636	0.440888	-2.361568
C	-7.703424	0.276688	-3.204406
H	-8.753261	-1.066071	-4.546407
H	-7.159962	-2.927489	-4.224485
H	-8.403692	1.093455	-3.355813
H	-6.485920	1.389290	-1.853969
H	1.865615	0.137256	0.388394
C	6.050263	0.415248	-0.702968
C	7.330949	0.953247	-0.646829

C	8.387131	0.112166	-0.329610
C	8.190075	-1.236841	-0.069880
C	6.895382	-1.732573	-0.138469
C	5.786012	-0.938267	-0.457993
H	7.495257	2.006381	-0.842959
H	9.020205	-1.886501	0.181689
Cl	6.667886	-3.431074	0.211997
Cl	10.001253	0.766636	-0.247252
Cl	4.745610	1.511836	-1.080954
C	4.400696	-1.527548	-0.528831
H	4.402653	-2.479372	-1.066642
H	3.725070	-0.870633	-1.091852
N	2.814329	-2.591192	0.921235
C	3.748719	-1.713748	0.805287
C	4.073184	-0.871208	1.983145
H	5.133866	-0.976451	2.232669
H	3.893760	0.178936	1.723835
H	3.468275	-1.128966	2.855312
O	2.368274	-3.288868	-0.145453
C	2.007785	-4.697137	0.162184
C	0.904274	-4.726983	1.214638
H	0.498642	-5.741965	1.268161
H	1.268083	-4.482098	2.219214
H	0.087673	-4.049005	0.945234
C	1.501241	-5.181106	-1.187335
H	2.263235	-5.043475	-1.961155
H	1.268788	-6.248001	-1.119107
H	0.592400	-4.648395	-1.484701
C	3.261833	-5.434765	0.604574
H	3.016578	-6.484406	0.795835
H	4.029570	-5.397436	-0.174496
H	3.671774	-5.016384	1.530726
H	2.277806	-2.694586	1.784124

B1

	Coordinates (Angstroms) X	Y	Z
Ir	1.487418	-0.369189	0.404324
C	2.690633	0.132770	-1.152726
N	0.881355	1.590072	-0.002236
C	3.689141	-0.642291	-1.761327

C	4.534177	-0.085872	-2.723350
C	4.394937	1.249366	-3.113049
C	3.407867	2.050143	-2.557205
C	2.576985	1.466088	-1.585064
C	1.338631	3.681683	-1.262064
C	1.547054	2.239226	-0.907752
C	-0.127379	2.204440	0.793723
C	0.030536	2.212989	2.177277
C	-0.960204	2.741995	3.001427
C	-2.109838	3.266979	2.398823
C	-2.291887	3.258090	1.012005
C	-1.287219	2.710953	0.213802
O	-3.125872	3.768436	3.188729
C	-2.935755	5.125518	3.575174
H	5.073536	1.657548	-3.858157
H	0.955508	4.234680	-0.399757
H	0.932366	1.793264	2.613555
H	-1.417929	2.656194	-0.863028
H	-3.799415	5.401497	4.184626
H	-2.021029	5.248274	4.167405
H	-2.884118	5.784701	2.699471
C	1.226862	-2.489415	0.011333
C	1.603231	-2.339601	1.366663
C	0.520274	-1.662570	2.056816
C	-0.470097	-1.333548	1.122260
C	-0.017137	-1.786054	-0.193246
C	-0.836133	-1.902856	-1.450059
C	-1.770360	-0.634010	1.401143
C	-1.683055	-0.702882	-1.803737
C	-2.921649	-1.529713	1.009920
C	-3.402470	-2.478733	1.953813
C	-4.405868	-3.353322	1.643188
C	-4.973635	-3.350940	0.344212
C	-4.493236	-2.427195	-0.627026
C	-3.460115	-1.502878	-0.262263
C	-1.182863	0.240168	-2.756844
C	-1.956407	1.289059	-3.187760
C	-3.263923	1.472098	-2.664332
C	-3.757285	0.584331	-1.670687
C	-2.940184	-0.524540	-1.268422
C	-4.093809	2.531860	-3.117793
C	-5.355219	2.706920	-2.604131
C	-5.836350	1.842085	-1.595720
C	-5.052854	0.811889	-1.135971

H	1.799291	-2.981545	-0.760698
H	2.515342	-2.706151	1.816143
H	0.502307	-1.402237	3.107811
H	-0.155148	-2.124155	-2.274693
H	-1.488785	-2.776599	-1.333669
H	-1.815199	-0.374330	2.460433
H	-1.822602	0.296463	0.833813
H	-1.596819	1.988728	-3.934526
H	-3.712481	3.203108	-3.883573
H	-5.983039	3.517117	-2.964740
H	-6.827328	1.996852	-1.178553
H	-5.429270	0.157551	-0.356127
O	-2.801585	-2.519405	3.193210
C	-3.520948	-1.824996	4.207975
H	-4.502503	-2.284296	4.378749
H	-2.923199	-1.895855	5.119188
H	-3.660973	-0.768690	3.944351
O	0.071393	-0.013955	-3.201492
C	0.619076	0.831162	-4.197162
H	0.693237	1.866205	-3.843732
H	1.619231	0.444007	-4.395850
H	0.021658	0.795226	-5.116689
S	4.383193	0.418707	1.919639
C	5.332382	1.460485	0.834248
H	5.113401	1.189567	-0.200441
H	5.069052	2.504633	1.017417
H	6.392989	1.300097	1.044180
O	4.704951	0.820318	3.299437
O	4.713128	-0.973077	1.578997
O	2.934765	0.777597	1.620469
H	0.560940	3.725804	-2.038541
H	-4.752034	-4.065830	2.387197
C	-6.000363	-4.266057	-0.011519
C	-6.523781	-4.274825	-1.278949
C	-5.047537	-2.473732	-1.935817
C	-6.036422	-3.371045	-2.251997
H	-7.307857	-4.979264	-1.541937
H	-6.360093	-4.963665	0.740858
H	-6.447981	-3.391234	-3.257185
H	-4.678393	-1.790802	-2.694340
C	-3.560192	3.798598	0.412293
H	-4.435751	3.252401	0.778900
H	-3.709385	4.853696	0.669153
H	-3.537829	3.716852	-0.677201

C	-0.804068	2.728516	4.498389
H	-0.354931	3.660478	4.863376
H	-1.773190	2.614377	4.993693
H	-0.152895	1.906365	4.811600
C	3.202039	3.491254	-2.953819
C	2.630193	4.308190	-1.795384
H	4.146572	3.924500	-3.300484
H	2.504819	3.538111	-3.803970
H	3.368473	4.361984	-0.985401
H	2.425206	5.334207	-2.117446
H	5.319089	-0.682581	-3.177310
O	3.777466	-1.943413	-1.367693
C	4.980249	-2.658938	-1.570289
H	5.819765	-2.121477	-1.110022
H	5.189534	-2.802962	-2.638527
C	4.794966	-4.015101	-0.932586
H	5.697844	-4.620133	-1.111196
H	3.944019	-4.531024	-1.405122
O	4.565257	-3.845749	0.446520
C	4.260219	-5.056557	1.099039
H	4.126892	-4.830968	2.160067
H	3.332164	-5.501535	0.708686
H	5.071923	-5.792524	0.992433

B2

	Coordinates (Angstroms)		
	X	Y	Z
Ir	1.660562	0.063048	-0.391893
C	2.483571	1.930126	-0.479498
N	0.942426	0.950148	1.367893
C	3.368412	2.430990	-1.436789
C	3.898413	3.716251	-1.309949
C	3.593837	4.510665	-0.204535
C	2.743471	4.041879	0.789297
C	2.195006	2.759502	0.619841
C	0.946477	2.958989	2.836505
C	1.319304	2.168772	1.617836
C	0.076112	0.220281	2.236753
C	0.485139	-1.023957	2.707612
C	-0.353696	-1.786565	3.520547
C	-1.615462	-1.270207	3.834727

C	-2.052416	-0.030153	3.355851
C	-1.192345	0.709367	2.545968
O	-2.495035	-2.011867	4.597921
C	-2.299018	-1.880366	6.001746
H	4.041926	5.497156	-0.115613
H	0.720343	2.292184	3.672891
H	1.461103	-1.414403	2.434185
H	-1.522284	1.658032	2.132666
H	-3.128094	-2.403841	6.483539
H	-1.352487	-2.334652	6.318987
H	-2.308718	-0.826335	6.305770
C	1.274086	-0.212088	-2.526678
C	1.867913	-1.403752	-2.049032
C	0.942982	-2.030073	-1.126863
C	-0.180503	-1.201424	-0.999810
C	0.029150	-0.014700	-1.825719
C	-0.999438	0.993667	-2.259322
C	-1.413854	-1.500100	-0.198312
C	-1.961874	1.487918	-1.204973
C	-2.574057	-1.635499	-1.156227
C	-2.794387	-2.890450	-1.789079
C	-3.778915	-3.056321	-2.722899
C	-4.590579	-1.957536	-3.103277
C	-4.376830	-0.686551	-2.498606
C	-3.351377	-0.548952	-1.506513
C	-1.720086	2.759539	-0.597104
C	-2.625743	3.312294	0.273860
C	-3.807939	2.603930	0.615485
C	-4.038780	1.311758	0.071855
C	-3.097144	0.781168	-0.870903
C	-4.768532	3.164162	1.499092
C	-5.903481	2.469220	1.836577
C	-6.121728	1.173145	1.317365
C	-5.209196	0.607712	0.460726
H	1.695225	0.467798	-3.253466
H	2.833253	-1.793499	-2.340046
H	1.114992	-2.948609	-0.580273
H	-0.473610	1.845382	-2.695824
H	-1.573762	0.527038	-3.069268
H	-1.255758	-2.417163	0.371168
H	-1.610462	-0.697317	0.512608
H	-2.465844	4.291210	0.712704
H	-4.588299	4.156425	1.905586
H	-6.632072	2.909652	2.511622

H	-7.014087	0.621813	1.599886
H	-5.384439	-0.390202	0.071616
H	0.022320	3.509636	2.608217
H	-3.923192	-4.026135	-3.191671
C	-5.602099	-2.098118	-4.090813
C	-6.365949	-1.024151	-4.470059
C	-5.178347	0.408613	-2.923607
C	-6.147505	0.243240	-3.881175
H	-7.136312	-1.140812	-5.227168
H	-5.755733	-3.073935	-4.544987
H	-6.750199	1.091799	-4.192648
H	-5.017617	1.388480	-2.485301
C	-3.434052	0.460283	3.689094
H	-4.197128	-0.228702	3.310929
H	-3.582269	0.540080	4.772241
H	-3.612616	1.444513	3.249747
C	0.090805	-3.130864	4.031941
H	0.605448	-3.034250	4.996249
H	-0.762266	-3.800631	4.177508
H	0.789173	-3.595735	3.329604
C	2.407660	4.851827	2.017495
C	2.054696	3.951733	3.201046
H	3.249767	5.503982	2.273563
H	1.557418	5.513686	1.797237
H	2.946488	3.397462	3.519575
H	1.727192	4.556787	4.052503
H	4.575520	4.096125	-2.070226
O	3.699185	1.621101	-2.490570
C	5.058924	1.602277	-2.906637
H	5.716383	1.805013	-2.052459
H	5.240773	2.359786	-3.679813
C	5.352163	0.231559	-3.467038
H	6.376784	0.229845	-3.870498
H	4.662450	0.001824	-4.294638
O	5.217641	-0.722935	-2.436879
C	5.623025	-2.011506	-2.847919
H	5.436603	-2.690013	-2.013719
H	5.052261	-2.352512	-3.724794
H	6.694221	-2.029485	-3.100313
H	3.317144	-0.103881	0.249697
S	3.532193	-3.444202	1.282832
C	4.627133	-4.684526	1.949935
H	4.516514	-5.605903	1.372988
H	5.656666	-4.324120	1.883525

H	4.365916	-4.867801	2.995176
O	3.727155	-2.241643	2.135254
H	2.886254	-0.615265	0.772381
O	2.162986	-4.007292	1.385702
O	3.958069	-3.228780	-0.120365
O	-1.956028	-3.934437	-1.466157
C	-2.481725	-4.813069	-0.475097
H	-3.376406	-5.329299	-0.844562
H	-1.700214	-5.544000	-0.258553
H	-2.735750	-4.269771	0.444058
O	-0.565608	3.359041	-0.978500
C	-0.333475	4.693297	-0.563026
H	-1.133088	5.357384	-0.913563
H	-0.249977	4.764327	0.527037
H	0.611775	4.987450	-1.020311

B3

	Coordinates (Angstroms) X	Y	Z
Ir	-1.334588	-0.872726	-0.470158
C	-2.558042	-1.090766	1.121048
N	-1.132962	0.956245	0.500427
C	-3.375120	-2.192789	1.442780
C	-4.210143	-2.165645	2.554113
C	-4.274121	-1.029743	3.376891
C	-3.499243	0.082436	3.102976
C	-2.645049	0.021032	1.982003
C	-1.894982	2.435183	2.358643
C	-1.830784	1.149475	1.585281
C	-0.347133	1.987791	-0.088278
C	-0.607462	2.345000	-1.408180
C	0.141640	3.328964	-2.047673
C	1.175240	3.939607	-1.328514
C	1.470423	3.581476	-0.008154
C	0.700666	2.591726	0.603162
O	1.968893	4.892633	-1.937652
C	1.421616	6.205809	-1.902524
H	-4.953635	-1.028204	4.225999
H	-1.717443	3.286806	1.695112
H	-1.403111	1.845170	-1.948927
H	0.929238	2.277992	1.617375

H	2.165841	6.867756	-2.351388
H	0.489274	6.267892	-2.477238
H	1.226083	6.526338	-0.871662
C	-0.564620	-2.943015	-0.740690
C	-1.110627	-2.461746	-1.968517
C	-0.253124	-1.390645	-2.425857
C	0.754233	-1.185212	-1.453285
C	0.586699	-2.166892	-0.405870
C	1.525079	-2.463762	0.730416
C	1.843870	-0.144492	-1.524529
C	2.008602	-1.243995	1.478308
C	3.215074	-0.767155	-1.405812
C	3.869608	-1.213581	-2.586958
C	5.100020	-1.808628	-2.546109
C	5.745247	-2.022143	-1.302247
C	5.101623	-1.607534	-0.101822
C	3.825056	-0.960543	-0.180044
C	1.303159	-0.835080	2.653402
C	1.750068	0.213213	3.416820
C	2.915178	0.928250	3.030884
C	3.597064	0.579539	1.833359
C	3.119416	-0.538669	1.070323
C	3.415976	1.991347	3.827727
C	4.546427	2.677005	3.457319
C	5.212003	2.347780	2.255298
C	4.741744	1.332015	1.458523
H	-0.954838	-3.762551	-0.153191
H	-1.955179	-2.882869	-2.495735
H	-0.364815	-0.825726	-3.342559
H	1.018260	-3.137016	1.425723
H	2.391750	-3.006910	0.334864
H	1.749669	0.391623	-2.471122
H	1.711663	0.588090	-0.726000
H	1.240230	0.507387	4.328019
H	2.892095	2.247348	4.745560
H	4.926018	3.480884	4.082032
H	6.094893	2.905896	1.956748
H	5.254594	1.095088	0.531385
O	3.212748	-1.058903	-3.788841
C	3.659139	0.058736	-4.550044
H	4.717457	-0.044846	-4.819355
H	3.054403	0.080876	-5.459282
H	3.519422	0.996666	-3.997030
O	0.206649	-1.576938	2.945824

C	-0.464866	-1.325819	4.167371
H	-0.845847	-0.300262	4.214936
H	-1.303484	-2.022887	4.195558
H	0.198299	-1.512437	5.021418
H	-1.082700	2.434590	3.099211
H	5.572132	-2.137508	-3.468088
C	7.012577	-2.660517	-1.229027
C	7.614237	-2.888925	-0.018285
C	5.746296	-1.874760	1.137526
C	6.969321	-2.495644	1.177861
H	8.583052	-3.378325	0.027479
H	7.495584	-2.968682	-2.153099
H	7.446368	-2.689383	2.134524
H	5.258548	-1.587749	2.063594
C	2.605055	4.248629	0.719044
H	3.552487	4.120834	0.184667
H	2.434839	5.327276	0.818510
H	2.718740	3.831988	1.723183
C	-0.163786	3.709315	-3.472014
H	-0.904410	4.517007	-3.515299
H	0.734256	4.052194	-3.994413
H	-0.581474	2.856613	-4.016324
C	-3.535834	1.340773	3.936199
C	-3.239287	2.573354	3.080393
H	-4.510519	1.439544	4.426641
H	-2.785889	1.274830	4.739040
H	-4.035861	2.704219	2.336373
H	-3.222619	3.474214	3.702859
H	-4.837024	-3.019660	2.793020
O	-3.291362	-3.267374	0.599481
C	-4.353460	-4.201051	0.555682
H	-5.312780	-3.680846	0.431484
H	-4.395493	-4.805887	1.471553
C	-4.088168	-5.111568	-0.619590
H	-4.856004	-5.900452	-0.643685
H	-3.107548	-5.597945	-0.497974
O	-4.117520	-4.348970	-1.806268
C	-3.795544	-5.115874	-2.945388
H	-3.843358	-4.449560	-3.810391
H	-2.783015	-5.540212	-2.872256
H	-4.510478	-5.940097	-3.090782
H	-2.665718	-0.264364	-1.165520
S	-4.568190	2.671079	-1.643037
C	-5.355588	3.965235	-0.738032

H	-5.898476	4.579814	-1.460560
H	-6.049799	3.528640	-0.017391
H	-4.591705	4.560992	-0.234420
O	-3.832037	1.954626	-0.416143
H	-3.382589	1.118484	-0.707708
O	-3.576429	3.233086	-2.551191
O	-5.556762	1.759502	-2.204346

B4

	Coordinates (Angstroms) X	Y	Z
Ir	1.856116	-0.278588	0.867497
C	3.253683	0.414192	-0.415262
N	1.269566	1.694272	0.588779
C	4.350062	-0.275513	-0.973434
C	5.265271	0.370740	-1.796656
C	5.126016	1.735975	-2.093531
C	4.066589	2.461536	-1.581098
C	3.148462	1.778633	-0.755395
C	1.787961	3.915236	-0.423667
C	2.013027	2.452113	-0.167549
C	0.096858	2.182350	1.231783
C	-0.031185	2.031731	2.610556
C	-1.200202	2.411045	3.266595
C	-2.238347	2.958139	2.502486
C	-2.130809	3.122426	1.117490
C	-0.954880	2.714497	0.488431
O	-3.431099	3.296676	3.112380
C	-3.445995	4.601909	3.679256
H	5.864313	2.218713	-2.729671
H	1.283213	4.379505	0.429106
H	0.784074	1.585667	3.172233
H	-0.869189	2.785870	-0.592081
H	-4.443669	4.748398	4.099671
H	-2.701054	4.701809	4.478150
H	-3.256417	5.368537	2.917739
C	1.677924	-2.432327	0.302136
C	1.854611	-2.323902	1.712753
C	0.667918	-1.700546	2.248112
C	-0.190260	-1.388706	1.163224
C	0.418509	-1.857046	-0.056071

C	-0.200333	-1.936834	-1.424616
C	-1.543234	-0.733038	1.271220
C	-0.954717	-0.699695	-1.854722
C	-2.613869	-1.612622	0.670746
C	-3.228118	-2.600491	1.488579
C	-4.178497	-3.451063	0.995429
C	-4.552156	-3.384599	-0.370448
C	-3.933786	-2.422065	-1.217978
C	-2.963182	-1.524819	-0.664202
C	-0.274951	0.286740	-2.636911
C	-0.939232	1.383347	-3.124914
C	-2.317590	1.567276	-2.835732
C	-2.998263	0.633783	-2.007508
C	-2.281362	-0.515142	-1.532388
C	-3.034014	2.671505	-3.368194
C	-4.368207	2.843990	-3.093408
C	-5.040434	1.931639	-2.249507
C	-4.368685	0.860462	-1.711704
H	2.375757	-2.883526	-0.389079
H	2.686255	-2.719204	2.280017
H	0.458388	-1.501962	3.291262
H	0.592675	-2.145412	-2.146688
H	-0.885035	-2.793717	-1.449657
H	-1.755503	-0.534380	2.323376
H	-1.532321	0.228345	0.755156
H	-0.438276	2.118085	-3.746170
H	-2.506101	3.375358	-4.007420
H	-4.906681	3.687744	-3.515971
H	-6.091482	2.083146	-2.020588
H	-4.893226	0.171624	-1.056919
O	-2.807866	-2.705283	2.796927
C	-3.691818	-2.115135	3.744603
H	-4.662978	-2.625081	3.750953
H	-3.217665	-2.226575	4.722260
H	-3.845677	-1.049234	3.532488
O	1.035769	0.025873	-2.862944
C	1.756769	0.892859	-3.719451
H	1.766038	1.919373	-3.337540
H	2.776991	0.507302	-3.742210
H	1.335407	0.881390	-4.732378
H	1.111866	4.016537	-1.284152
H	-4.630154	-4.194909	1.646399
C	-5.514336	-4.277249	-0.914597
C	-5.843243	-4.228517	-2.245021

C	-4.288086	-2.407905	-2.595436
C	-5.217882	-3.285611	-3.094259
H	-6.578169	-4.917715	-2.651718
H	-5.981378	-5.005138	-0.255556
H	-5.472587	-3.260069	-4.150134
H	-3.809944	-1.696048	-3.260962
C	-3.272876	3.705286	0.332737
H	-4.204540	3.162090	0.519975
H	-3.450445	4.752241	0.607464
H	-3.062821	3.672499	-0.739435
C	-1.350487	2.210522	4.751152
H	-1.088783	3.119497	5.306973
H	-2.381803	1.953055	5.011209
H	-0.692631	1.409443	5.102449
C	3.854446	3.927420	-1.875632
C	3.111004	4.622695	-0.734546
H	4.817980	4.416385	-2.057842
H	3.270772	4.035355	-2.802639
H	3.741179	4.627019	0.164172
H	2.908792	5.667401	-0.992870
H	6.109857	-0.169653	-2.213370
O	4.448699	-1.598954	-0.646884
C	5.667016	-2.282573	-0.857776
H	6.495531	-1.740652	-0.381694
H	5.886643	-2.394683	-1.928346
C	5.507164	-3.654244	-0.245449
H	6.427065	-4.234614	-0.418002
H	4.675147	-4.184467	-0.734603
O	5.254584	-3.508497	1.134557
C	4.973791	-4.737759	1.766146
H	4.817266	-4.530640	2.827825
H	4.065357	-5.202831	1.354595
H	5.807692	-5.448468	1.661575
H	2.875399	0.335558	1.953408

B5fR

	Coordinates (Angstroms) X	Y	Z
Ir	0.138484	-0.681987	-0.071991
C	-0.639611	-1.218333	1.710707
N	0.380479	1.043381	1.064954

C	-1.218247	-2.455089	2.073274
C	-1.762846	-2.647394	3.337595
C	-1.749899	-1.618148	4.294539
C	-1.189011	-0.391258	3.997498
C	-0.640405	-0.222174	2.707512
C	-0.066613	2.215371	3.223694
C	-0.066330	1.035799	2.290638
C	0.987094	2.192690	0.485179
C	0.508880	2.666769	-0.734029
C	1.117126	3.744640	-1.374133
C	2.221916	4.340226	-0.755618
C	2.727863	3.874689	0.462993
C	2.105466	2.784685	1.069752
O	2.880726	5.384241	-1.375177
C	2.289970	6.658830	-1.149521
H	-2.195940	-1.796199	5.270548
H	-0.145034	3.149988	2.659163
H	-0.330718	2.165101	-1.204398
H	2.513075	2.375937	1.989789
H	2.968881	7.398598	-1.580265
H	1.310629	6.741307	-1.636928
H	2.171655	6.856166	-0.076803
C	0.950016	-2.609903	-0.849272
C	0.116113	-2.000717	-1.833751
C	0.777351	-0.789777	-2.263082
C	1.971291	-0.648428	-1.507896
C	2.103533	-1.792659	-0.641616
C	3.287885	-2.179877	0.200911
C	2.959497	0.485577	-1.628128
C	3.857693	-1.052650	1.030039
C	4.352154	-0.020896	-1.919893
C	4.746721	-0.221066	-3.271552
C	5.980896	-0.712681	-3.594630
C	6.898267	-1.060692	-2.571328
C	6.523808	-0.884569	-1.209087
C	5.231110	-0.346967	-0.903917
C	3.409234	-0.893287	2.379462
C	3.936009	0.080797	3.189236
C	4.933028	0.960057	2.688477
C	5.360181	0.850965	1.337166
C	4.800634	-0.187798	0.520147
C	5.523483	1.946006	3.522370
C	6.498113	2.784840	3.040453
C	6.908597	2.692233	1.691617

C	6.344914	1.755620	0.859142
H	0.751622	-3.542481	-0.337224
H	-0.795184	-2.419581	-2.241203
H	0.435336	-0.104226	-3.028002
H	2.985407	-2.998319	0.858265
H	4.074426	-2.572959	-0.454980
H	2.621576	1.161208	-2.415977
H	2.980069	1.061326	-0.700865
H	3.621971	0.189209	4.221962
H	5.195438	2.019614	4.556654
H	6.948483	3.528306	3.692539
H	7.665980	3.371429	1.310427
H	6.659542	1.698631	-0.178348
O	3.834269	0.065541	-4.264919
C	4.003573	1.353736	-4.848123
H	4.973058	1.432794	-5.355739
H	3.199651	1.474506	-5.577428
H	3.931918	2.145647	-4.091082
O	2.468816	-1.785312	2.772946
C	2.038676	-1.763329	4.121425
H	1.600897	-0.795551	4.387187
H	1.273856	-2.537227	4.200244
H	2.868009	-1.994302	4.801816
H	0.898921	2.245189	3.747588
H	6.248810	-0.855054	-4.638231
C	8.177442	-1.597112	-2.878392
C	9.047496	-1.952906	-1.879891
C	7.444309	-1.277078	-0.198427
C	8.672893	-1.794290	-0.524860
H	10.022743	-2.364346	-2.124730
H	8.451163	-1.725489	-3.922770
H	9.362564	-2.088819	0.261258
H	7.167030	-1.168289	0.845440
H	-1.344149	-0.126514	-0.315981
C	-6.114075	2.093150	-1.307684
C	-7.030662	3.134802	-1.239021
C	-6.580793	4.376281	-0.815241
C	-5.254481	4.587626	-0.470060
C	-4.371602	3.517370	-0.548561
C	-4.761361	2.237035	-0.967088
H	-8.068028	2.977008	-1.510140
H	-4.910215	5.562515	-0.145525
Cl	-2.710627	3.842399	-0.108922
Cl	-7.707384	5.704032	-0.717483

Cl	-6.714056	0.550118	-1.866051
C	-3.800540	1.069190	-1.038925
H	-2.769905	1.429782	-1.103980
H	-3.982698	0.469179	-1.933633
N	-4.199162	-1.023066	0.035589
C	-3.883979	0.211985	0.197535
C	-3.613901	0.793527	1.532258
H	-2.662951	1.330763	1.482607
H	-4.396926	1.522268	1.772479
H	-3.566621	0.032606	2.308500
O	-4.219092	-1.885872	1.085980
C	-5.577143	-2.358017	1.435519
C	-6.169453	-3.137288	0.267652
H	-7.107053	-3.604552	0.585825
H	-6.393914	-2.490887	-0.587124
H	-5.475688	-3.919418	-0.053657
C	-5.290637	-3.262540	2.623528
H	-4.803818	-2.703512	3.428985
H	-6.232317	-3.670029	3.004365
H	-4.645857	-4.096611	2.330819
C	-6.442000	-1.171361	1.839844
H	-7.430635	-1.534853	2.139081
H	-6.002687	-0.637202	2.688277
H	-6.589118	-0.468803	1.012484
H	-4.321914	-1.452261	-0.919363
S	-3.699063	-3.257910	-2.676229
C	-4.891926	-4.423889	-3.301733
H	-5.291311	-4.056255	-4.249919
H	-4.396177	-5.385940	-3.452651
H	-5.699303	-4.530571	-2.572791
O	-3.215676	-3.791362	-1.385377
O	-4.465728	-1.979954	-2.485114
O	-2.649642	-3.131741	-3.706770
C	3.929859	4.532698	1.081679
H	4.794839	4.491347	0.411469
H	3.737342	5.591571	1.292517
H	4.198632	4.042677	2.020898
C	0.609883	4.230422	-2.705586
H	-0.159618	5.003412	-2.585682
H	1.419277	4.661245	-3.302809
H	0.160085	3.407102	-3.269840
C	-1.138388	0.754015	4.979578
H	-0.206196	0.701512	5.562614
H	-1.959934	0.671292	5.700049

C	-1.191576	2.100854	4.257414
H	-2.162943	2.212422	3.758595
H	-1.102809	2.920823	4.977924
H	-2.234428	-3.590025	3.595885
O	-1.238339	-3.409603	1.100830
C	-1.561263	-4.743793	1.436879
H	-1.087463	-5.030629	2.385122
H	-2.647268	-4.867753	1.533507
C	-1.038486	-5.632980	0.333648
H	-1.424037	-6.654624	0.477007
H	-1.390671	-5.263720	-0.638864
O	0.374870	-5.624174	0.386828
C	0.966228	-6.259752	-0.725012
H	0.649901	-5.794066	-1.669901
H	2.049025	-6.151823	-0.619784
H	0.713858	-7.330459	-0.758024

B5fS

	Coordinates (Angstroms) X	Y	Z
Ir	0.117551	0.212892	-0.056314
C	-0.845529	-0.409185	-1.721911
N	0.562548	-1.816253	-0.097204
C	-1.598619	0.344009	-2.653048
C	-2.230626	-0.269591	-3.730754
C	-2.131808	-1.653444	-3.939579
C	-1.404954	-2.440719	-3.068721
C	-0.774863	-1.797637	-1.980543
C	0.166247	-4.032290	-1.165547
C	0.025010	-2.542192	-1.035744
C	1.390955	-2.370740	0.918211
C	1.061107	-2.153020	2.253783
C	1.890037	-2.606470	3.278474
C	3.056321	-3.296206	2.927184
C	3.410667	-3.521042	1.592322
C	2.569614	-3.038786	0.590874
O	3.926941	-3.726967	3.909354
C	3.560260	-4.965992	4.503916
H	-2.642846	-2.100792	-4.789010
H	0.294404	-4.491110	-0.179914
H	0.161994	-1.592693	2.493942

H	2.847134	-3.160391	-0.452218
H	4.345004	-5.212885	5.222519
H	2.599935	-4.890693	5.028969
H	3.492364	-5.761045	3.750689
C	0.609105	2.342149	-0.477864
C	-0.008700	2.238690	0.805409
C	0.899491	1.521233	1.662918
C	2.042327	1.169102	0.897315
C	1.881356	1.684450	-0.436003
C	2.877350	1.675904	-1.566879
C	3.246925	0.428344	1.421286
C	3.589157	0.353754	-1.759006
C	4.515459	1.200523	1.141416
C	4.945133	2.195299	2.073176
C	6.080737	2.931801	1.850709
C	6.831077	2.738337	0.659456
C	6.398630	1.784520	-0.301205
C	5.223681	1.010274	-0.023497
C	3.087875	-0.579790	-2.721668
C	3.722197	-1.776661	-2.945656
C	4.894184	-2.111584	-2.216562
C	5.396186	-1.212389	-1.238163
C	4.716335	0.033456	-1.034379
C	5.586017	-3.328717	-2.455768
C	6.730329	-3.636114	-1.761077
C	7.222494	-2.749158	-0.777170
C	6.564040	-1.572300	-0.515163
H	0.197047	2.835719	-1.347560
H	-0.950250	2.682580	1.098586
H	0.754446	1.297674	2.711953
H	2.346820	1.942737	-2.484287
H	3.625503	2.459469	-1.393683
H	3.126601	0.266330	2.494762
H	3.315765	-0.555613	0.952655
H	3.359075	-2.479139	-3.688134
H	5.197953	-4.012075	-3.207617
H	7.255185	-4.566739	-1.959459
H	8.120074	-3.004225	-0.220702
H	6.943478	-0.901893	0.249815
O	4.135369	2.333016	3.154805
C	4.431603	3.353280	4.091928
H	4.427745	4.340534	3.615085
H	3.639496	3.309281	4.841296
H	5.400156	3.183082	4.577590

O	1.983144	-0.170068	-3.389811
C	1.494600	-0.980416	-4.442603
H	1.192362	-1.969117	-4.080708
H	0.621009	-0.459892	-4.836739
H	2.246836	-1.088375	-5.234143
H	1.083590	-4.243434	-1.733058
H	6.420537	3.678882	2.560019
C	8.001037	3.499644	0.396838
C	8.701926	3.333379	-0.771997
C	7.134924	1.647711	-1.507641
C	8.259890	2.401230	-1.737787
H	9.595472	3.922444	-0.960339
H	8.330737	4.223266	1.138650
H	8.810198	2.283526	-2.667122
H	6.799672	0.938642	-2.258173
H	-1.220809	-0.308020	0.614438
C	-6.184048	-1.653303	-1.065748
C	-7.454871	-2.215655	-1.098244
C	-8.442753	-1.660972	-0.298432
C	-8.186543	-0.568286	0.516409
C	-6.903114	-0.037136	0.517478
C	-5.858028	-0.550427	-0.263644
H	-7.665445	-3.066170	-1.736217
H	-8.963196	-0.135864	1.136514
Cl	-6.622637	1.345351	1.548030
Cl	-10.044681	-2.351256	-0.322193
Cl	-4.977381	-2.376822	-2.100107
C	-4.474596	0.062710	-0.227083
H	-4.530247	1.155238	-0.245470
H	-3.904050	-0.227952	-1.115582
N	-3.291939	0.471835	1.801828
C	-3.678224	-0.415838	0.955185
C	-3.344642	-1.853608	1.100279
H	-4.271256	-2.425234	1.230273
H	-2.871399	-2.190731	0.171650
H	-2.676767	-2.041537	1.938394
O	-2.414797	0.148507	2.789788
C	-2.954565	0.284997	4.157830
C	-4.125732	-0.670572	4.336260
H	-4.498948	-0.597574	5.362999
H	-4.955791	-0.420724	3.666295
H	-3.821860	-1.706321	4.153370
C	-1.750479	-0.131243	4.989500
H	-0.903666	0.535836	4.798263

H	-2.001022	-0.076843	6.053317
H	-1.455905	-1.159818	4.757148
C	-3.342639	1.733738	4.416281
H	-3.562496	1.863937	5.480889
H	-2.522372	2.405829	4.144631
H	-4.235237	2.025062	3.854388
H	-3.436089	1.504381	1.657130
S	-3.549508	3.994503	0.329907
C	-4.731614	5.286138	0.658605
H	-4.424528	5.829706	1.555311
H	-4.756556	5.964044	-0.198313
H	-5.716615	4.838152	0.808607
O	-4.047617	3.260134	-0.853860
O	-3.556105	3.148746	1.566490
O	-2.246945	4.660674	0.125918
C	4.688994	-4.240912	1.263518
H	5.557294	-3.709618	1.667406
H	4.701000	-5.249272	1.693921
H	4.816272	-4.332602	0.181774
C	1.546486	-2.327437	4.717842
H	0.851926	-3.076729	5.117807
H	2.440684	-2.332355	5.347803
H	1.065992	-1.348767	4.814230
C	-1.251702	-3.932781	-3.247101
H	-0.392553	-4.141409	-3.902780
H	-2.133747	-4.342169	-3.752381
C	-1.031703	-4.633087	-1.906171
H	-1.933392	-4.533851	-1.288101
H	-0.861356	-5.703681	-2.060151
H	-2.829264	0.315987	-4.421256
O	-1.690292	1.689027	-2.425129
C	-2.089424	2.529313	-3.493958
H	-1.618355	2.200045	-4.429786
H	-3.179620	2.507031	-3.621708
C	-1.646345	3.935522	-3.175258
H	-2.065149	4.622841	-3.927604
H	-2.019243	4.230612	-2.187893
O	-0.231318	3.979243	-3.200744
C	0.278511	5.182183	-2.667227
H	-0.067398	5.340960	-1.636226
H	1.369053	5.101615	-2.671254
H	-0.019043	6.050060	-3.275728

B5uR

	Coordinates (Angstroms) X	Y	Z
Ir	-0.136247	-1.301353	-0.259717
C	0.825275	-1.231330	-2.020190
N	0.079600	0.738532	-0.562527
C	1.343896	-2.305430	-2.766905
C	2.021832	-2.090212	-3.962035
C	2.242905	-0.789777	-4.440075
C	1.789068	0.305902	-3.725431
C	1.082988	0.059007	-2.531079
C	0.923625	2.563534	-2.039989
C	0.645824	1.134988	-1.668367
C	-0.350686	1.654992	0.437070
C	0.155300	1.518300	1.727499
C	-0.265648	2.356096	2.756945
C	-1.224164	3.332408	2.459290
C	-1.756497	3.479853	1.173728
C	-1.311856	2.624722	0.165018
O	-1.713361	4.143272	3.463963
C	-0.915857	5.292485	3.726940
H	2.790111	-0.648257	-5.368935
H	1.056968	3.174802	-1.142085
H	0.871822	0.729409	1.934453
H	-1.735594	2.698171	-0.832668
H	-1.460836	5.890729	4.460431
H	0.061517	5.017147	4.141140
H	-0.762865	5.884145	2.816212
C	-1.377312	-3.129036	-0.480195
C	-0.632277	-3.195393	0.735946
C	-1.145241	-2.170087	1.611351
C	-2.153271	-1.459727	0.909757
C	-2.331427	-2.066472	-0.386237
C	-3.404239	-1.777093	-1.400916
C	-2.938350	-0.295775	1.458350
C	-3.652587	-0.307762	-1.655353
C	-4.423356	-0.566665	1.408877
C	-5.046219	-1.225084	2.504912
C	-6.382071	-1.517138	2.502082
C	-7.178448	-1.194269	1.374365
C	-6.573628	-0.562604	0.251134
C	-5.178245	-0.241100	0.298044

C	-2.988440	0.330259	-2.750896
C	-3.237970	1.643941	-3.060062
C	-4.146910	2.401855	-2.274262
C	-4.778897	1.803785	-1.150935
C	-4.517774	0.421346	-0.869212
C	-4.440223	3.753953	-2.593844
C	-5.324097	4.481133	-1.834969
C	-5.937416	3.894395	-0.705245
C	-5.663016	2.591550	-0.367214
H	-1.259951	-3.788549	-1.329533
H	0.126080	-3.926535	0.975263
H	-0.828056	-1.968322	2.626552
H	-3.129775	-2.267497	-2.337695
H	-4.339853	-2.239354	-1.062609
H	-2.615090	-0.108715	2.484396
H	-2.718089	0.605691	0.883368
H	-2.766202	2.125546	-3.909815
H	-3.957509	4.202541	-3.458951
H	-5.545733	5.512797	-2.094240
H	-6.620075	4.480799	-0.096813
H	-6.131055	2.154282	0.509097
O	-4.256110	-1.600808	3.570930
C	-4.297574	-0.698197	4.671350
H	-5.303726	-0.649615	5.105915
H	-3.596979	-1.082166	5.416211
H	-3.989773	0.311542	4.369603
O	-2.141077	-0.468800	-3.445111
C	-1.537129	0.048895	-4.617072
H	-0.929261	0.933479	-4.398715
H	-0.892460	-0.745521	-4.995597
H	-2.295521	0.300387	-5.368478
H	0.041163	2.959640	-2.561996
H	-6.828720	-2.015762	3.358169
C	-8.564131	-1.506207	1.335644
C	-9.317868	-1.214522	0.227594
C	-7.380441	-0.288484	-0.887417
C	-8.715957	-0.604133	-0.897762
H	-10.377123	-1.455567	0.207895
H	-9.015787	-1.983695	2.201847
H	-9.315653	-0.386754	-1.777291
H	-6.929361	0.172012	-1.760758
C	-2.808409	4.520167	0.906377
H	-3.704803	4.338416	1.509157
H	-2.451009	5.525978	1.155894

H	-3.096997	4.515264	-0.147754
C	0.284574	2.197652	4.148867
H	1.092596	2.913991	4.343725
H	-0.489876	2.365768	4.903959
H	0.695205	1.192684	4.291279
C	2.024631	1.731083	-4.161609
C	2.146948	2.665679	-2.956961
H	2.923619	1.789776	-4.784633
H	1.185532	2.066264	-4.790068
H	3.049382	2.410543	-2.386250
H	2.260771	3.702119	-3.291076
H	2.399475	-2.936140	-4.530823
O	1.151721	-3.564261	-2.254989
C	2.192708	-4.516883	-2.398432
H	3.165697	-4.010972	-2.442388
H	2.063274	-5.104609	-3.316633
C	2.147019	-5.436298	-1.199746
H	2.898741	-6.230851	-1.330333
H	1.158251	-5.914578	-1.124503
O	2.412897	-4.677855	-0.040016
C	2.384436	-5.448361	1.139875
H	2.511825	-4.757662	1.976196
H	1.425407	-5.975312	1.255357
H	3.193863	-6.193914	1.154503
H	1.374190	-1.261807	0.259403
C	5.307885	2.098791	0.984278
C	6.503061	2.803468	0.935725
C	7.271898	2.711994	-0.215383
C	6.866583	1.945701	-1.297750
C	5.666249	1.251317	-1.205959
C	4.845853	1.296823	-0.068736
H	6.819622	3.411659	1.774974
H	7.467440	1.888344	-2.197930
Cl	5.194935	0.313434	-2.600680
Cl	8.776481	3.586168	-0.306446
Cl	4.356038	2.282795	2.441893
C	3.531535	0.546743	0.019874
H	3.122076	0.384095	-0.979968
H	2.794833	1.131457	0.575460
N	3.396113	-0.948392	1.888056
C	3.710758	-0.811856	0.645304
C	4.247734	-1.943067	-0.141352
H	3.691766	-1.995315	-1.083206
H	5.297362	-1.734100	-0.382365

H	4.171338	-2.890439	0.387201
O	3.436603	-2.149922	2.511194
C	4.427014	-2.204339	3.620052
C	5.811655	-1.898073	3.069711
H	6.085304	-2.608304	2.283136
H	5.878769	-0.879561	2.671167
H	6.545165	-1.981184	3.878039
C	4.301580	-3.650596	4.069907
H	4.997289	-3.831956	4.894887
H	3.286542	-3.863984	4.420135
H	4.550123	-4.335191	3.252604
C	4.001169	-1.235252	4.714478
H	4.137192	-0.189206	4.419360
H	2.952974	-1.397487	4.986599
H	4.616682	-1.401643	5.603824
H	2.958510	-0.199086	2.424881

B5uS

	Coordinates (Angstroms) X	Y	Z
Ir	0.277797	0.218315	-0.335593
C	1.277298	0.182100	1.442077
N	-0.057053	-1.720233	0.329640
C	1.975765	1.189094	2.134906
C	2.723188	0.920109	3.278597
C	2.756083	-0.356767	3.838534
C	2.040988	-1.385022	3.245786
C	1.325986	-1.092938	2.068875
C	0.472329	-3.506478	1.975617
C	0.544656	-2.117304	1.410571
C	-0.939496	-2.570846	-0.395035
C	-0.687025	-2.837416	-1.737128
C	-1.587766	-3.581350	-2.497549
C	-2.748239	-4.052609	-1.871232
C	-3.018302	-3.800707	-0.521698
C	-2.101651	-3.046571	0.208635
O	-3.695844	-4.737258	-2.605621
C	-3.433019	-6.128630	-2.746074
H	3.343806	-0.538826	4.734591
H	0.305989	-4.235287	1.176577
H	0.211151	-2.437431	-2.199105

H	-2.310720	-2.795137	1.245155
H	-4.286377	-6.555211	-3.278021
H	-2.518921	-6.309011	-3.324815
H	-3.335447	-6.614427	-1.767172
C	-0.283992	2.345603	-0.654642
C	0.207878	1.803940	-1.878638
C	-0.719588	0.802355	-2.319763
C	-1.770127	0.731376	-1.361976
C	-1.527843	1.699139	-0.330430
C	-2.454125	2.100786	0.787380
C	-2.969584	-0.170441	-1.485246
C	-3.128693	0.940889	1.485154
C	-4.244939	0.635331	-1.460665
C	-4.765141	1.162763	-2.674016
C	-5.899390	1.927009	-2.693562
C	-6.572783	2.233643	-1.483483
C	-6.057218	1.737338	-0.252475
C	-4.879472	0.924427	-0.269066
C	-2.536464	0.396466	2.668855
C	-3.110618	-0.665741	3.322047
C	-4.318118	-1.235917	2.837251
C	-4.923315	-0.712228	1.663702
C	-4.296199	0.393641	1.000616
C	-4.942862	-2.318592	3.511795
C	-6.120392	-2.851596	3.047262
C	-6.718290	-2.337495	1.874648
C	-6.128003	-1.299948	1.195277
H	0.179935	3.154940	-0.108092
H	1.095178	2.131413	-2.401811
H	-0.664536	0.222230	-3.231911
H	-1.882781	2.681205	1.513728
H	-3.222855	2.771726	0.383717
H	-2.886004	-0.735350	-2.415537
H	-2.983097	-0.895301	-0.670675
H	-2.671218	-1.079154	4.223114
H	-4.475074	-2.714588	4.410271
H	-6.591828	-3.675315	3.576363
H	-7.642540	-2.772160	1.504615
H	-6.588926	-0.920265	0.288855
O	-4.069980	0.916097	-3.838938
C	-4.578306	-0.175709	-4.599022
H	-5.595665	0.030412	-4.953748
H	-3.910780	-0.296492	-5.454853
H	-4.585354	-1.102372	-4.009940

O	-1.406487	1.022884	3.079813
C	-0.820466	0.618373	4.304342
H	-0.479635	-0.421882	4.261221
H	0.038462	1.273597	4.452423
H	-1.525145	0.743205	5.135868
H	-0.398146	-3.561639	2.644468
H	-6.274644	2.316053	-3.636460
C	-7.743348	3.038892	-1.472453
C	-8.373166	3.344731	-0.293017
C	-6.727022	2.083273	0.953061
C	-7.855592	2.863747	0.932782
H	-9.269103	3.959472	-0.295434
H	-8.130657	3.410478	-2.418067
H	-8.356025	3.116038	1.863550
H	-6.335647	1.724636	1.900169
C	-4.294154	-4.297043	0.099633
H	-5.169130	-3.888184	-0.416947
H	-4.369588	-5.389333	0.043324
H	-4.351905	-4.007149	1.152326
C	-1.329372	-3.844674	-3.956977
H	-0.815252	-4.802509	-4.104332
H	-2.265339	-3.883829	-4.522535
H	-0.695184	-3.062069	-4.385418
C	1.998372	-2.774041	3.837149
C	1.738036	-3.835572	2.770830
H	2.935523	-2.980780	4.365995
H	1.198582	-2.818998	4.591413
H	2.595658	-3.890751	2.089077
H	1.627617	-4.820642	3.235453
H	3.305889	1.712384	3.740565
O	2.005284	2.468978	1.597542
C	1.921610	3.583652	2.491143
H	2.844506	4.165212	2.402736
H	1.823169	3.243111	3.525197
C	0.713550	4.425802	2.162617
H	0.640966	5.239815	2.900760
H	-0.194336	3.809525	2.245830
O	0.841678	4.948755	0.858971
C	-0.276503	5.718022	0.468999
H	-0.100777	6.048755	-0.557291
H	-1.202309	5.124760	0.500038
H	-0.399407	6.597691	1.116835
H	1.652644	-0.391047	-0.886181
C	5.445784	-2.134622	-0.458988

C	6.536014	-2.969038	-0.676102
C	7.809929	-2.431850	-0.561563
C	8.012329	-1.096034	-0.243318
C	6.894890	-0.300409	-0.031995
C	5.582762	-0.781684	-0.123805
H	6.389883	-4.012879	-0.928098
H	9.011426	-0.684566	-0.159299
Cl	7.166782	1.382028	0.361838
Cl	9.193110	-3.459663	-0.826182
Cl	3.858352	-2.849534	-0.626190
C	4.401933	0.113943	0.143183
H	4.546748	0.672606	1.071982
H	3.497908	-0.487586	0.289346
N	3.553437	2.183586	-0.665074
C	4.078090	1.052730	-0.985889
C	4.279015	0.652290	-2.395581
H	5.347274	0.496971	-2.584942
H	3.779381	-0.311938	-2.547207
H	3.879474	1.384432	-3.094593
O	3.065282	3.044804	-1.593203
C	3.835308	4.307841	-1.708341
C	4.045398	4.923766	-0.332454
H	3.088082	5.065036	0.177422
H	4.709747	4.314347	0.291318
H	4.520355	5.902557	-0.453420
C	2.896457	5.145098	-2.561608
H	3.346925	6.124210	-2.750048
H	2.709079	4.659700	-3.525314
H	1.941588	5.290274	-2.045815
C	5.151705	4.015835	-2.413607
H	5.784884	3.346137	-1.821257
H	4.981989	3.566744	-3.397118
H	5.702162	4.952203	-2.552766
H	3.245045	2.405916	0.299587

B6

	Coordinates (Angstroms) X	Y	Z
Ir	1.791470	-0.263518	0.874063
C	3.229011	0.352886	-0.437862
N	1.264113	1.740690	0.588985

C	4.285981	-0.374440	-0.991212
C	5.249777	0.259706	-1.789836
C	5.172947	1.623256	-2.047231
C	4.133552	2.392768	-1.523919
C	3.186240	1.731329	-0.735094
C	1.894205	3.918414	-0.419296
C	2.060309	2.456707	-0.147574
C	0.082911	2.268615	1.186684
C	-0.073929	2.169077	2.565298
C	-1.259704	2.571284	3.177181
C	-2.279102	3.083467	2.364667
C	-2.138150	3.196139	0.976658
C	-0.945592	2.770204	0.393337
O	-3.488060	3.434574	2.928129
C	-3.529872	4.758425	3.451085
H	5.935711	2.092265	-2.663600
H	1.356003	4.400658	0.401682
H	0.728419	1.749887	3.167364
H	-0.828370	2.801625	-0.686380
H	-4.540400	4.908289	3.837388
H	-2.808851	4.890382	4.267043
H	-3.324611	5.499978	2.669448
C	1.707732	-2.403775	0.524266
C	1.830030	-2.201256	1.913069
C	0.624588	-1.546757	2.391004
C	-0.203071	-1.277174	1.295545
C	0.494587	-1.740701	0.090568
C	-0.065260	-1.864138	-1.298357
C	-1.561399	-0.637911	1.324264
C	-0.859840	-0.679697	-1.798423
C	-2.574679	-1.576179	0.712767
C	-3.168250	-2.570301	1.536833
C	-4.060906	-3.475452	1.033303
C	-4.388895	-3.458637	-0.345711
C	-3.783497	-2.492079	-1.198386
C	-2.872694	-1.539994	-0.635498
C	-0.206503	0.283583	-2.630685
C	-0.900653	1.334776	-3.174138
C	-2.285031	1.491301	-2.901536
C	-2.945611	0.571760	-2.042713
C	-2.199680	-0.532084	-1.511643
C	-3.028602	2.551597	-3.484256
C	-4.368980	2.695199	-3.225064
C	-5.021983	1.797093	-2.350825

C	-4.325331	0.766919	-1.767661
H	2.416812	-2.894528	-0.123884
H	2.662640	-2.514497	2.529540
H	0.420180	-1.272097	3.417992
H	0.764366	-2.064189	-1.979628
H	-0.701597	-2.757932	-1.301272
H	-1.819040	-0.405057	2.358735
H	-1.546089	0.301421	0.768931
H	-0.419628	2.050879	-3.831563
H	-2.516394	3.244442	-4.147639
H	-4.928730	3.505236	-3.684240
H	-6.078790	1.927130	-2.136176
H	-4.834877	0.087643	-1.091404
O	-2.780732	-2.623503	2.857248
C	-3.729921	-2.080713	3.770498
H	-4.673685	-2.638474	3.740247
H	-3.288420	-2.170086	4.765377
H	-3.928659	-1.023823	3.551074
O	1.110313	0.048554	-2.846914
C	1.813778	0.908535	-3.725350
H	1.807696	1.942970	-3.363885
H	2.839516	0.537852	-3.745604
H	1.389283	0.868978	-4.735869
H	1.264388	4.018076	-1.315513
H	-4.499310	-4.225022	1.686636
C	-5.292669	-4.405894	-0.897698
C	-5.576757	-4.404791	-2.239107
C	-4.089521	-2.527874	-2.587009
C	-4.963258	-3.457595	-3.092318
H	-6.268915	-5.133668	-2.651405
H	-5.751134	-5.136020	-0.235344
H	-5.183344	-3.470040	-4.156101
H	-3.621228	-1.812111	-3.255311
C	-3.265609	3.736634	0.142747
H	-4.192936	3.183349	0.321594
H	-3.466614	4.787959	0.381621
H	-3.025657	3.673929	-0.921635
C	-1.447720	2.427666	4.663564
H	-1.178568	3.350631	5.191585
H	-2.489713	2.200619	4.908449
H	-0.814774	1.626789	5.058468
C	4.011006	3.872857	-1.790917
C	3.250949	4.582636	-0.671891
H	5.006532	4.312494	-1.914858

H	3.482331	4.025676	-2.743514
H	3.844126	4.558531	0.250921
H	3.091019	5.634417	-0.927547
H	6.068102	-0.312047	-2.216366
O	4.327396	-1.708922	-0.732026
C	5.566755	-2.396895	-0.777898
H	6.335693	-1.826311	-0.241014
H	5.897931	-2.554138	-1.812019
C	5.343212	-3.736587	-0.118329
H	6.271744	-4.324957	-0.177386
H	4.555744	-4.289114	-0.654314
O	4.967962	-3.521429	1.225547
C	4.646466	-4.721464	1.894307
H	4.418195	-4.467351	2.932552
H	3.770453	-5.211593	1.443865
H	5.488206	-5.429874	1.880094

B7fR

	Coordinates (Angstroms) X	Y	Z
Ir	-0.139049	1.009724	0.397480
C	0.296009	1.428748	2.326578
N	-0.063331	-0.883270	1.259919
C	0.499959	2.690474	2.929089
C	0.839128	2.798304	4.273631
C	1.008063	1.653478	5.068944
C	0.829146	0.393603	4.532661
C	0.463884	0.310108	3.169990
C	0.399349	-2.234953	3.313781
C	0.243021	-0.964740	2.525904
C	-0.307818	-2.029279	0.453924
C	0.340577	-2.123164	-0.775632
C	0.104245	-3.187215	-1.638987
C	-0.816426	-4.163976	-1.241117
C	-1.496959	-4.084644	-0.022161
C	-1.239038	-2.999633	0.817272
O	-1.116323	-5.204485	-2.098818
C	-0.246874	-6.324755	-1.981471
H	1.289087	1.770326	6.113030
H	0.689148	-3.065255	2.663502
H	1.005373	-1.320885	-1.076358

H	-1.793621	-2.898217	1.745025
H	-0.599922	-7.069331	-2.698628
H	0.790025	-6.057976	-2.220002
H	-0.280472	-6.750523	-0.971112
C	-1.270638	2.796782	-0.317855
C	-0.249101	2.466438	-1.258980
C	-0.592752	1.195499	-1.841591
C	-1.794169	0.745555	-1.230177
C	-2.244464	1.745877	-0.299366
C	-3.547218	1.780766	0.456823
C	-2.485609	-0.554493	-1.553641
C	-3.885127	0.474767	1.139807
C	-3.932587	-0.346985	-1.925055
C	-4.283150	-0.108696	-3.282288
C	-5.580768	0.100435	-3.660218
C	-6.613175	0.107680	-2.687595
C	-6.284608	-0.106403	-1.318819
C	-4.918860	-0.341049	-0.959096
C	-3.504530	0.286692	2.506934
C	-3.764024	-0.893028	3.157986
C	-4.432593	-1.948607	2.482517
C	-4.821843	-1.784685	1.125993
C	-4.535987	-0.539807	0.472983
C	-4.731808	-3.169201	3.143707
C	-5.396646	-4.178809	2.491876
C	-5.773108	-4.021954	1.138870
C	-5.482485	-2.857275	0.470819
H	-1.315883	3.706543	0.267119
H	0.579103	3.106389	-1.533058
H	-0.044413	0.658243	-2.603471
H	-3.491891	2.580626	1.198221
H	-4.355359	2.045023	-0.236049
H	-1.945891	-1.039391	-2.368823
H	-2.437646	-1.227257	-0.695481
H	-3.489197	-1.037161	4.197421
H	-4.434006	-3.287914	4.182929
H	-5.626146	-5.104742	3.012269
H	-6.283380	-4.830945	0.623647
H	-5.763031	-2.749647	-0.572470
O	-3.269121	-0.056204	-4.216132
C	-3.045452	-1.285266	-4.899717
H	-3.903290	-1.547495	-5.531768
H	-2.163129	-1.135750	-5.526061
H	-2.856596	-2.105396	-4.194984

O	-2.912834	1.359296	3.085464
C	-2.633741	1.307152	4.473000
H	-1.917768	0.512894	4.708799
H	-2.194988	2.273647	4.723031
H	-3.555176	1.160835	5.050320
H	-0.580084	-2.497764	3.738505
H	-5.816961	0.280867	-4.705600
C	-7.967518	0.339629	-3.049141
C	-8.953669	0.364387	-2.096106
C	-7.328850	-0.061443	-0.355072
C	-8.628407	0.164738	-0.733824
H	-9.986573	0.542811	-2.382042
H	-8.206259	0.500715	-4.097664
H	-9.413076	0.194277	0.017018
H	-7.091374	-0.205865	0.694317
H	1.455326	0.895554	0.369351
C	6.429060	-1.825875	-0.851256
C	7.487484	-2.382447	-0.146565
C	7.191990	-3.231194	0.911179
C	5.882941	-3.529531	1.258997
C	4.854858	-2.948485	0.525450
C	5.084585	-2.076932	-0.546244
H	8.512883	-2.156535	-0.414973
H	5.661258	-4.197118	2.083439
Cl	3.221838	-3.360297	0.992034
Cl	8.498444	-3.939517	1.824014
Cl	6.822440	-0.749669	-2.171991
C	3.971073	-1.439031	-1.336505
H	3.022472	-1.946174	-1.131475
H	4.138627	-1.553608	-2.411314
N	3.131618	0.755211	-1.867611
C	3.764350	0.015987	-1.027792
C	4.194436	0.628532	0.256539
H	3.712331	0.078668	1.074586
H	5.276733	0.527615	0.384464
H	3.913769	1.680556	0.324487
O	2.559857	0.202120	-2.966147
C	2.805420	0.936761	-4.233182
C	4.299750	1.170819	-4.395997
H	4.485707	1.616668	-5.378281
H	4.686981	1.862157	-3.639994
H	4.850270	0.226508	-4.338320
C	2.267290	-0.049884	-5.258996
H	1.205066	-0.252240	-5.085164

H	2.372361	0.378837	-6.260297
H	2.821174	-0.993846	-5.227003
C	2.010069	2.236107	-4.241372
H	2.143782	2.722565	-5.213664
H	0.942359	2.044391	-4.103330
H	2.351659	2.937605	-3.474857
H	3.015991	1.783355	-1.708746
S	3.105929	4.546779	-0.807825
C	4.339299	5.816676	-1.021311
H	4.481091	5.999096	-2.089081
H	3.992816	6.728549	-0.528741
H	5.276350	5.482197	-0.570033
O	2.979644	4.314676	0.645196
O	3.664461	3.358123	-1.528116
O	1.872091	5.052819	-1.449338
C	-2.499368	-5.140511	0.354185
H	-3.273017	-5.246750	-0.413511
H	-2.021256	-6.120955	0.466898
H	-2.985358	-4.894583	1.301688
C	0.799113	-3.262959	-2.971682
H	1.628257	-3.980968	-2.953715
H	0.109992	-3.584184	-3.759599
H	1.211485	-2.287431	-3.245920
C	0.995263	-0.870091	5.342965
H	0.047309	-1.113883	5.846356
H	1.736167	-0.712772	6.134945
C	1.401045	-2.046686	4.456452
H	2.400810	-1.867267	4.040617
H	1.453668	-2.968605	5.045379
H	0.995017	3.771175	4.728974
O	0.361845	3.770136	2.109852
C	0.402066	5.070573	2.659929
H	-0.346403	5.172856	3.458391
H	1.394420	5.291738	3.074980
C	0.105685	6.036649	1.539125
H	0.252219	7.066828	1.899855
H	0.801420	5.855295	0.710265
O	-1.229660	5.848531	1.110711
C	-1.486397	6.472345	-0.129468
H	-0.823730	6.083837	-0.916006
H	-2.525266	6.255180	-0.392226
H	-1.353801	7.563096	-0.065026

B7fS

	Coordinates (Angstroms)		
	X	Y	Z
Ir	0.071417	-0.673201	-0.376607
C	-0.878787	-1.138069	1.351533
N	0.000677	1.158767	0.590786
C	-1.266990	-2.397192	1.859094
C	-1.957646	-2.506191	3.061853
C	-2.297721	-1.372090	3.811913
C	-1.912488	-0.114749	3.386411
C	-1.190857	-0.031090	2.176668
C	-0.856511	2.472984	2.534445
C	-0.651367	1.229127	1.717293
C	0.688184	2.273949	0.036442
C	0.458637	2.619657	-1.292899
C	1.168443	3.653467	-1.899260
C	2.126631	4.330877	-1.137013
C	2.394316	3.985654	0.192552
C	1.668804	2.943044	0.767882
O	2.880402	5.332707	-1.716832
C	2.254452	6.610589	-1.689279
H	-2.853486	-1.492145	4.739065
H	-0.820498	3.364594	1.902305
H	-0.258252	2.047445	-1.872925
H	1.896551	2.623742	1.780908
H	2.969000	7.319735	-2.113721
H	1.335097	6.620626	-2.288205
H	2.012694	6.910255	-0.661947
C	1.088244	-2.559643	-0.960155
C	0.455494	-1.998103	-2.106530
C	1.130058	-0.767731	-2.416351
C	2.152792	-0.574004	-1.446362
C	2.153651	-1.691595	-0.544241
C	3.152886	-2.016736	0.536089
C	3.117541	0.583279	-1.436576
C	3.579643	-0.832062	1.375498
C	4.539064	0.083229	-1.506613
C	5.137905	-0.150238	-2.774764
C	6.407185	-0.647043	-2.886707
C	7.150104	-0.970351	-1.722519
C	6.565167	-0.763686	-0.441070
C	5.243837	-0.219428	-0.358374

C	2.924033	-0.583590	2.623740
C	3.297087	0.468946	3.422585
C	4.346731	1.335735	3.019347
C	4.998000	1.122709	1.775037
C	4.592877	0.007930	0.968152
C	4.767330	2.411894	3.845155
C	5.790851	3.240100	3.455005
C	6.426033	3.042276	2.208336
C	6.032507	2.014311	1.385882
H	0.840433	-3.494241	-0.482508
H	-0.359029	-2.437061	-2.666760
H	0.924088	-0.107571	-3.248983
H	2.718395	-2.786751	1.178248
H	4.043711	-2.457244	0.070388
H	2.891975	1.235120	-2.281972
H	2.986442	1.174783	-0.529476
H	2.817460	0.652156	4.377941
H	4.268000	2.563976	4.799205
H	6.108666	4.054686	4.100221
H	7.223390	3.711273	1.897107
H	6.521191	1.878186	0.426155
O	4.389344	0.108109	-3.903942
C	4.641632	1.385205	-4.481342
H	5.667758	1.449769	-4.864602
H	3.936702	1.499906	-5.307612
H	4.480694	2.189931	-3.752006
O	1.952975	-1.470878	2.944871
C	1.339536	-1.365270	4.216360
H	0.812658	-0.411251	4.328811
H	0.620129	-2.182553	4.266782
H	2.079310	-1.476474	5.019020
H	-0.025539	2.558082	3.249163
H	6.840300	-0.812413	-3.869625
C	8.460697	-1.511718	-1.809469
C	9.159276	-1.842821	-0.676465
C	7.312328	-1.126640	0.712878
C	8.575262	-1.651423	0.597780
H	10.159736	-2.259385	-0.754166
H	8.899571	-1.661575	-2.792964
H	9.130811	-1.924796	1.490619
H	6.870053	-0.992048	1.695222
H	-1.434610	-0.357180	-0.828693
C	-4.312163	3.339924	-0.446827
C	-5.021776	4.491127	-0.770485

C	-6.403724	4.467099	-0.664157
C	-7.077614	3.329127	-0.245501
C	-6.328085	2.203336	0.067619
C	-4.929654	2.158887	-0.014716
H	-4.501713	5.383179	-1.099857
H	-8.158093	3.315758	-0.162656
Cl	-7.206456	0.785765	0.587125
Cl	-7.317458	5.897060	-1.067691
Cl	-2.576776	3.421568	-0.607743
C	-4.165755	0.912355	0.369944
H	-4.539712	0.507660	1.311612
H	-3.106552	1.146461	0.529874
N	-4.335714	-1.386252	-0.372336
C	-4.170486	-0.151543	-0.686863
C	-3.935177	0.192978	-2.115828
H	-4.827149	0.694585	-2.509955
H	-3.095735	0.890445	-2.188159
H	-3.719985	-0.688103	-2.723005
O	-4.519394	-1.741930	0.922906
C	-5.327866	-2.967119	1.121754
C	-4.475584	-4.182117	0.779760
H	-4.952969	-5.084333	1.176342
H	-4.357039	-4.306618	-0.298701
H	-3.485282	-4.078973	1.226291
C	-5.625125	-2.888769	2.612016
H	-6.156241	-1.962506	2.854643
H	-6.252813	-3.737016	2.901883
H	-4.700016	-2.926740	3.194211
C	-6.604283	-2.913229	0.293842
H	-7.192316	-3.812432	0.504104
H	-7.212605	-2.041835	0.551463
H	-6.398505	-2.901614	-0.781237
H	-4.256714	-2.151487	-1.094584
S	-3.011402	-3.716897	-2.917815
C	-3.468341	-5.172789	-3.835576
H	-4.133541	-4.887094	-4.653759
H	-2.560266	-5.631143	-4.234909
H	-3.974912	-5.871612	-3.165815
O	-2.148488	-4.161380	-1.805884
O	-4.313487	-3.152126	-2.423986
O	-2.349783	-2.804609	-3.874809
C	3.470354	4.704331	0.959135
H	4.426706	4.667890	0.427207
H	3.222792	5.763094	1.102874

H	3.608949	4.254171	1.945757
C	0.930020	3.997781	-3.345009
H	0.151430	4.763454	-3.450839
H	1.839438	4.382943	-3.815851
H	0.598244	3.114062	-3.899790
C	-2.206753	1.135333	4.184166
H	-1.459038	1.241191	4.984653
H	-3.179111	1.038842	4.680668
C	-2.172946	2.389944	3.312037
H	-3.014549	2.378304	2.607969
H	-2.284813	3.285522	3.932029
H	-2.259048	-3.482681	3.429321
O	-0.950422	-3.499491	1.110731
C	-0.816648	-4.747808	1.768731
H	-0.287430	-4.621207	2.722579
H	-1.797094	-5.197857	1.972171
C	-0.032209	-5.669193	0.866933
H	-0.085645	-6.695464	1.262184
H	-0.476122	-5.664485	-0.139210
O	1.308644	-5.226850	0.823262
C	2.077961	-5.909498	-0.141749
H	1.664672	-5.776217	-1.152600
H	3.086190	-5.488669	-0.109547
H	2.131114	-6.986838	0.076054

B7uR

	Coordinates (Angstroms)		
	X	Y	Z
Ir	0.143522	-0.731570	-0.298208
C	1.094633	-0.173633	-1.983768
N	-0.109507	1.335931	-0.292732
C	1.768020	-0.988253	-2.917775
C	2.323401	-0.450437	-4.073547
C	2.294683	0.933754	-4.306198
C	1.693673	1.787714	-3.399956
C	1.089394	1.208241	-2.264540
C	0.369417	3.510131	-1.418697
C	0.409845	2.015053	-1.274702
C	-0.787657	1.958270	0.793576
C	-0.342361	1.712159	2.090580
C	-1.002181	2.255757	3.190847

C	-2.131386	3.048530	2.955735
C	-2.608464	3.293199	1.663571
C	-1.927066	2.732031	0.583474
O	-2.840185	3.571015	4.019886
C	-2.309928	4.790843	4.526187
H	2.757068	1.329132	-5.207529
H	0.344941	3.991767	-0.436120
H	0.519906	1.070442	2.243693
H	-2.300865	2.878251	-0.425807
H	-2.998065	5.132375	5.302766
H	-1.314882	4.646840	4.964606
H	-2.247404	5.552284	3.738885
C	-0.684426	-2.697394	-0.906422
C	0.037276	-2.834432	0.319446
C	-0.700911	-2.116436	1.333174
C	-1.822129	-1.513787	0.712706
C	-1.835705	-1.882198	-0.683305
C	-2.920158	-1.635532	-1.696616
C	-2.865600	-0.675581	1.405630
C	-3.511405	-0.243878	-1.685300
C	-4.239519	-1.274563	1.212221
C	-4.688263	-2.277826	2.114981
C	-5.905839	-2.883205	1.968476
C	-6.743544	-2.541947	0.876899
C	-6.305526	-1.562906	-0.058564
C	-5.038598	-0.923767	0.140280
C	-3.010933	0.730128	-2.605433
C	-3.593889	1.967900	-2.712288
C	-4.688266	2.314414	-1.876374
C	-5.155250	1.389669	-0.903824
C	-4.547858	0.091275	-0.841777
C	-5.330806	3.574748	-1.997577
C	-6.389894	3.905292	-1.188578
C	-6.838720	2.997562	-0.203057
C	-6.229648	1.774642	-0.058772
H	-0.411832	-3.141803	-1.853979
H	0.908909	-3.452782	0.477090
H	-0.451748	-2.044637	2.384160
H	-2.509708	-1.842226	-2.687649
H	-3.726154	-2.361138	-1.529317
H	-2.620587	-0.609561	2.467784
H	-2.853001	0.340300	1.006237
H	-3.246966	2.696760	-3.436763
H	-4.971517	4.273955	-2.748977

H	-6.879767	4.868995	-1.297579
H	-7.664938	3.271404	0.447015
H	-6.577265	1.086697	0.705685
O	-3.838524	-2.657149	3.132280
C	-4.154851	-2.085046	4.397296
H	-5.144441	-2.408558	4.743201
H	-3.394652	-2.436596	5.098229
H	-4.131270	-0.988495	4.352022
O	-1.963195	0.316199	-3.359891
C	-1.514026	1.163883	-4.401417
H	-1.154034	2.122399	-4.012177
H	-0.686897	0.638456	-4.880377
H	-2.312055	1.340313	-5.133364
H	-0.571059	3.777902	-1.920989
H	-6.221279	-3.644110	2.677366
C	-8.001029	-3.175227	0.685999
C	-8.787357	-2.862198	-0.393141
C	-7.137538	-1.274708	-1.175401
C	-8.345547	-1.905325	-1.336810
H	-9.746880	-3.352987	-0.530592
H	-8.326231	-3.916990	1.411307
H	-8.966656	-1.671855	-2.197150
H	-6.807368	-0.550018	-1.912673
C	-3.848544	4.119351	1.463413
H	-4.715454	3.654171	1.945165
H	-3.737466	5.118978	1.899508
H	-4.069130	4.236779	0.399611
C	-0.515803	1.978757	4.588264
H	0.213683	2.731446	4.912215
H	-1.340570	1.988776	5.307189
H	-0.021573	1.003242	4.639357
C	1.631765	3.283678	-3.595620
C	1.548009	4.016741	-2.256260
H	2.504458	3.624848	-4.163746
H	0.747678	3.539280	-4.199268
H	2.483162	3.869639	-1.701361
H	1.434290	5.093297	-2.420004
H	2.803390	-1.087664	-4.809198
O	1.868630	-2.313164	-2.584244
C	2.584426	-3.199463	-3.421586
H	3.546439	-2.765001	-3.723575
H	2.010513	-3.428879	-4.329489
C	2.816897	-4.459106	-2.615770
H	3.212181	-5.250980	-3.263680

H	1.875808	-4.804510	-2.178601
O	3.706536	-4.231494	-1.528931
C	5.078712	-4.369892	-1.860536
H	5.653927	-4.071080	-0.980379
H	5.308982	-5.414780	-2.103423
H	5.363991	-3.731059	-2.705520
H	1.548656	-0.378857	0.391490
C	6.629358	0.814701	1.053213
C	7.484531	1.870253	0.768317
C	6.925760	3.125068	0.568704
C	5.557420	3.333709	0.654311
C	4.739282	2.247075	0.941269
C	5.238028	0.954058	1.144412
H	8.555020	1.714775	0.701744
H	5.131989	4.319187	0.503402
Cl	3.025412	2.559751	1.056315
Cl	7.969285	4.472832	0.201435
Cl	7.353161	-0.761719	1.288274
C	4.350498	-0.224741	1.446825
H	3.342753	0.106726	1.722408
H	4.720369	-0.780089	2.313685
N	3.638663	-2.320267	0.511776
C	4.180309	-1.172996	0.297150
C	4.547748	-0.814144	-1.094825
H	4.010936	0.103079	-1.363929
H	5.618904	-0.600858	-1.161998
H	4.290493	-1.604614	-1.798227
O	3.122004	-2.598838	1.735083
C	3.642250	-3.832402	2.378012
C	5.163225	-3.816164	2.369650
H	5.528701	-4.675958	2.939987
H	5.568086	-3.899081	1.355454
H	5.551443	-2.907543	2.839862
C	3.081149	-3.685328	3.784436
H	1.988693	-3.612401	3.758679
H	3.353539	-4.562091	4.379974
H	3.483416	-2.792519	4.273938
C	3.074459	-5.057512	1.674447
H	3.433268	-5.958585	2.182815
H	1.980580	-5.058871	1.715119
H	3.391781	-5.118189	0.628285
H	3.461478	-3.004502	-0.254032

B7uS

	Coordinates (Angstroms) X	Y	Z
Ir	0.221280	0.032175	-0.193702
C	1.151247	1.761743	-0.648214
N	-0.526597	1.332989	1.251464
C	2.091331	2.008866	-1.669568
C	2.645725	3.271341	-1.846699
C	2.319887	4.329049	-0.985222
C	1.427888	4.136855	0.054555
C	0.853922	2.856143	0.192599
C	-0.466229	3.619354	2.243858
C	-0.090704	2.560683	1.247096
C	-1.465420	0.855847	2.207997
C	-1.140344	-0.261090	2.975124
C	-2.060297	-0.806522	3.868279
C	-3.315366	-0.196275	3.977069
C	-3.667291	0.919297	3.209903
C	-2.729279	1.432682	2.314736
O	-4.267215	-0.729367	4.823184
C	-4.149742	-0.295170	6.173724
H	2.786958	5.299593	-1.134825
H	-0.706853	3.166381	3.210647
H	-0.164582	-0.722983	2.854066
H	-2.992621	2.271384	1.676400
H	-4.997758	-0.722556	6.713385
H	-3.216850	-0.648514	6.629844
H	-4.189169	0.799055	6.243173
C	0.037823	-0.934527	-2.184777
C	0.610655	-1.853389	-1.253423
C	-0.413348	-2.168890	-0.285842
C	-1.569918	-1.415566	-0.605467
C	-1.311401	-0.655839	-1.800026
C	-2.289065	0.153054	-2.610403
C	-2.867889	-1.453337	0.157836
C	-3.215752	1.030752	-1.799276
C	-4.002791	-1.850580	-0.754677
C	-4.277017	-3.230798	-0.958109
C	-5.259601	-3.648215	-1.812379
C	-6.015930	-2.698364	-2.544802
C	-5.745164	-1.310669	-2.377242
C	-4.724378	-0.905055	-1.457964

C	-2.870855	2.403901	-1.592098
C	-3.720412	3.254975	-0.929926
C	-4.953401	2.773028	-0.414911
C	-5.290935	1.400382	-0.559072
C	-4.395058	0.542960	-1.279952
C	-5.864399	3.642564	0.241621
C	-7.056423	3.171451	0.734863
C	-7.381154	1.801480	0.615897
C	-6.514897	0.936836	-0.007948
H	0.534587	-0.521734	-3.051631
H	1.595173	-2.294686	-1.321185
H	-0.342025	-2.870906	0.532704
H	-1.721945	0.773539	-3.308026
H	-2.894343	-0.532565	-3.216573
H	-2.769380	-2.158308	0.985654
H	-3.076155	-0.473826	0.591725
H	-3.481959	4.304913	-0.797598
H	-5.604089	4.693678	0.341686
H	-7.748334	3.849459	1.226894
H	-8.316679	1.430333	1.024806
H	-6.769428	-0.115437	-0.089054
O	-3.492902	-4.152623	-0.296697
C	-4.089859	-4.677724	0.884618
H	-5.014161	-5.221117	0.652300
H	-3.364099	-5.367546	1.321050
H	-4.314309	-3.880033	1.604792
O	-1.678629	2.776003	-2.119418
C	-1.312083	4.142134	-2.054395
H	-1.244999	4.491151	-1.018151
H	-0.328628	4.209914	-2.521489
H	-2.025632	4.765184	-2.607855
H	-1.381817	4.114010	1.890658
H	-5.443883	-4.710769	-1.947826
C	-7.024219	-3.105764	-3.459010
C	-7.729461	-2.179290	-4.183666
C	-6.486784	-0.377487	-3.152697
C	-7.453531	-0.800354	-4.030026
H	-8.496868	-2.500875	-4.882131
H	-7.223242	-4.168078	-3.577700
H	-8.009826	-0.072751	-4.614395
H	-6.280047	0.683073	-3.048921
C	-5.038372	1.521854	3.342721
H	-5.818796	0.773457	3.169181
H	-5.198809	1.924936	4.350115

H	-5.175239	2.337011	2.627635
C	-1.720623	-2.031822	4.674343
H	-1.300486	-1.765269	5.651991
H	-2.610049	-2.643074	4.855088
H	-0.978269	-2.644965	4.154044
C	1.036898	5.232736	1.016455
C	0.649476	4.660328	2.380585
H	1.855002	5.954325	1.119307
H	0.179860	5.788943	0.607402
H	1.528083	4.194371	2.845649
H	0.315493	5.460799	3.048601
H	3.366230	3.449485	-2.638938
O	2.424346	0.937855	-2.457585
C	3.660637	0.945127	-3.143519
H	4.480436	1.162461	-2.444858
H	3.674972	1.696375	-3.944277
C	3.834631	-0.419305	-3.766955
H	4.773026	-0.430219	-4.343156
H	3.007012	-0.614671	-4.466395
O	3.859614	-1.401264	-2.754705
C	3.934500	-2.706563	-3.281666
H	3.916359	-3.404647	-2.441962
H	3.080200	-2.920753	-3.941015
H	4.864716	-2.856792	-3.849985
H	1.441989	-0.087468	0.839371
C	6.250272	1.175363	0.006755
C	7.603080	1.377534	-0.243222
C	8.407598	0.269511	-0.461146
C	7.890752	-1.016771	-0.429146
C	6.534987	-1.173992	-0.173011
C	5.663227	-0.098138	0.044490
H	8.014914	2.379642	-0.266259
H	8.523795	-1.879822	-0.598408
Cl	5.932050	-2.809716	-0.121915
Cl	10.107431	0.497200	-0.776503
Cl	5.285964	2.607032	0.268579
C	4.180627	-0.318479	0.263543
H	3.818081	-1.127347	-0.368991
H	3.626147	0.578145	-0.033024
N	3.227822	-1.631390	2.082205
C	3.819925	-0.555225	1.699349
C	4.064012	0.485987	2.732878
H	5.128416	0.738507	2.762648
H	3.506324	1.386824	2.453532

H	3.747123	0.166193	3.728401
O	2.840997	-2.606504	1.234307
C	2.583653	-3.908398	1.904004
C	1.418839	-3.752012	2.878432
H	1.081561	-4.745052	3.191060
H	1.691995	-3.210458	3.792221
H	0.582352	-3.238621	2.396665
C	2.208925	-4.788557	0.722764
H	3.012569	-4.803279	-0.019896
H	2.047040	-5.810801	1.077516
H	1.289813	-4.440549	0.243340
C	3.854059	-4.394889	2.587710
H	3.650229	-5.354090	3.073915
H	4.659941	-4.542506	1.863640
H	4.199627	-3.701586	3.362561
H	2.920799	-1.735234	3.050721

TSA2-3

	Coordinates (Angstroms)		
	X	Y	Z
Ir	-1.823224	0.245292	-0.613856
C	-2.987801	-1.402923	-0.376668
N	-1.568589	-0.198794	1.418998
C	-3.715467	-2.065187	-1.358747
C	-4.515801	-3.171397	-1.042667
C	-4.615202	-3.633918	0.276001
C	-3.895913	-2.978808	1.266296
C	-3.086758	-1.883611	0.954116
C	-2.272593	-1.543836	3.371354
C	-2.272083	-1.172864	1.921883
C	-0.626275	0.554067	2.178332
C	-0.705267	1.942873	2.196849
C	0.275828	2.701113	2.827864
C	1.354835	2.062422	3.444760
C	1.428249	0.665553	3.441557
C	0.444899	-0.080339	2.810610
O	2.378247	2.708386	4.065515
C	2.394570	4.125969	4.023957
H	-5.235888	-4.484160	0.533845
H	-1.819905	-0.772254	3.995582
H	-1.530299	2.442178	1.701617

H	0.525408	-1.160895	2.775523
H	3.304983	4.429194	4.543958
H	2.427936	4.494093	2.991356
H	1.526090	4.552987	4.539378
C	-1.185217	-0.121327	-2.670456
C	-1.593811	1.235287	-2.570505
C	-0.644418	1.917578	-1.718001
C	0.288564	0.978240	-1.251691
C	-0.053565	-0.326750	-1.800911
C	0.798425	-1.570751	-1.800036
C	1.463335	1.278724	-0.365531
C	1.422872	-1.932443	-0.470650
C	2.743276	0.891994	-1.066343
C	3.346934	1.825760	-1.963209
C	4.501757	1.514652	-2.633286
C	5.096358	0.235077	-2.465261
C	4.489700	-0.724177	-1.608750
C	3.291872	-0.359620	-0.907936
C	0.785503	-2.922224	0.343252
C	1.333249	-3.318163	1.537433
C	2.521287	-2.704628	2.015035
C	3.162132	-1.705981	1.234722
C	2.600734	-1.360824	-0.038473
C	3.060512	-3.041321	3.284241
C	4.182355	-2.409612	3.761255
C	4.816844	-1.409566	2.989338
C	4.318262	-1.067525	1.755902
H	-1.656693	-0.889297	-3.270294
H	-2.420935	1.695433	-3.093934
H	-0.662265	2.965632	-1.451321
H	0.182887	-2.401447	-2.151357
H	1.592579	-1.428715	-2.543066
H	1.460897	2.342597	-0.120868
H	1.380834	0.725054	0.570609
H	0.864721	-4.081281	2.149304
H	2.562929	-3.804973	3.877398
H	4.583363	-2.673004	4.736133
H	5.698415	-0.907340	3.377404
H	4.803300	-0.289290	1.174565
H	-1.722207	-2.479941	3.522124
H	4.973554	2.220841	-3.308116
C	6.286605	-0.112758	-3.157133
C	6.847709	-1.357063	-3.011456
C	5.085809	-2.007862	-1.490351

C	6.238311	-2.316307	-2.171272
H	7.759794	-1.609275	-3.545517
H	6.746374	0.626020	-3.809261
H	6.683898	-3.301472	-2.065533
H	4.622632	-2.754375	-0.852685
H	-3.333560	0.865759	-0.102589
S	-3.077648	4.221906	-0.141923
C	-4.215745	5.485690	0.373193
H	-3.965172	6.407316	-0.158300
H	-5.232673	5.174443	0.124837
H	-4.117086	5.636973	1.450249
O	-3.527216	3.001173	0.651958
H	-3.167351	1.768550	0.128436
O	-1.726427	4.654277	0.255726
O	-3.256206	4.012959	-1.588195
H	2.275730	0.178274	3.914821
H	0.188197	3.781631	2.811945
H	-3.968005	-3.341292	2.288186
H	-3.300064	-1.706654	3.710439
H	-3.699266	-1.739768	-2.395145
O	-5.168463	-3.730109	-2.092164
C	-6.026370	-4.834239	-1.851986
H	-6.839731	-4.565862	-1.167739
H	-6.446734	-5.099361	-2.823500
H	-5.472753	-5.691649	-1.451594
O	2.675763	2.999156	-2.082884
C	3.181663	3.975744	-2.976680
H	3.228759	3.588800	-4.001662
H	2.475801	4.806885	-2.935334
H	4.174559	4.323466	-2.666942
O	-0.359232	-3.435162	-0.172002
C	-0.952858	-4.538610	0.488447
H	-0.253542	-5.382020	0.542309
H	-1.285276	-4.272567	1.498106
H	-1.819041	-4.817776	-0.112674

TSA5-6uR

	Coordinates (Angstroms)		
	X	Y	Z
Ir	0.304951	0.381401	-0.789659
C	1.253579	2.039837	-0.152292

N	0.200095	0.180461	1.285064
C	1.824132	3.038492	-0.939209
C	2.567973	4.077353	-0.368032
C	2.746352	4.156950	1.021155
C	2.183139	3.172965	1.820534
C	1.448388	2.127632	1.253407
C	0.945016	0.986561	3.505444
C	0.832428	1.059842	2.014224
C	-0.632567	-0.823333	1.856302
C	-0.458265	-2.169372	1.524052
C	-1.345110	-3.122103	1.997445
C	-2.435821	-2.744755	2.791111
C	-2.615107	-1.402848	3.127243
C	-1.708183	-0.454999	2.658568
O	-3.265730	-3.751470	3.172556
C	-4.443995	-3.406759	3.883978
H	3.317797	4.958403	1.474460
H	0.626817	0.019250	3.896944
H	1.983847	1.159836	3.804354
H	0.342933	-2.466777	0.856175
H	-1.866786	0.593152	2.888978
H	-4.976000	-4.344803	4.051216
H	-4.210388	-2.946445	4.851489
H	-5.075304	-2.728989	3.296771
C	-0.496404	1.049320	-2.723047
C	0.159519	-0.202676	-2.900439
C	-0.600861	-1.206468	-2.194104
C	-1.688299	-0.556863	-1.561573
C	-1.647749	0.851469	-1.884572
C	-2.691429	1.900608	-1.603013
C	-2.744903	-1.243054	-0.738260
C	-3.259903	1.865367	-0.202209
C	-4.115965	-0.980157	-1.310023
C	-4.618582	-1.831281	-2.331265
C	-5.842580	-1.616932	-2.902385
C	-6.632513	-0.510846	-2.497419
C	-6.139053	0.371603	-1.494708
C	-4.861489	0.108827	-0.905174
C	-2.702530	2.726305	0.796201
C	-3.206498	2.747964	2.072631
C	-4.260131	1.868682	2.438089
C	-4.810836	0.986209	1.471095
C	-4.300113	1.026951	0.132322
C	-4.749548	1.825209	3.770192

C	-5.737277	0.941012	4.128764
C	-6.280267	0.056410	3.169012
C	-5.826490	0.079101	1.872101
H	-0.198288	1.992011	-3.164781
H	1.035556	-0.376802	-3.510675
H	-0.388603	-2.266223	-2.151746
H	-2.246061	2.879824	-1.791273
H	-3.507430	1.777556	-2.325888
H	-2.533781	-2.313313	-0.717183
H	-2.707490	-0.885056	0.291668
H	-2.804863	3.413916	2.829012
H	-4.325250	2.504914	4.505426
H	-6.102902	0.916916	5.151694
H	-7.058148	-0.643787	3.459897
H	-6.241767	-0.610634	1.143542
O	-3.815864	-2.866406	-2.760833
C	-4.162251	-4.134469	-2.213183
H	-5.167091	-4.441631	-2.528666
H	-3.429086	-4.849324	-2.593261
H	-4.120711	-4.116265	-1.116191
O	-1.675612	3.502289	0.370437
C	-1.105022	4.421959	1.284171
H	-0.697455	3.912190	2.163586
H	-0.293099	4.914276	0.746846
H	-1.843286	5.169181	1.599965
H	-1.227031	-4.170666	1.739646
H	0.335148	1.766046	3.976130
H	2.325962	3.231401	2.896338
H	1.737649	3.018173	-2.022482
O	3.104823	4.958373	-1.249369
C	3.911204	6.014482	-0.751495
H	4.250486	6.566994	-1.629308
H	3.334478	6.683712	-0.102502
H	4.782204	5.628621	-0.208875
H	-3.456364	-1.072371	3.726649
H	-6.201147	-2.283952	-3.681893
C	-7.899660	-0.254789	-3.086258
C	-8.642782	0.833358	-2.705650
C	-6.929274	1.496489	-1.131086
C	-8.148539	1.720172	-1.720237
H	-9.610056	1.020750	-3.163477
H	-8.268751	-0.937778	-3.847461
H	-8.738326	2.585918	-1.431731
H	-6.557507	2.188960	-0.382004

H	1.832647	-0.183864	-0.584639
C	6.251590	-1.095142	0.963508
C	7.626898	-1.213695	0.807793
C	8.271201	-0.321132	-0.035317
C	7.574812	0.678768	-0.697754
C	6.199603	0.760092	-0.516075
C	5.482939	-0.126002	0.303814
H	8.177362	-1.983716	1.335730
H	8.086763	1.383922	-1.342139
Cl	5.382331	2.061534	-1.349693
Cl	9.995560	-0.450648	-0.257472
Cl	5.499209	-2.217057	2.074662
C	3.978500	-0.044701	0.457184
H	3.635354	0.954799	0.188648
H	3.681185	-0.211630	1.496128
N	2.862099	-2.149669	0.117071
C	3.267000	-1.029152	-0.452500
C	3.549229	-1.019051	-1.921992
H	3.518776	0.002053	-2.302770
H	4.557989	-1.424166	-2.073248
H	2.836687	-1.636491	-2.466412
O	2.058448	-2.980308	-0.609357
C	2.507939	-4.382194	-0.633924
C	3.848824	-4.464636	-1.349040
H	3.772098	-4.071257	-2.367680
H	4.622156	-3.907146	-0.809218
H	4.169204	-5.509958	-1.406740
C	1.395091	-5.046797	-1.430365
H	1.612927	-6.112314	-1.549659
H	0.433236	-4.946657	-0.916194
H	1.311323	-4.600019	-2.426876
C	2.580522	-4.919926	0.789662
H	3.393914	-4.456368	1.358249
H	1.635094	-4.756099	1.318427
H	2.773964	-5.996955	0.761357
H	2.658349	-2.164768	1.115075

TSA5-6uS

	Coordinates (Angstroms)		
	X	Y	Z
Ir	0.308078	0.285302	-0.744017

C	1.200977	2.044792	-0.349903
N	0.084516	0.435391	1.321951
C	1.767064	2.931984	-1.265554
C	2.404114	4.104188	-0.842533
C	2.497913	4.421632	0.520978
C	1.951072	3.544571	1.446024
C	1.308106	2.375332	1.028387
C	0.724806	1.598951	3.412828
C	0.673241	1.432309	1.925728
C	-0.731550	-0.498922	2.020196
C	-0.494008	-1.869183	1.901825
C	-1.355672	-2.777162	2.504871
C	-2.465922	-2.318201	3.212963
C	-2.707106	-0.953517	3.335694
C	-1.839172	-0.046766	2.737087
O	-3.356047	-3.216576	3.759672
C	-2.964723	-3.713532	5.034334
H	2.988891	5.326292	0.859713
H	0.458177	0.681028	3.939219
H	1.734693	1.891377	3.715972
H	0.345465	-2.220867	1.311687
H	-2.037039	1.017501	2.799555
H	-3.733021	-4.426829	5.340443
H	-1.994956	-4.224513	4.981058
H	-2.906130	-2.903065	5.772465
C	-0.411663	0.649906	-2.783016
C	0.273892	-0.599454	-2.749842
C	-0.514958	-1.511961	-1.953437
C	-1.640662	-0.808731	-1.469913
C	-1.593725	0.548262	-1.973292
C	-2.664794	1.604239	-1.887984
C	-2.717846	-1.396610	-0.597507
C	-3.275254	1.774499	-0.515373
C	-4.078856	-1.225158	-1.227453
C	-4.567493	-2.214402	-2.124225
C	-5.785681	-2.089051	-2.733386
C	-6.586117	-0.942788	-2.496510
C	-6.107682	0.073509	-1.622179
C	-4.834908	-0.095545	-0.988545
C	-2.777761	2.802444	0.348056
C	-3.326809	3.011050	1.588600
C	-4.367490	2.165938	2.059437
C	-4.857874	1.122584	1.230576
C	-4.304471	0.968158	-0.082690

C	-4.904075	2.318948	3.364945
C	-5.877432	1.468524	3.829978
C	-6.357854	0.422741	3.009496
C	-5.858938	0.255290	1.740733
H	-0.111797	1.528305	-3.340659
H	1.175594	-0.842839	-3.296854
H	-0.284560	-2.549300	-1.748655
H	-2.234825	2.552298	-2.218407
H	-3.455654	1.349081	-2.603868
H	-2.499353	-2.453534	-0.434517
H	-2.712833	-0.909822	0.379581
H	-2.967987	3.798692	2.242571
H	-4.525495	3.119990	3.995456
H	-6.278961	1.595881	4.831636
H	-7.119462	-0.253759	3.386789
H	-6.226736	-0.556513	1.120889
O	-3.761083	-3.299938	-2.393600
C	-4.095258	-4.467412	-1.648732
H	-5.098535	-4.827370	-1.907818
H	-3.357397	-5.227533	-1.913723
H	-4.050862	-4.274804	-0.569033
O	-1.761574	3.535711	-0.169465
C	-1.281973	4.643321	0.571163
H	-0.885368	4.333480	1.543864
H	-0.476171	5.071888	-0.026189
H	-2.072156	5.391088	0.713450
H	-1.189081	-3.846359	2.407795
H	0.041053	2.391919	3.736701
H	2.025358	3.788885	2.502459
H	1.738000	2.735834	-2.334423
O	2.913792	4.878958	-1.833168
C	3.619229	6.057413	-1.477052
H	3.949755	6.502754	-2.416827
H	2.970896	6.766821	-0.949684
H	4.494638	5.825622	-0.858578
H	-3.583130	-0.607339	3.876302
H	-6.132584	-2.862087	-3.413981
C	-7.849639	-0.780452	-3.125762
C	-8.605013	0.342418	-2.902536
C	-6.910067	1.229936	-1.421988
C	-8.126818	1.359209	-2.043048
H	-9.569765	0.457129	-3.388811
H	-8.204880	-1.563521	-3.791238
H	-8.726127	2.250050	-1.877292

H	-6.552119	2.019830	-0.769176
H	1.811350	-0.247245	-0.369923
C	6.108225	0.836434	0.241730
C	7.435217	0.830111	0.653890
C	8.258601	-0.196077	0.215357
C	7.788058	-1.189855	-0.629501
C	6.454647	-1.145898	-1.017549
C	5.562569	-0.150640	-0.593657
H	7.814039	1.612447	1.301061
H	8.441219	-1.979411	-0.982680
Cl	5.924365	-2.401577	-2.113988
Cl	9.925337	-0.229126	0.726164
Cl	5.125927	2.173256	0.790340
C	4.105874	-0.137325	-1.009554
H	3.998262	-0.427110	-2.057842
H	3.703185	0.873448	-0.921114
N	2.988566	-2.233997	-0.630165
C	3.257243	-1.040147	-0.132161
C	3.251791	-0.834883	1.347949
H	4.219069	-1.173798	1.741107
H	3.140639	0.227256	1.570565
H	2.455611	-1.397911	1.832191
O	2.080721	-3.015888	0.024562
C	2.543954	-4.389508	0.274455
C	2.820036	-5.077245	-1.057032
H	1.954806	-4.996718	-1.724228
H	3.696652	-4.654784	-1.560271
H	3.023035	-6.138967	-0.885326
C	1.342135	-4.998648	0.982202
H	1.542678	-6.050834	1.206089
H	1.141939	-4.478682	1.924671
H	0.450063	-4.945076	0.348626
C	3.773387	-4.352831	1.170545
H	4.607199	-3.838349	0.680798
H	3.551498	-3.848549	2.116536
H	4.095597	-5.375419	1.392438
H	2.974527	-2.366290	-1.639261

TSA5f-1R

Coordinates (Angstroms)

X Y Z

Ir	0.059698	-0.379858	0.259228
C	0.760552	-2.155025	-0.378564
N	-0.432026	-0.339537	-1.775105
C	1.408095	-3.118533	0.388437
C	1.949464	-4.264135	-0.204855
C	1.811590	-4.497787	-1.580246
C	1.170363	-3.543014	-2.357265
C	0.653428	-2.383376	-1.776132
C	-0.166285	-1.390837	-4.003291
C	-0.014763	-1.331934	-2.514121
C	-1.230407	0.716887	-2.295336
C	-0.876431	2.044180	-2.074026
C	-1.704100	3.084363	-2.488557
C	-2.916477	2.792973	-3.118278
C	-3.277582	1.460753	-3.343335
C	-2.440325	0.435365	-2.936001
O	-3.809284	3.726793	-3.542087
C	-3.542918	5.088824	-3.252817
H	2.209627	-5.391347	-2.046299
H	-0.423418	-0.420485	-4.430807
H	0.770474	-1.731139	-4.455191
H	0.040701	2.272529	-1.542202
H	-2.740463	-0.595977	-3.083454
H	-4.400056	5.646415	-3.634128
H	-3.452999	5.256391	-2.172640
H	-2.632952	5.435857	-3.756999
C	-0.473329	-0.886627	2.323050
C	0.315163	0.297235	2.340804
C	-0.455825	1.348631	1.712030
C	-1.678905	0.794201	1.278427
C	-1.707297	-0.609896	1.640865
C	-2.879229	-1.555307	1.592282
C	-2.792451	1.537175	0.591852
C	-3.670345	-1.529498	0.304292
C	-4.071812	1.425819	1.384778
C	-4.322005	2.363465	2.432759
C	-5.471106	2.298320	3.178024
C	-6.416833	1.265958	2.936008
C	-6.169074	0.298457	1.924219
C	-4.969271	0.409771	1.146142
C	-3.380917	-2.499292	-0.708258
C	-4.099063	-2.537828	-1.877107
C	-5.103997	-1.565711	-2.128400
C	-5.386047	-0.574025	-1.151879

C	-4.665100	-0.601580	0.087066
C	-5.806369	-1.538690	-3.361979
C	-6.740533	-0.565087	-3.618972
C	-7.012664	0.429832	-2.652773
C	-6.351009	0.423630	-1.448963
H	-0.195508	-1.837984	2.757491
H	1.289479	0.396847	2.802113
H	-0.157381	2.381862	1.588994
H	-2.504588	-2.565127	1.772328
H	-3.547692	-1.310878	2.426968
H	-2.505771	2.582958	0.468069
H	-2.955202	1.125233	-0.405209
H	-3.901036	-3.284938	-2.638118
H	-5.585089	-2.300224	-4.106064
H	-7.267842	-0.553869	-4.568898
H	-7.744640	1.203478	-2.866366
H	-6.559193	1.197453	-0.716636
O	-3.341042	3.288083	2.607478
C	-3.524112	4.266452	3.616701
H	-3.616432	3.803980	4.606527
H	-2.630214	4.891953	3.593653
H	-4.407138	4.884275	3.414099
O	-2.376946	-3.356144	-0.401379
C	-2.066991	-4.393375	-1.314204
H	-1.792424	-3.994516	-2.296784
H	-1.211448	-4.917373	-0.885730
H	-2.910338	-5.087303	-1.419407
H	-1.400338	4.107570	-2.297271
H	-0.945674	-2.108862	-4.283559
H	1.078799	-3.718189	-3.425874
H	1.558237	-2.984091	1.454991
O	2.615329	-5.095069	0.638310
C	3.188891	-6.278240	0.108053
H	3.684507	-6.770983	0.946363
H	2.422278	-6.947447	-0.301220
H	3.931889	-6.052310	-0.665710
H	-4.228274	1.244333	-3.821625
H	-5.677706	3.013571	3.967011
C	-7.608575	1.172913	3.702423
C	-8.510489	0.161950	3.481658
C	-7.114262	-0.743274	1.728901
C	-8.257904	-0.809556	2.486765
H	-9.419967	0.102745	4.073256
H	-7.793901	1.918068	4.472270

H	-8.971829	-1.611779	2.322706
H	-6.925784	-1.496573	0.970185
H	1.588201	0.057072	-0.273388
C	5.682931	2.155052	0.576436
C	6.699910	3.053315	0.273291
C	6.361495	4.255476	-0.327809
C	5.041365	4.578218	-0.602861
C	4.057069	3.650465	-0.285242
C	4.330598	2.399655	0.291136
H	7.731011	2.818334	0.509971
H	4.780353	5.530078	-1.050135
Cl	2.406621	4.145982	-0.609089
Cl	7.620480	5.396149	-0.724894
Cl	6.164011	0.690178	1.393561
C	3.234898	1.396693	0.584162
H	2.290218	1.921664	0.711218
H	3.429646	0.864838	1.520618
N	3.764010	-0.734284	-0.388274
C	3.068818	0.385273	-0.552097
C	2.861158	0.872387	-1.958321
H	2.139776	1.687312	-1.973573
H	3.817215	1.239229	-2.350713
H	2.509456	0.067429	-2.604289
O	3.767558	-1.645423	-1.417955
C	5.112390	-2.077305	-1.798081
C	5.753084	-2.822509	-0.631110
H	6.667496	-3.322332	-0.967964
H	6.023816	-2.143545	0.183267
H	5.063333	-3.576909	-0.236522
C	4.835441	-3.012294	-2.967144
H	4.264824	-2.496071	-3.747247
H	5.782350	-3.351317	-3.398871
H	4.268750	-3.889560	-2.642221
C	5.942251	-0.878084	-2.241572
H	6.947048	-1.209907	-2.524507
H	5.486172	-0.387398	-3.108032
H	6.047615	-0.145281	-1.435465
H	3.866650	-1.164054	0.549064
S	3.500370	-1.642038	3.397331
C	4.692790	-2.247764	4.575274
H	5.660278	-1.782611	4.370707
H	4.361266	-1.988871	5.583823
H	4.771188	-3.332934	4.475444
O	2.216618	-2.301806	3.726211

O	4.035552	-2.047426	2.066146
O	3.455409	-0.170101	3.571709

TSA5f-1S

	Coordinates (Angstroms)		
	X	Y	Z
Ir	-0.047755	-0.446384	0.109594
C	-0.936476	-0.120281	1.882980
N	0.228285	1.615148	0.367040
C	-1.561019	-1.071940	2.686464
C	-2.214852	-0.702790	3.866124
C	-2.269432	0.639827	4.269816
C	-1.651639	1.598503	3.480084
C	-0.985234	1.235354	2.304825
C	-0.229093	3.630632	1.733006
C	-0.304538	2.166480	1.425024
C	1.061468	2.341031	-0.531190
C	0.831577	2.288667	-1.902377
C	1.693592	2.918060	-2.796501
C	2.815910	3.596205	-2.315663
C	3.055595	3.647045	-0.939088
C	2.185626	3.024385	-0.059117
O	3.729673	4.226913	-3.100661
C	3.573454	4.140710	-4.507379
H	-2.780424	0.937635	5.177875
H	-0.025057	4.227942	0.842151
H	-1.165334	3.975323	2.179026
H	-0.015452	1.732063	-2.285194
H	2.395882	3.039364	1.003811
H	4.423332	4.674896	-4.935580
H	3.593317	3.098413	-4.847995
H	2.643757	4.619652	-4.837554
C	0.664589	-2.491062	0.443765
C	-0.006972	-2.446755	-0.808196
C	0.786119	-1.633218	-1.704335
C	1.901920	-1.151750	-0.987443
C	1.836516	-1.664846	0.367592
C	2.901788	-1.611407	1.430623
C	2.990957	-0.270536	-1.538517
C	3.536872	-0.255817	1.637757
C	4.344112	-0.919738	-1.369322

C	4.821801	-1.806654	-2.382928
C	6.041897	-2.422031	-2.266140
C	6.836316	-2.210361	-1.107449
C	6.365058	-1.359900	-0.071101
C	5.097705	-0.708520	-0.237291
C	3.061228	0.581831	2.697065
C	3.649099	1.794420	2.957919
C	4.703128	2.268267	2.130990
C	5.165519	1.470202	1.051410
C	4.577081	0.179178	0.847002
C	5.279066	3.549583	2.336744
C	6.263228	4.021189	1.502521
C	6.714889	3.232482	0.420181
C	6.177963	1.987198	0.201779
H	0.353708	-3.058516	1.311091
H	-0.909725	-2.983484	-1.058952
H	0.562999	-1.421779	-2.742150
H	2.459956	-1.955413	2.368233
H	3.682419	-2.334816	1.164929
H	2.789952	-0.070677	-2.592900
H	2.989268	0.689357	-1.019112
H	3.311980	2.424026	3.774430
H	4.921285	4.156816	3.164905
H	6.694254	5.004576	1.669499
H	7.484376	3.615980	-0.243795
H	6.522922	1.392912	-0.638656
O	3.973016	-1.968146	-3.431433
C	4.382908	-2.803734	-4.500243
H	4.538021	-3.835225	-4.162182
H	3.569384	-2.781054	-5.227236
H	5.299670	-2.426728	-4.969231
O	2.026693	0.066441	3.404937
C	1.560480	0.774858	4.538703
H	1.192255	1.770202	4.266758
H	0.735664	0.182813	4.937412
H	2.349326	0.864274	5.296075
H	1.484632	2.856451	-3.858370
H	0.565682	3.824756	2.463341
H	-1.694001	2.636275	3.799158
H	-1.577003	-2.120451	2.403317
O	-2.781733	-1.720721	4.561946
C	-3.501938	-1.419843	5.746046
H	-3.876910	-2.374936	6.117864
H	-2.850545	-0.968646	6.503495

H	-4.348816	-0.754913	5.539816
H	3.939110	4.162069	-0.573572
H	6.418297	-3.088966	-3.034339
C	8.095081	-2.850402	-0.955822
C	8.847413	-2.664913	0.177404
C	7.159744	-1.199021	1.094591
C	8.371694	-1.833479	1.216356
H	9.807943	-3.162187	0.281265
H	8.450784	-3.497240	-1.754399
H	8.965276	-1.698980	2.116236
H	6.800679	-0.566425	1.900341
H	-1.563683	-0.013008	-0.441905
C	-4.890188	2.826894	-0.226441
C	-5.967927	3.624590	-0.591111
C	-7.162933	3.004274	-0.921215
C	-7.296961	1.625429	-0.874748
C	-6.192994	0.866054	-0.504380
C	-4.947682	1.425379	-0.180748
H	-5.872112	4.703897	-0.613357
H	-8.238952	1.146643	-1.115275
Cl	-6.429163	-0.860695	-0.424955
Cl	-8.530803	3.979776	-1.392510
Cl	-3.422354	3.676104	0.209552
C	-3.742916	0.582241	0.182281
H	-4.030528	-0.270758	0.801823
H	-3.049305	1.179557	0.773492
N	-3.294760	-1.155571	-1.415068
C	-3.006063	0.089933	-1.058051
C	-2.653704	1.077613	-2.132435
H	-2.215078	1.972544	-1.691012
H	-1.959996	0.642947	-2.850956
H	-3.573682	1.360386	-2.658912
O	-2.581223	-1.666254	-2.472197
C	-3.413603	-2.315182	-3.485912
C	-4.123069	-3.515538	-2.870243
H	-3.403256	-4.201503	-2.412777
H	-4.845549	-3.211611	-2.105919
H	-4.670390	-4.058744	-3.648137
C	-2.375828	-2.743492	-4.514243
H	-2.867237	-3.246731	-5.352823
H	-1.835709	-1.872795	-4.902337
H	-1.652175	-3.437301	-4.072807
C	-4.396684	-1.306719	-4.067291
H	-5.093213	-0.946726	-3.302939

H	-3.868833	-0.449576	-4.498142
H	-4.983883	-1.781470	-4.860312
H	-3.505172	-1.857898	-0.680576
S	-3.439653	-4.199871	1.029029
C	-3.976537	-4.492187	2.701128
H	-3.541055	-3.729087	3.351562
H	-5.067143	-4.436322	2.740030
H	-3.644606	-5.485909	3.012819
O	-4.095045	-5.220600	0.182676
O	-1.962451	-4.308978	1.042289
O	-3.909386	-2.815651	0.719521

TSA7-8uR

	Coordinates (Angstroms) X	Y	Z
Ir	0.500320	-0.737231	0.375649
C	1.130473	-2.353591	-0.640502
N	0.224238	-0.153285	-1.612652
C	1.579812	-3.559869	-0.105997
C	2.065435	-4.585562	-0.928547
C	2.136952	-4.418942	-2.318129
C	1.683949	-3.227306	-2.868369
C	1.175819	-2.212299	-2.053980
C	0.603359	-0.628997	-4.009499
C	0.644771	-0.955215	-2.549682
C	-0.444748	1.069629	-1.899028
C	0.062531	2.274375	-1.410578
C	-0.646005	3.450753	-1.586432
C	-1.882430	3.437750	-2.246364
C	-2.382368	2.239773	-2.756982
C	-1.655794	1.064213	-2.579813
O	-2.519046	4.635590	-2.336069
C	-3.813553	4.657254	-2.916564
H	2.523867	-5.198267	-2.964174
H	0.436425	0.434529	-4.187293
H	1.545287	-0.918172	-4.484843
H	1.007349	2.278816	-0.878588
H	-2.056153	0.126448	-2.948032
H	-4.164300	5.686710	-2.825396
H	-3.781898	4.378145	-3.976719
H	-4.499781	3.990421	-2.380927

C	-0.262863	-1.723361	2.177184
C	0.595274	-0.655845	2.564988
C	-0.057764	0.581175	2.195536
C	-1.265496	0.267452	1.542042
C	-1.414303	-1.175472	1.515361
C	-2.630385	-1.972986	1.121868
C	-2.255853	1.263331	1.004356
C	-3.298937	-1.533723	-0.162067
C	-3.604614	1.053661	1.650271
C	-3.877341	1.683232	2.903353
C	-5.080372	1.503260	3.536072
C	-6.063260	0.650834	2.964322
C	-5.797722	-0.015533	1.737035
C	-4.538727	0.212979	1.089434
C	-2.984135	-2.217018	-1.379924
C	-3.600922	-1.879893	-2.558763
C	-4.518103	-0.796204	-2.600064
C	-4.822853	-0.084116	-1.410026
C	-4.210764	-0.500907	-0.182921
C	-5.104072	-0.376880	-3.823605
C	-5.952399	0.702410	-3.864052
C	-6.253997	1.413797	-2.680203
C	-5.700372	1.029418	-1.482902
H	-0.090035	-2.777072	2.356649
H	1.525450	-0.753314	3.108952
H	0.306573	1.580024	2.387500
H	-2.337631	-3.022114	1.041279
H	-3.357459	-1.910127	1.941005
H	-1.886749	2.273622	1.191970
H	-2.351034	1.146368	-0.076487
H	-3.382125	-2.403851	-3.482722
H	-4.863892	-0.922857	-4.732974
H	-6.392793	1.014451	-4.807016
H	-6.925828	2.266672	-2.719078
H	-5.928586	1.586818	-0.579632
O	-2.856509	2.439399	3.383766
C	-3.023125	3.072516	4.640761
H	-3.199298	2.337296	5.435179
H	-2.085787	3.596931	4.834027
H	-3.846274	3.796596	4.616897
O	-2.061392	-3.203478	-1.258493
C	-1.735229	-3.964673	-2.407949
H	-1.319752	-3.333035	-3.200696
H	-0.980939	-4.683478	-2.085384

H	-2.614460	-4.499527	-2.787266
H	-0.263050	4.392725	-1.205002
H	-0.199860	-1.188679	-4.502992
H	1.728663	-3.102769	-3.946915
H	1.578196	-3.734075	0.965690
O	2.455579	-5.709940	-0.281401
C	3.095631	-6.737119	-1.022270
H	3.377995	-7.496703	-0.292028
H	2.415849	-7.178819	-1.760163
H	3.995420	-6.362829	-1.524161
H	-3.334430	2.193749	-3.273714
H	-5.301110	1.986821	4.481730
C	-7.309617	0.434868	3.609541
C	-8.245444	-0.411409	3.068463
C	-6.780294	-0.893903	1.208370
C	-7.976123	-1.086316	1.856221
H	-9.194778	-0.567718	3.573495
H	-7.508762	0.949470	4.546485
H	-8.717047	-1.762604	1.439102
H	-6.579915	-1.420039	0.279959
H	2.068152	-0.218184	0.135873
C	5.826013	2.250095	0.043439
C	6.352205	3.426979	-0.471415
C	5.556239	4.562818	-0.455557
C	4.278133	4.539318	0.079595
C	3.793360	3.337426	0.583026
C	4.526521	2.142811	0.563112
H	7.359876	3.454752	-0.869458
H	3.670355	5.435970	0.112422
Cl	2.203180	3.396986	1.306251
Cl	6.184022	6.056218	-1.100256
Cl	6.882468	0.856748	0.048676
C	3.988054	0.837911	1.101847
H	3.085712	1.015571	1.685279
H	4.712567	0.404751	1.796128
N	4.020219	-1.466384	0.329386
C	3.689138	-0.212350	0.030015
C	3.713264	0.128737	-1.431237
H	3.138925	1.037862	-1.618215
H	4.746433	0.295495	-1.754375
H	3.299950	-0.690477	-2.023724
O	3.828065	-1.843503	1.636064
C	4.846790	-2.766345	2.156044
C	6.220266	-2.114011	2.098002

H	6.956861	-2.775881	2.565256
H	6.531592	-1.943972	1.062987
H	6.228448	-1.160466	2.635364
C	4.369351	-2.962251	3.587724
H	3.359109	-3.385405	3.603075
H	5.039829	-3.650100	4.111744
H	4.360373	-2.008562	4.126123
C	4.816217	-4.075864	1.375687
H	5.556052	-4.763653	1.798616
H	3.834804	-4.551419	1.439108
H	5.074952	-3.932952	0.320071
H	3.747389	-2.198079	-0.327937

TSA7-8uS

	Coordinates (Angstroms) X	Y	Z
Ir	-0.404430	-0.132580	-0.521221
C	-1.317657	-1.927677	-0.501855
N	-0.027930	-0.798017	1.420051
C	-1.975036	-2.554382	-1.559394
C	-2.588980	-3.801818	-1.396571
C	-2.572727	-4.457291	-0.156583
C	-1.931181	-3.843038	0.909700
C	-1.304220	-2.604556	0.748032
C	-0.470994	-2.476163	3.182757
C	-0.570370	-1.922838	1.796089
C	0.838020	-0.039953	2.256192
C	0.573565	1.310561	2.499111
C	1.478818	2.084742	3.204487
C	2.675829	1.523193	3.668937
C	2.936222	0.170750	3.449176
C	2.013152	-0.599106	2.744648
O	3.518172	2.372351	4.313986
C	4.788682	1.876412	4.706525
H	-3.047361	-5.421306	-0.016607
H	-0.145512	-1.723685	3.903006
H	-1.442507	-2.866576	3.500191
H	-0.333861	1.758868	2.110353
H	2.230648	-1.641982	2.544332
H	5.320431	2.726154	5.137658
H	4.696066	1.087157	5.462377

H	5.347449	1.494122	3.843787
C	0.178142	-0.058513	-2.642741
C	-0.482290	1.158569	-2.308571
C	0.375731	1.874664	-1.393458
C	1.511084	1.081006	-1.134016
C	1.411462	-0.137228	-1.911436
C	2.471248	-1.185499	-2.130543
C	2.654974	1.469279	-0.237457
C	3.200734	-1.626491	-0.881333
C	3.952194	1.455861	-1.008294
C	4.341975	2.617350	-1.729312
C	5.492042	2.648108	-2.468914
C	6.317071	1.497286	-2.548122
C	5.932755	0.312279	-1.858482
C	4.727976	0.316884	-1.084532
C	2.765829	-2.813067	-0.209684
C	3.423593	-3.279928	0.900422
C	4.513121	-2.547898	1.442605
C	4.942992	-1.350275	0.811855
C	4.276732	-0.924268	-0.384016
C	5.155085	-2.968029	2.637098
C	6.175467	-2.230064	3.185097
C	6.600770	-1.034885	2.561367
C	5.997737	-0.606408	1.403334
H	-0.176285	-0.802685	-3.344964
H	-1.407718	1.523064	-2.733985
H	0.197071	2.856718	-0.981058
H	2.000051	-2.051124	-2.601790
H	3.199668	-0.790681	-2.849741
H	2.460254	2.463112	0.171098
H	2.723333	0.777394	0.604430
H	3.113177	-4.190340	1.401732
H	4.818246	-3.884169	3.116307
H	6.657285	-2.562211	4.100488
H	7.406817	-0.454310	3.001014
H	6.324598	0.316998	0.935265
O	3.499201	3.707387	-1.692278
C	3.927614	4.742619	-0.813072
H	4.875958	5.179771	-1.148562
H	3.148478	5.507861	-0.830463
H	4.047379	4.364936	0.210579
O	1.688607	-3.418774	-0.766779
C	1.251961	-4.648797	-0.217212
H	0.947132	-4.534064	0.828830

H	0.389657	-4.952758	-0.811824
H	2.036713	-5.411721	-0.290072
H	1.286546	3.137427	3.390600
H	0.243056	-3.307695	3.210436
H	-1.914529	-4.350740	1.870495
H	-2.042681	-2.088339	-2.538799
O	-3.191133	-4.298715	-2.506195
C	-3.868984	-5.541683	-2.414500
H	-4.294860	-5.720999	-3.403174
H	-3.178911	-6.355663	-2.163281
H	-4.677791	-5.500257	-1.675370
H	3.850165	-0.296928	3.797837
H	5.765201	3.549729	-3.010948
C	7.511820	1.498248	-3.317191
C	8.290522	0.372555	-3.404191
C	6.755483	-0.840943	-1.980596
C	7.904146	-0.810295	-2.730939
H	9.203760	0.383530	-3.992685
H	7.795988	2.409532	-3.837759
H	8.520454	-1.701234	-2.812333
H	6.467813	-1.757240	-1.474667
H	-1.838720	0.339920	0.158052
C	-6.156715	-0.787038	0.110597
C	-7.524649	-0.882573	0.337913
C	-8.309836	0.239616	0.121090
C	-7.759417	1.427090	-0.334647
C	-6.386715	1.480083	-0.545218
C	-5.527972	0.396623	-0.310263
H	-7.963998	-1.816327	0.669179
H	-8.379884	2.293491	-0.531379
Cl	-5.759805	2.980725	-1.178086
Cl	-10.028471	0.148454	0.403355
Cl	-5.235578	-2.256477	0.329790
C	-4.029446	0.482333	-0.536790
H	-3.801842	1.171078	-1.350379
H	-3.654301	-0.500050	-0.828175
N	-2.982921	2.178895	0.926895
C	-3.268072	0.905485	0.712377
C	-3.362312	0.078336	1.961449
H	-4.374133	0.153406	2.376200
H	-3.160061	-0.966459	1.725412
H	-2.646441	0.415857	2.715509
O	-2.655037	2.981513	-0.130895
C	-2.367478	4.370327	0.260226

C	-1.158508	4.408467	1.196842
H	-0.788297	5.436513	1.259244
H	-1.405127	4.104132	2.221030
H	-0.348864	3.775927	0.822435
C	-2.046401	5.008842	-1.083264
H	-2.884369	4.890457	-1.777649
H	-1.863240	6.078417	-0.943324
H	-1.153326	4.562365	-1.531699
C	-3.589856	5.008077	0.908590
H	-3.336730	6.025258	1.225061
H	-4.426254	5.065239	0.207954
H	-3.913656	4.454943	1.796951
H	-2.496440	2.413005	1.789583

TSA7f-1R

	Coordinates (Angstroms) X	Y	Z
Ir	0.203440	0.352760	0.458771
C	1.027803	-0.700694	1.964249
N	-0.038746	-1.618578	-0.191266
C	1.593159	-0.195335	3.133269
C	2.166521	-1.043485	4.087679
C	2.196614	-2.431480	3.894684
C	1.628042	-2.951796	2.740790
C	1.042334	-2.111608	1.789740
C	0.237133	-4.034675	0.272378
C	0.400863	-2.577239	0.576171
C	-0.766863	-1.843707	-1.393065
C	-0.397446	-1.168725	-2.562385
C	-1.167936	-1.284420	-3.706657
C	-2.334587	-2.061367	-3.700261
C	-2.704991	-2.741772	-2.541236
C	-1.916438	-2.625418	-1.398297
O	-3.043152	-2.079896	-4.861176
C	-4.286007	-2.762971	-4.866717
H	2.646679	-3.098089	4.621040
H	0.100855	-4.222481	-0.794623
H	1.097937	-4.606447	0.625909
H	0.475417	-0.522529	-2.562590
H	-2.232401	-3.117581	-0.486748
H	-4.707746	-2.610137	-5.861668

H	-4.152200	-3.837345	-4.692589
H	-4.970510	-2.351156	-4.114842
C	-0.613690	2.004921	1.625941
C	0.059557	2.547041	0.496768
C	-0.692707	2.175507	-0.680703
C	-1.778643	1.379590	-0.278048
C	-1.738624	1.230576	1.165047
C	-2.801862	0.657672	2.068518
C	-2.833040	0.831928	-1.198690
C	-3.417034	-0.642389	1.597875
C	-4.190728	1.334496	-0.770614
C	-4.645604	2.605364	-1.238551
C	-5.859492	3.108439	-0.845051
C	-6.672264	2.376378	0.062749
C	-6.221791	1.126252	0.567442
C	-4.955897	0.622240	0.122205
C	-2.950592	-1.877028	2.151770
C	-3.498279	-3.077244	1.771205
C	-4.509640	-3.117581	0.775053
C	-4.984058	-1.906021	0.206699
C	-4.434200	-0.666323	0.668992
C	-5.028683	-4.351538	0.301528
C	-5.973829	-4.381220	-0.694466
C	-6.446021	-3.175433	-1.261947
C	-5.962064	-1.967690	-0.820277
H	-0.333342	2.147551	2.661520
H	0.931544	3.185482	0.532700
H	-0.470166	2.441358	-1.703895
H	-2.361802	0.516313	3.057925
H	-3.594720	1.408521	2.176217
H	-2.610737	1.140762	-2.222020
H	-2.826433	-0.258811	-1.176349
H	-3.158326	-4.013449	2.200140
H	-4.657056	-5.276254	0.736593
H	-6.360109	-5.332017	-1.051538
H	-7.193001	-3.205605	-2.050186
H	-6.322616	-1.046570	-1.267657
O	-3.778795	3.237214	-2.071653
C	-4.128663	4.522336	-2.555770
H	-4.248283	5.237863	-1.733424
H	-3.297929	4.836358	-3.190094
H	-5.047916	4.487329	-3.152812
O	-1.967882	-1.752541	3.076727
C	-1.585659	-2.905768	3.805191

H	-1.147425	-3.667707	3.151056
H	-0.833431	-2.571038	4.520142
H	-2.443169	-3.330143	4.341800
H	-0.893189	-0.756913	-4.615337
H	-0.646260	-4.422311	0.794976
H	1.643818	-4.028553	2.596635
H	1.638064	0.872825	3.322476
O	2.686139	-0.421571	5.177884
C	3.276166	-1.213007	6.195685
H	3.604377	-0.510956	6.964025
H	2.551461	-1.912133	6.630026
H	4.144255	-1.766823	5.818862
H	-3.606988	-3.341792	-2.495442
H	-6.218395	4.068482	-1.200464
C	-7.927808	2.880548	0.494296
C	-8.696526	2.181157	1.391560
C	-7.033050	0.432322	1.503537
C	-8.241933	0.945558	1.905309
H	-9.654480	2.579693	1.714576
H	-8.270690	3.835205	0.102163
H	-8.851518	0.401873	2.621595
H	-6.688435	-0.513922	1.909313
H	1.756315	0.248050	-0.225755
C	5.972973	-0.588274	-1.739166
C	7.152021	-1.300500	-1.560175
C	7.063871	-2.639854	-1.212252
C	5.836293	-3.263360	-1.058974
C	4.683228	-2.509089	-1.245453
C	4.695305	-1.146207	-1.575707
H	8.113061	-0.817751	-1.694831
H	5.770462	-4.314785	-0.804683
Cl	3.172640	-3.368713	-1.077685
Cl	8.526507	-3.561954	-0.980290
Cl	6.142968	1.084889	-2.207700
C	3.424114	-0.346923	-1.771408
H	2.573422	-1.025260	-1.804253
H	3.451933	0.163493	-2.737823
N	2.997342	1.960353	-1.113026
C	3.187856	0.713846	-0.688717
C	3.878179	0.594683	0.642283
H	3.807057	-0.431909	1.006881
H	4.936609	0.856275	0.536513
H	3.410622	1.255674	1.374273
O	2.171983	2.079779	-2.207222

C	2.358919	3.295016	-3.005078
C	1.556194	2.967193	-4.258064
H	1.578613	3.816393	-4.948208
H	1.973958	2.093476	-4.769594
H	0.509945	2.757648	-4.008610
C	1.769335	4.494624	-2.267862
H	2.298207	4.700717	-1.332558
H	1.854991	5.385207	-2.899864
H	0.709186	4.339409	-2.045738
C	3.831788	3.507124	-3.322290
H	3.936325	4.371130	-3.986854
H	4.408010	3.709728	-2.413638
H	4.252077	2.632344	-3.827335
H	2.925425	2.721963	-0.419233
S	3.259642	4.128273	2.193796
C	4.918259	3.722705	2.703245
H	5.610695	4.463034	2.294800
H	4.965777	3.732586	3.794949
H	5.176500	2.728498	2.330572
O	2.385762	3.075208	2.759928
O	3.286881	4.088375	0.699164
O	2.976701	5.481164	2.712612

TSA7f-1S

	Coordinates (Angstroms) X	Y	Z
Ir	0.071197	-0.695928	-0.199577
C	-0.918525	-0.930065	1.536678
N	0.203399	1.206529	0.637110
C	-1.526153	-2.094264	1.998279
C	-2.219902	-2.110946	3.211860
C	-2.320755	-0.957093	4.001833
C	-1.721058	0.210434	3.552659
C	-1.025579	0.235321	2.340669
C	-0.367200	2.727456	2.500690
C	-0.372823	1.407484	1.793115
C	0.955982	2.212493	-0.033953
C	0.697500	2.492597	-1.372948
C	1.485522	3.396132	-2.080256
C	2.562803	4.018990	-1.444684
C	2.827896	3.740867	-0.100111

C	2.030617	2.846226	0.595436
O	3.408398	4.896863	-2.046129
C	3.200338	5.194023	-3.417028
H	-2.855806	-0.958984	4.944270
H	-0.286035	3.561390	1.799887
H	-1.276374	2.856951	3.090103
H	-0.113732	1.982480	-1.881867
H	2.265501	2.609379	1.626044
H	3.985391	5.900344	-3.691726
H	3.289136	4.293850	-4.037324
H	2.221257	5.660491	-3.580066
C	0.944254	-2.695581	-0.359796
C	0.349654	-2.374023	-1.608390
C	1.103542	-1.285314	-2.186864
C	2.114349	-0.916639	-1.280480
C	2.024471	-1.777348	-0.113139
C	3.025966	-1.926652	1.002854
C	3.142979	0.154766	-1.509526
C	3.560178	-0.632547	1.574810
C	4.530894	-0.433265	-1.452737
C	5.100930	-0.992891	-2.628508
C	6.346476	-1.557159	-2.620007
C	7.090575	-1.620013	-1.414006
C	6.526431	-1.096823	-0.215866
C	5.226672	-0.497356	-0.263050
C	2.982144	-0.115528	2.778334
C	3.459338	1.034113	3.357332
C	4.505761	1.765158	2.733829
C	5.080271	1.282310	1.528153
C	4.603200	0.045382	0.982590
C	4.962595	2.997701	3.270434
C	5.940592	3.722419	2.634330
C	6.507148	3.246797	1.430029
C	6.086329	2.055335	0.891629
H	0.637254	-3.495799	0.301632
H	-0.458840	-2.914347	-2.080672
H	0.924777	-0.821759	-3.148542
H	2.561157	-2.511636	1.799367
H	3.865293	-2.520483	0.620043
H	2.957121	0.622918	-2.477834
H	3.051127	0.931569	-0.749609
H	3.038702	1.418750	4.280138
H	4.516858	3.364728	4.192002
H	6.279056	4.665850	3.053980

H	7.274419	3.829011	0.927616
H	6.519973	1.702618	-0.038859
O	4.339096	-0.993793	-3.776527
C	4.662835	0.038505	-4.701600
H	5.682526	-0.080080	-5.088381
H	3.948358	-0.048777	-5.523241
H	4.566500	1.029425	-4.238990
O	1.969278	-0.859479	3.285922
C	1.458341	-0.519408	4.562237
H	0.996954	0.474087	4.559321
H	0.696842	-1.267692	4.785552
H	2.248545	-0.556526	5.321963
H	1.257323	3.588661	-3.122389
H	0.480405	2.780659	3.194282
H	-1.798708	1.101887	4.169017
H	-1.520397	-3.005025	1.407739
O	-2.790247	-3.299663	3.538800
C	-3.637877	-3.356391	4.674466
H	-4.035384	-4.372509	4.697046
H	-3.081398	-3.166852	5.599936
H	-4.467055	-2.644123	4.589124
H	3.676647	4.217693	0.381822
H	6.756771	-1.977756	-3.534335
C	8.380961	-2.212915	-1.372558
C	9.080050	-2.290721	-0.195056
C	7.272561	-1.203695	0.989703
C	8.516409	-1.783506	0.999153
H	10.065363	-2.747700	-0.173974
H	8.803496	-2.608593	-2.292927
H	9.071147	-1.857869	1.930305
H	6.847791	-0.822617	1.913206
H	-1.467164	-0.168545	-0.727570
C	-5.123223	2.332679	-1.927392
C	-6.427356	2.807271	-1.846472
C	-6.945702	3.094393	-0.594230
C	-6.173370	2.961369	0.549119
C	-4.876226	2.478336	0.420028
C	-4.313698	2.091505	-0.807841
H	-7.014241	2.959718	-2.744565
H	-6.566196	3.232160	1.522143
Cl	-3.939497	2.424137	1.891565
Cl	-8.586550	3.671350	-0.456794
Cl	-4.493632	2.109010	-3.543207
C	-2.916546	1.512575	-0.903411

H	-2.423654	1.638976	0.059270
H	-2.327636	2.075778	-1.631765
N	-3.672940	-0.824441	-0.733993
C	-2.822761	0.030207	-1.297376
C	-2.467327	-0.358949	-2.720236
H	-3.358621	-0.322677	-3.350625
H	-1.718394	0.330031	-3.114397
H	-2.071739	-1.374090	-2.754702
O	-4.099935	-0.579646	0.551721
C	-5.379809	-1.230238	0.848799
C	-5.172302	-2.737910	0.991552
H	-6.107661	-3.196839	1.330723
H	-4.904429	-3.205637	0.040534
H	-4.394687	-2.960446	1.727918
C	-5.772252	-0.618193	2.186302
H	-6.039360	0.436587	2.078253
H	-6.638996	-1.150507	2.590742
H	-4.948822	-0.699786	2.903398
C	-6.406237	-0.911548	-0.233281
H	-7.379233	-1.316851	0.064233
H	-6.513429	0.168933	-0.370292
H	-6.134208	-1.363021	-1.192833
H	-3.662137	-1.812777	-1.028095
S	-2.899160	-4.342149	-1.933010
C	-3.796843	-5.881317	-1.961560
H	-4.311029	-5.979999	-2.920600
H	-3.089416	-6.703745	-1.831102
H	-4.523993	-5.882649	-1.145865
O	-2.232875	-4.268707	-0.614439
O	-3.930598	-3.274336	-2.099574
O	-1.954297	-4.383209	-3.072087

TSB2-3

	Coordinates (Angstroms) X	Y	Z
Ir	1.649211	-0.023833	-0.310411
C	2.529474	1.799178	-0.524273
N	0.903675	1.032745	1.333374
C	3.450625	2.197803	-1.502880
C	4.003588	3.477516	-1.480910
C	3.689675	4.376414	-0.456648

C	2.807878	4.017912	0.551920
C	2.236867	2.733622	0.484949
C	0.938812	3.153118	2.642063
C	1.315476	2.255581	1.499604
C	-0.018210	0.410008	2.226344
C	0.311002	-0.822098	2.783524
C	-0.587816	-1.495021	3.609484
C	-1.826025	-0.895331	3.863826
C	-2.182018	0.336709	3.304592
C	-1.267762	0.977981	2.470122
O	-2.760770	-1.544499	4.645729
C	-2.577018	-1.357734	6.044480
H	4.154562	5.359136	-0.450471
H	0.688218	2.563287	3.528405
H	1.271531	-1.275363	2.561061
H	-1.539334	1.912746	1.988713
H	-3.417763	-1.850418	6.538340
H	-1.639923	-1.810007	6.391329
H	-2.575643	-0.291665	6.304229
C	1.353983	-0.508258	-2.433077
C	1.935945	-1.634439	-1.795830
C	0.963676	-2.156387	-0.858869
C	-0.168714	-1.321526	-0.882703
C	0.067537	-0.255498	-1.841229
C	-0.944686	0.696777	-2.417020
C	-1.429597	-1.515671	-0.087754
C	-1.885971	1.349491	-1.431858
C	-2.595299	-1.717156	-1.026024
C	-2.865814	-3.027777	-1.507357
C	-3.885754	-3.271755	-2.384429
C	-4.678151	-2.200475	-2.868445
C	-4.403839	-0.873437	-2.431127
C	-3.351950	-0.654332	-1.481404
C	-1.588643	2.673512	-0.980729
C	-2.468078	3.366576	-0.187609
C	-3.678426	2.755059	0.233335
C	-3.960998	1.413507	-0.140982
C	-3.044299	0.732427	-1.010746
C	-4.619166	3.465801	1.024858
C	-5.785000	2.869435	1.437087
C	-6.051735	1.524597	1.095502
C	-5.158465	0.813604	0.331393
H	1.810088	0.094444	-3.204875
H	2.909975	-2.053792	-2.002547

H	1.090099	-3.021524	-0.220964
H	-0.405929	1.470977	-2.968143
H	-1.537143	0.137384	-3.151496
H	-1.302767	-2.376200	0.571632
H	-1.615330	-0.644046	0.540708
H	-2.269976	4.386940	0.122676
H	-4.401383	4.496537	1.294382
H	-6.499369	3.426428	2.037054
H	-6.964475	1.050197	1.444442
H	-5.368786	-0.221772	0.082236
H	0.029016	3.700989	2.357039
H	-4.071109	-4.284944	-2.731170
C	-5.724355	-2.424587	-3.803366
C	-6.461272	-1.378337	-4.295915
C	-5.175004	0.189956	-2.976371
C	-6.176713	-0.056098	-3.881641
H	-7.258256	-1.559627	-5.011666
H	-5.926465	-3.443612	-4.124049
H	-6.753280	0.770471	-4.287380
H	-4.962371	1.211193	-2.677272
C	-3.535486	0.926032	3.587209
H	-4.335466	0.246734	3.274841
H	-3.673517	1.110871	4.659247
H	-3.663766	1.875355	3.061709
C	-0.231361	-2.837841	4.189602
H	0.273357	-2.731490	5.158036
H	-1.123920	-3.450647	4.347466
H	0.449915	-3.375958	3.523310
C	2.447280	4.941368	1.689825
C	2.058498	4.154753	2.941436
H	3.284950	5.613163	1.906749
H	1.603798	5.581307	1.389991
H	2.934863	3.617390	3.325379
H	1.725542	4.838121	3.729362
H	4.701021	3.782486	-2.255923
O	3.772130	1.278008	-2.462308
C	5.064963	1.313567	-3.043881
H	5.805769	1.637264	-2.302006
H	5.093924	2.004580	-3.896388
C	5.383981	-0.087363	-3.511460
H	6.352475	-0.075528	-4.035284
H	4.619008	-0.434569	-4.223758
O	5.430815	-0.935893	-2.385723
C	5.769611	-2.264192	-2.720011

H	5.737480	-2.846222	-1.796496
H	5.056134	-2.693414	-3.439504
H	6.779943	-2.324652	-3.152750
H	3.083338	-0.114735	0.594322
S	3.564343	-3.283501	1.494915
C	4.864726	-3.932645	2.516261
H	5.044648	-4.970605	2.225373
H	5.767423	-3.337538	2.362435
H	4.553983	-3.886051	3.562407
O	3.392860	-1.859500	2.010184
H	2.978192	-0.936679	1.077266
O	2.357570	-4.092533	1.744337
O	4.032195	-3.294691	0.099879
O	-2.038542	-4.048698	-1.094499
C	-2.576735	-4.851606	-0.048823
H	-3.479635	-5.378841	-0.380919
H	-1.805138	-5.578382	0.212868
H	-2.819697	-4.244001	0.832514
O	-0.411498	3.172468	-1.430181
C	-0.106015	4.526067	-1.147795
H	-0.858326	5.196436	-1.581327
H	-0.035681	4.703697	-0.068906
H	0.863322	4.715926	-1.609664

TSB5-6uR

	Coordinates (Angstroms) X	Y	Z
Ir	-0.129798	0.867465	-0.460353
C	-1.013423	0.471278	-2.228271
N	-0.038658	-1.214661	-0.553108
C	-1.606297	1.387156	-3.117827
C	-2.229429	0.951100	-4.283908
C	-2.311124	-0.416466	-4.584779
C	-1.772667	-1.361130	-3.728286
C	-1.126763	-0.890616	-2.568300
C	-0.672709	-3.283260	-1.791224
C	-0.567363	-1.799827	-1.589441
C	0.525981	-1.938474	0.534252
C	0.060339	-1.670599	1.818864
C	0.622750	-2.286112	2.933144
C	1.673421	-3.187408	2.723359

C	2.163782	-3.468192	1.443223
C	1.583692	-2.825120	0.349140
O	2.300804	-3.772817	3.803790
C	1.644790	-4.930593	4.307942
H	-2.819207	-0.729120	-5.493791
H	-0.723528	-3.796521	-0.826208
H	-0.730536	-0.939961	1.947798
H	1.980414	-2.992226	-0.647877
H	2.264611	-5.309072	5.123687
H	0.646587	-4.691491	4.694281
H	1.552483	-5.702472	3.533713
C	0.827054	2.804958	-0.879222
C	0.041176	2.917978	0.304920
C	0.670598	2.096795	1.317848
C	1.782403	1.447790	0.734548
C	1.897993	1.883541	-0.642290
C	3.018906	1.617144	-1.610130
C	2.718885	0.498762	1.436540
C	3.505263	0.185986	-1.654728
C	4.142065	0.998600	1.345634
C	4.618438	1.913057	2.325081
C	5.878244	2.440870	2.262414
C	6.739508	2.101523	1.188427
C	6.281168	1.202152	0.185020
C	4.964174	0.647708	0.292241
C	2.999507	-0.694480	-2.663316
C	3.488854	-1.970077	-2.798150
C	4.490810	-2.447558	-1.911614
C	4.960833	-1.612666	-0.862579
C	4.453879	-0.274441	-0.768294
C	5.041608	-3.748340	-2.058516
C	6.012659	-4.202402	-1.200229
C	6.460290	-3.383675	-0.139051
C	5.939872	-2.123687	0.029848
H	0.648732	3.321610	-1.811717
H	-0.815149	3.564178	0.440582
H	0.340028	1.974900	2.341211
H	2.683846	1.918788	-2.605137
H	3.862320	2.268896	-1.349740
H	2.407792	0.403674	2.478796
H	2.651822	-0.493479	0.985702
H	3.137519	-2.632102	-3.582439
H	4.684976	-4.378045	-2.870209
H	6.433807	-5.195686	-1.329544

H	7.214305	-3.755367	0.549000
H	6.285965	-1.504885	0.851919
O	3.757200	2.290820	3.333910
C	3.942480	1.570032	4.548263
H	4.945881	1.738117	4.958543
H	3.195722	1.944186	5.252092
H	3.793782	0.492968	4.396462
O	2.048360	-0.155777	-3.465105
C	1.571892	-0.923063	-4.555993
H	1.106625	-1.854947	-4.216261
H	0.821152	-0.304116	-5.049162
H	2.382838	-1.151874	-5.258302
H	0.246941	-3.629407	-2.283721
H	6.210640	3.138624	3.026493
C	8.042223	2.658536	1.082500
C	8.855321	2.344328	0.023683
C	7.145302	0.906918	-0.905133
C	8.398194	1.461172	-0.982320
H	9.848596	2.778247	-0.050105
H	8.380371	3.344156	1.855572
H	9.043713	1.223606	-1.823337
H	6.805238	0.236963	-1.688237
C	3.316573	-4.417648	1.271184
H	4.216897	-4.036604	1.764855
H	3.098111	-5.397084	1.711520
H	3.543893	-4.564385	0.212275
C	0.130519	-1.962668	4.318112
H	-0.640178	-2.671204	4.645868
H	0.944568	-2.002902	5.048518
H	-0.310342	-0.960585	4.347435
C	-1.858277	-2.846695	-3.980481
C	-1.879582	-3.631826	-2.668005
H	-2.750009	-3.075166	-4.574815
H	-0.991510	-3.169276	-4.577105
H	-2.804579	-3.406855	-2.120977
H	-1.877711	-4.707756	-2.869985
H	-2.674881	1.663615	-4.971473
O	-1.541669	2.701423	-2.750675
C	-2.388641	3.641796	-3.381004
H	-3.428063	3.286165	-3.370760
H	-2.086964	3.815875	-4.422468
C	-2.269849	4.934301	-2.609242
H	-2.856028	5.711966	-3.122703
H	-1.219260	5.262975	-2.585085

O	-2.751801	4.730772	-1.298512
C	-2.683287	5.898901	-0.511609
H	-3.107992	5.658334	0.466344
H	-1.645012	6.237127	-0.379046
H	-3.263639	6.719491	-0.959538
H	-1.652534	0.594638	0.061820
C	-5.586859	-1.582409	1.600376
C	-6.945626	-1.865833	1.657225
C	-7.675320	-1.812125	0.479004
C	-7.072991	-1.505807	-0.731832
C	-5.711475	-1.228431	-0.743860
C	-4.920903	-1.236913	0.415881
H	-7.418356	-2.124145	2.597794
H	-7.646495	-1.485470	-1.651097
Cl	-4.996630	-0.896453	-2.303023
Cl	-9.384296	-2.153502	0.516078
Cl	-4.703420	-1.716415	3.106267
C	-3.446503	-0.891290	0.389179
H	-3.043819	-1.068968	-0.609606
H	-2.881337	-1.523827	1.078186
N	-2.981467	0.865939	1.972903
C	-3.210563	0.572560	0.708417
C	-3.743294	1.630087	-0.202376
H	-3.557799	1.353914	-1.239718
H	-4.827934	1.694403	-0.043040
H	-3.293866	2.600080	-0.000350
O	-2.594822	2.135736	2.288320
C	-3.372367	2.741353	3.380718
C	-4.827264	2.871780	2.953159
H	-4.912200	3.473938	2.042873
H	-5.280369	1.890305	2.775277
H	-5.398351	3.363830	3.747280
C	-2.698244	4.097384	3.523396
H	-3.185499	4.667482	4.320140
H	-1.638937	3.981769	3.776271
H	-2.777848	4.666704	2.591801
C	-3.212215	1.899596	4.640832
H	-3.727574	0.936695	4.555826
H	-2.152317	1.720415	4.853475
H	-3.649521	2.427807	5.493714
H	-2.616623	0.159971	2.608539

TSB5-6uS

	Coordinates (Angstroms)		
	X	Y	Z
Ir	-0.318576	-0.391417	-0.378037
C	-1.146721	-1.758923	0.887437
N	-0.052333	0.504270	1.478512
C	-1.693149	-3.027516	0.617399
C	-2.351096	-3.755458	1.608144
C	-2.441939	-3.278650	2.918218
C	-1.886383	-2.055334	3.256018
C	-1.250699	-1.328461	2.231501
C	-0.641472	0.518495	3.894027
C	-0.612376	-0.058031	2.508684
C	0.744586	1.677655	1.606053
C	0.444242	2.808805	0.854617
C	1.288182	3.920036	0.877418
C	2.426394	3.870308	1.689804
C	2.747311	2.740708	2.451911
C	1.896787	1.639914	2.391264
O	3.303818	4.936038	1.711902
C	2.917058	5.974248	2.605740
H	-2.964354	-3.869735	3.666560
H	-0.602520	1.611398	3.849379
H	-0.432646	2.808250	0.213083
H	2.151186	0.728822	2.926248
H	3.713645	6.721235	2.582623
H	1.974652	6.441062	2.293538
H	2.805132	5.592241	3.628363
C	0.280216	-1.526471	-2.192977
C	-0.377009	-0.325408	-2.580780
C	0.448061	0.784570	-2.185840
C	1.586938	0.258765	-1.517077
C	1.507108	-1.179842	-1.528937
C	2.577404	-2.161682	-1.137891
C	2.714372	1.084761	-0.958350
C	3.282175	-1.834594	0.157635
C	4.024637	0.695904	-1.595425
C	4.425039	1.335818	-2.799484
C	5.580971	0.991201	-3.442785
C	6.402727	-0.041003	-2.922141
C	6.017204	-0.705687	-1.722912
C	4.809840	-0.308484	-1.064446
C	2.801292	-2.418444	1.371786

C	3.398292	-2.137166	2.575059
C	4.520449	-1.268258	2.628762
C	5.011553	-0.672107	1.436189
C	4.360783	-0.978649	0.195229
C	5.174040	-0.992771	3.859178
C	6.269766	-0.166467	3.904286
C	6.749960	0.439242	2.721367
C	6.129256	0.199949	1.519413
H	-0.073674	-2.528114	-2.395922
H	-1.313110	-0.264683	-3.121215
H	0.246026	1.834426	-2.354553
H	2.123269	-3.151929	-1.070687
H	3.314636	-2.203165	-1.949200
H	2.495124	2.137883	-1.138106
H	2.780995	0.948759	0.121732
H	3.043980	-2.579754	3.499685
H	4.794999	-1.456870	4.766607
H	6.766837	0.028074	4.850660
H	7.608240	1.103818	2.764910
H	6.500662	0.677494	0.618170
O	3.597622	2.306325	-3.324982
C	3.973001	3.632118	-2.962097
H	4.955994	3.887855	-3.376256
H	3.216603	4.299028	-3.382026
H	4.000791	3.754925	-1.871346
O	1.751919	-3.264071	1.222327
C	1.253717	-3.916273	2.376951
H	0.860461	-3.195316	3.102560
H	0.445850	-4.561789	2.032204
H	2.031553	-4.529263	2.848584
H	0.260308	0.189329	4.428616
H	5.861431	1.499741	-4.361424
C	7.599379	-0.432599	-3.579738
C	8.378629	-1.442277	-3.075589
C	6.842101	-1.754821	-1.232729
C	7.992534	-2.111830	-1.890522
H	9.292099	-1.734187	-3.585986
H	7.885363	0.083091	-4.493170
H	8.611436	-2.915425	-1.501395
H	6.551576	-2.278440	-0.327007
C	4.006479	2.715374	3.273156
H	4.890143	2.869838	2.645245
H	4.006382	3.510123	4.028380
H	4.115740	1.756412	3.786787

C	1.008649	5.116018	0.006748
H	0.314011	5.815768	0.486863
H	1.929742	5.662541	-0.215429
H	0.559889	4.800270	-0.939979
C	-1.946913	-1.494096	4.657212
C	-1.880659	0.032731	4.651165
H	-2.860348	-1.836921	5.155613
H	-1.104082	-1.885952	5.245604
H	-2.783774	0.440062	4.177975
H	-1.850772	0.416148	5.675965
H	-2.820179	-4.704286	1.364936
O	-1.594924	-3.497768	-0.667575
C	-1.496410	-4.899909	-0.887106
H	-2.431305	-5.262683	-1.330174
H	-1.325835	-5.437259	0.050872
C	-0.324007	-5.177766	-1.797714
H	-0.208609	-6.266621	-1.916844
H	0.594074	-4.785411	-1.334114
O	-0.545855	-4.564140	-3.049573
C	0.566554	-4.685188	-3.910339
H	0.318251	-4.162343	-4.837467
H	1.466035	-4.228219	-3.470984
H	0.783085	-5.739426	-4.136923
H	-1.835540	0.202711	-0.267869
C	-6.166370	-0.748632	0.616002
C	-7.535632	-0.629895	0.820519
C	-8.312900	-0.102129	-0.198958
C	-7.754114	0.282381	-1.408096
C	-6.381320	0.144656	-1.571964
C	-5.531798	-0.351523	-0.571932
H	-7.982928	-0.948827	1.754546
H	-8.370120	0.674030	-2.209074
Cl	-5.746245	0.590314	-3.141163
Cl	-10.031775	0.064654	0.038081
Cl	-5.246369	-1.467830	1.915289
C	-4.029781	-0.452897	-0.755058
H	-3.778934	-0.719031	-1.784328
H	-3.627958	-1.241332	-0.117783
N	-3.232396	1.743208	-1.336516
C	-3.341630	0.847644	-0.371533
C	-3.360822	1.307814	1.050577
H	-4.369358	1.672602	1.283026
H	-3.134105	0.467159	1.705591
H	-2.645937	2.112117	1.221204

O	-2.602925	2.926944	-1.080034
C	-3.442824	4.106959	-1.364918
C	-3.714006	4.170632	-2.862679
H	-2.776717	4.099555	-3.424808
H	-4.387053	3.371005	-3.189700
H	-4.193667	5.123186	-3.109215
C	-2.545802	5.247299	-0.911136
H	-3.073119	6.198538	-1.031065
H	-2.277848	5.130644	0.144280
H	-1.631414	5.284244	-1.510706
C	-4.727681	4.042648	-0.550630
H	-5.325333	3.161681	-0.809444
H	-4.514273	4.027039	0.522781
H	-5.332488	4.929358	-0.766547
H	-3.166378	1.448878	-2.307740

TSB5-1fR

	Coordinates (Angstroms)		
	X	Y	Z
Ir	0.04131000	-0.77246200	-0.20643800
C	0.44676700	-1.43334700	-2.08050900
N	-0.07390400	0.98487100	-1.32746900
C	0.72039500	-2.74365000	-2.52032600
C	1.13557000	-2.98933300	-3.82770400
C	1.25138500	-1.94982500	-4.75687400
C	0.94640700	-0.64923400	-4.39385700
C	0.54614900	-0.42326800	-3.06172100
C	0.30353300	2.07587500	-3.53782400
C	0.23246300	0.91278600	-2.59071100
C	-0.45785100	2.19831300	-0.68766200
C	0.14090100	2.53315300	0.52452500
C	-0.24476500	3.66720800	1.23252600
C	-1.26340800	4.46124400	0.69463900
C	-1.90299700	4.13227000	-0.50545900
C	-1.49400600	2.98627800	-1.18754200
O	-1.70813600	5.56954400	1.38726700
C	-0.93740300	6.74324600	1.15471300
H	1.58168800	-2.17308700	-5.76854800
H	0.52277000	3.00131400	-2.99708700
H	0.88158000	1.86949400	0.95454800
H	-2.01246900	2.68935700	-2.09421400

H	-1.43674000	7.55643100	1.68662700
H	0.08435300	6.63503800	1.53878100
H	-0.89303800	6.98261400	0.08489000
C	-0.85043700	-2.54271900	0.76554300
C	0.09782000	-1.96207200	1.65069700
C	-0.40093700	-0.66599500	2.04651600
C	-1.62154600	-0.44455200	1.36747400
C	-1.91690500	-1.60270200	0.55206500
C	-3.17689900	-1.90722000	-0.21833000
C	-2.49509000	0.77183800	1.51831500
C	-3.69984700	-0.74906400	-1.03945100
C	-3.89564000	0.37145000	1.91405300
C	-4.19958600	0.17473700	3.29579200
C	-5.45685800	-0.20131500	3.69541600
C	-6.46938600	-0.43492900	2.72588400
C	-6.17639500	-0.28493200	1.34281700
C	-4.85811400	0.13072100	0.96127400
C	-3.36437000	-0.67477400	-2.42891200
C	-3.79310600	0.37192600	-3.20660500
C	-4.59831400	1.39679700	-2.64328200
C	-4.95402500	1.33885000	-1.26940500
C	-4.49261600	0.23182800	-0.48459800
C	-5.07126200	2.47727000	-3.43428800
C	-5.86864500	3.45260700	-2.88754300
C	-6.21198800	3.40330200	-1.51729500
C	-5.75788000	2.37576900	-0.72670200
H	-0.79506300	-3.51516000	0.30216800
H	1.00562500	-2.42803900	2.01017500
H	0.06707300	0.02070800	2.73648800
H	-2.97065000	-2.75815800	-0.87112000
H	-3.94980100	-2.22651500	0.49148500
H	-2.05656100	1.43534500	2.26613500
H	-2.53580200	1.32663000	0.57948200
H	-3.55004000	0.42915300	-4.26202300
H	-4.79904000	2.51328500	-4.48667400
H	-6.23106200	4.26966500	-3.50555200
H	-6.83102700	4.18588700	-1.08781400
H	-6.01673000	2.35043000	0.32756600
O	-3.15101000	0.37957200	4.13257600
C	-3.35276100	0.18576000	5.52133500
H	-3.65734700	-0.84549100	5.73637800
H	-2.38793300	0.38078600	5.99195300
H	-4.10093200	0.88398000	5.91563700
O	-2.63467400	-1.71606500	-2.89574500

C	-2.38793100	-1.78906500	-4.28812000
H	-1.79665800	-0.93532200	-4.63617200
H	-1.82161600	-2.70801700	-4.44223500
H	-3.32929200	-1.84049100	-4.84937900
H	-0.68740600	2.20557800	-3.99573000
H	-5.69975500	-0.34484600	4.74300900
C	-7.77370100	-0.84211000	3.11316300
C	-8.74053900	-1.09746400	2.17254400
C	-7.19320800	-0.56915000	0.39325800
C	-8.44567000	-0.96329300	0.79696800
H	-9.73416700	-1.40926900	2.48314000
H	-7.99205500	-0.95408300	4.17256100
H	-9.21230300	-1.17592800	0.05716200
H	-6.97469800	-0.47519600	-0.66600700
H	1.68968300	-0.45866600	-0.24191200
C	5.95793100	1.17454000	0.98017300
C	7.20699900	1.73574300	0.74290400
C	7.32462800	2.67457700	-0.27032100
C	6.22919800	3.06100100	-1.02770700
C	4.99995000	2.47181100	-0.75758900
C	4.81275900	1.50324700	0.24072300
H	8.06247200	1.44994300	1.34409800
H	6.32342000	3.80794300	-1.80724500
Cl	3.64919000	3.03078700	-1.72327400
Cl	8.88078700	3.39698800	-0.58896300
Cl	5.85102200	0.03575300	2.29791300
C	3.45925200	0.88392000	0.51435400
H	2.69348600	1.54658100	0.11625800
H	3.27405200	0.79792800	1.58963500
N	3.51973000	-1.52290400	0.61418600
C	3.26978400	-0.46903400	-0.15696200
C	3.52898800	-0.56813500	-1.62976100
H	3.03388700	0.25073900	-2.15210200
H	4.61103600	-0.47812600	-1.78756000
H	3.18637200	-1.51700100	-2.03842200
O	3.18133500	-2.74653400	0.08619700
C	4.24469500	-3.74545500	0.17655100
C	4.54647100	-4.04130600	1.64141800
H	5.24209600	-4.88425600	1.71200600
H	5.01062700	-3.18449900	2.14059800
H	3.62872600	-4.30268600	2.17845600
C	3.61148400	-4.94359800	-0.51660400
H	3.36307000	-4.69683200	-1.55392400
H	4.31483100	-5.78213500	-0.51873200

H	2.69970300	-5.25484900	0.00314900
C	5.48064700	-3.26264600	-0.57269500
H	6.26135900	-4.02921100	-0.52395900
H	5.25079600	-3.07490800	-1.62659700
H	5.88223700	-2.34564900	-0.12886500
H	3.34505900	-1.46914600	1.63279700
S	2.45370100	-0.61440700	4.26695400
C	3.61108100	-0.33365100	5.59229000
H	3.79150800	-1.27727200	6.11274300
H	4.54607000	0.05170000	5.17988900
H	3.18309400	0.39642000	6.28387700
O	1.21091500	-1.10718200	4.89450000
O	3.09242900	-1.64072600	3.38912500
O	2.29135500	0.69023000	3.57832900
C	-3.02687000	4.99030500	-1.01769700
H	-3.45340300	4.56929700	-1.93198800
H	-3.82509200	5.07987300	-0.27325700
H	-2.68129600	6.00675300	-1.24122500
C	0.38885500	3.99359400	2.55807300
H	-0.34895200	4.39718300	3.25888400
H	0.83922000	3.10001500	3.00186900
H	1.18226000	4.74389800	2.45119800
C	1.02617700	0.50402600	-5.36496300
H	0.06753400	0.60346600	-5.89631400
H	1.78553200	0.29975800	-6.12790300
C	1.32988100	1.81581600	-4.64375900
H	2.33664100	1.77040600	-4.20921200
H	1.31803500	2.65144300	-5.35103000
H	1.38711800	-3.99893500	-4.13863800
O	0.60335400	-3.75343700	-1.60528400
C	0.35092300	-5.06823000	-2.07404600
H	-0.28431700	-5.04070400	-2.96839600
H	1.28647200	-5.58366400	-2.32584200
C	-0.36667400	-5.82091800	-0.97988300
H	-0.43283800	-6.88523600	-1.25373900
H	0.19233200	-5.74699000	-0.03422300
O	-1.65578400	-5.26181400	-0.83523100
C	-2.37771700	-5.81438000	0.24306500
H	-1.85095900	-5.66863800	1.19764300
H	-3.33998200	-5.29839200	0.28732200
H	-2.55339500	-6.89066100	0.09794100

TSB5f-1S

	Coordinates (Angstroms) X	Y	Z
Ir	-0.095253	0.257050	-0.155095
C	-0.886756	-0.208831	-1.967721
N	0.300116	-1.779775	-0.299963
C	-1.524105	0.634215	-2.900082
C	-2.102850	0.111656	-4.056594
C	-2.047859	-1.258418	-4.338199
C	-1.418742	-2.129635	-3.466773
C	-0.854002	-1.580709	-2.298217
C	-0.033637	-3.892704	-1.581516
C	-0.161938	-2.415947	-1.338541
C	1.098635	-2.421826	0.688716
C	0.785833	-2.267793	2.036009
C	1.605110	-2.808131	3.026541
C	2.747261	-3.512978	2.630707
C	3.094017	-3.661361	1.282693
C	2.261470	-3.098072	0.318948
O	3.606262	-4.023662	3.583465
C	3.230370	-5.304287	4.076233
H	-2.513249	-1.633791	-5.246426
H	0.036002	-4.432127	-0.632055
H	-0.074941	-1.671239	2.321891
H	2.543321	-3.151056	-0.728381
H	4.000056	-5.600503	4.792448
H	2.258599	-5.269021	4.584215
H	3.184193	-6.044031	3.267083
C	0.466383	2.385238	-0.328632
C	-0.175591	2.181357	0.923620
C	0.665105	1.334083	1.727054
C	1.802448	0.992791	0.953563
C	1.691254	1.629694	-0.337369
C	2.735493	1.727959	-1.418119
C	2.968262	0.171434	1.433614
C	3.493775	0.447698	-1.691585
C	4.240303	0.977511	1.339078
C	4.605867	1.825040	2.429070
C	5.744139	2.587944	2.379039
C	6.559233	2.570063	1.214898
C	6.194934	1.759752	0.104935
C	5.015116	0.950964	0.201984
C	3.067931	-0.399067	-2.764123

C	3.738320	-1.560392	-3.059351
C	4.870783	-1.945738	-2.294106
C	5.300389	-1.131666	-1.212145
C	4.589954	0.084175	-0.939736
C	5.592028	-3.131760	-2.595818
C	6.690104	-3.495113	-1.855472
C	7.105372	-2.697920	-0.765078
C	6.422060	-1.549207	-0.447743
H	0.114071	2.999097	-1.145566
H	-1.112741	2.618036	1.239682
H	0.477505	1.015165	2.742975
H	2.242071	2.068744	-2.331105
H	3.448823	2.510387	-1.130565
H	2.786974	-0.139159	2.463824
H	3.067866	-0.733967	0.834079
H	3.433389	-2.195720	-3.883816
H	5.261803	-3.746345	-3.430105
H	7.236636	-4.401945	-2.100157
H	7.965112	-2.998684	-0.172929
H	6.744649	-0.949525	0.397627
O	3.740682	1.795243	3.475868
C	4.054869	2.559857	4.626654
H	4.083100	3.631828	4.397799
H	3.253147	2.364095	5.340614
H	5.012576	2.248682	5.060855
O	1.994917	0.055031	-3.454030
C	1.571828	-0.666569	-4.596162
H	1.271652	-1.687923	-4.337730
H	0.709778	-0.123849	-4.985092
H	2.362397	-0.695174	-5.356308
H	0.906674	-4.072935	-2.120709
H	6.036724	3.226558	3.205851
C	7.732563	3.365504	1.131194
C	8.503414	3.365921	-0.004858
C	7.005468	1.790626	-1.060532
C	8.132942	2.573299	-1.114890
H	9.399144	3.978985	-0.055510
H	8.009244	3.980235	1.984604
H	8.741012	2.586519	-2.015131
H	6.723832	1.189420	-1.919891
H	-1.649837	-0.186280	0.244901
C	-5.688386	-1.802764	-0.925085
C	-6.936484	-2.406303	-0.825053
C	-7.896419	-1.805735	-0.025753

C	-7.637896	-0.617706	0.639196
C	-6.376797	-0.047058	0.509305
C	-5.344611	-0.620197	-0.250113
H	-7.148868	-3.323712	-1.361519
H	-8.398881	-0.137229	1.243121
Cl	-6.133816	1.474643	1.325692
Cl	-9.466020	-2.551582	0.136087
Cl	-4.537633	-2.586061	-1.980846
C	-3.952963	-0.019351	-0.327906
H	-3.994387	1.072008	-0.402904
H	-3.450084	-0.376181	-1.227468
N	-3.125263	0.420449	1.882467
C	-3.104412	-0.431935	0.867009
C	-2.899229	-1.895183	1.125632
H	-3.864335	-2.339006	1.400430
H	-2.538426	-2.384228	0.219060
H	-2.194616	-2.067479	1.936162
O	-2.274163	0.157733	2.927717
C	-2.928756	0.223257	4.236493
C	-4.031241	-0.826267	4.311033
H	-4.510267	-0.787228	5.294832
H	-4.803180	-0.645803	3.555347
H	-3.622480	-1.832073	4.167425
C	-1.784401	-0.101599	5.185219
H	-0.986743	0.644552	5.103574
H	-2.149113	-0.101443	6.217276
H	-1.370864	-1.091874	4.967908
C	-3.461867	1.632201	4.470112
H	-3.812623	1.727258	5.503085
H	-2.674258	2.374031	4.300710
H	-4.304407	1.862069	3.810124
H	-3.230768	1.433965	1.696376
S	-3.331484	4.051630	0.348608
C	-4.581527	5.283301	0.664680
H	-4.309551	5.847595	1.560181
H	-4.635461	5.954420	-0.196312
H	-5.545111	4.789739	0.810600
O	-3.778627	3.301303	-0.845784
O	-3.295318	3.205208	1.579778
O	-2.066746	4.793020	0.148760
C	4.357892	-4.380166	0.899621
H	5.229263	-3.938739	1.394271
H	4.321365	-5.436208	1.193244
H	4.516894	-4.336301	-0.181272

C	1.286323	-2.589949	4.481750
H	0.546262	-3.314053	4.844413
H	2.182088	-2.690728	5.100500
H	0.872054	-1.588604	4.636433
C	-1.313037	-3.612948	-3.731637
H	-0.423065	-3.813044	-4.347538
H	-2.175991	-3.952209	-4.315722
C	-1.197843	-4.407509	-2.431223
H	-2.131314	-4.326313	-1.860380
H	-1.042771	-5.469584	-2.646772
H	-2.619692	0.763553	-4.753552
O	-1.572430	1.963597	-2.597595
C	-1.840431	2.884976	-3.640364
H	-1.292272	2.598596	-4.547896
H	-2.913428	2.915100	-3.870673
C	-1.387379	4.249885	-3.184330
H	-1.678451	4.992509	-3.944208
H	-1.873075	4.511753	-2.236698
O	0.018394	4.226731	-3.026739
C	0.516560	5.405263	-2.431366
H	0.052369	5.583675	-1.451374
H	1.593142	5.269308	-2.298012
H	0.341029	6.283940	-3.070500

TSB7-8uR

	Coordinates (Angstroms) X	Y	Z
Ir	-0.282589	-0.799341	0.597198
C	-1.104714	-0.158068	2.320100
N	-0.063615	1.278561	0.444694
C	-1.734386	-0.935118	3.306189
C	-2.266513	-0.346045	4.449311
C	-2.241808	1.045320	4.609321
C	-1.679751	1.861961	3.640686
C	-1.105795	1.234751	2.517237
C	-0.453990	3.495210	1.536858
C	-0.502372	1.998241	1.438092
C	0.547451	1.821629	-0.720866
C	0.143342	1.327983	-1.960177
C	0.733617	1.763206	-3.141689
C	1.756158	2.714156	-3.051940

C	2.201118	3.206544	-1.820893
C	1.592405	2.741696	-0.654076
O	2.394721	3.148277	-4.196029
C	1.710698	4.199182	-4.870865
H	-2.685381	1.484080	5.499800
H	-0.438103	3.955649	0.545049
H	-0.617810	0.556255	-1.998086
H	1.959834	3.080687	0.309133
H	2.350645	4.508015	-5.700504
H	0.745579	3.860028	-5.267019
H	1.542378	5.053010	-4.202959
C	0.672591	-2.637377	1.319727
C	-0.097661	-2.950187	0.167542
C	0.530798	-2.307468	-0.968451
C	1.631795	-1.565863	-0.498154
C	1.728023	-1.742969	0.939069
C	2.862297	-1.354169	1.847399
C	2.593347	-0.768966	-1.337945
C	3.432619	0.029914	1.644996
C	3.991435	-1.315338	-1.171017
C	4.402398	-2.407279	-1.984005
C	5.641082	-2.971023	-1.853534
C	6.539948	-2.492016	-0.867255
C	6.141009	-1.420344	-0.018793
C	4.847104	-0.830703	-0.200480
C	2.999068	1.087270	2.504890
C	3.575964	2.330948	2.442450
C	4.587599	2.599585	1.482957
C	4.982463	1.584931	0.569952
C	4.398277	0.280808	0.694459
C	5.217763	3.870797	1.418431
C	6.188073	4.128227	0.481971
C	6.558011	3.130865	-0.448323
C	5.964826	1.892541	-0.408145
H	0.492733	-2.995480	2.323919
H	-0.938390	-3.627362	0.143591
H	0.207650	-2.364576	-1.999173
H	2.517088	-1.456064	2.878705
H	3.664653	-2.090330	1.710173
H	2.278732	-0.818169	-2.382125
H	2.576362	0.280668	-1.040502
H	3.283407	3.124134	3.121865
H	4.917999	4.638335	2.128005
H	6.666598	5.102898	0.446030

H	7.312757	3.346743	-1.199126
H	6.253991	1.134237	-1.128944
O	3.493794	-2.914954	-2.887261
C	3.708494	-2.496076	-4.231371
H	4.678484	-2.846385	-4.605248
H	2.907840	-2.941013	-4.826188
H	3.663047	-1.402757	-4.317622
O	2.023576	0.743677	3.381703
C	1.661047	1.680410	4.380704
H	1.249821	2.594937	3.939804
H	0.894454	1.191831	4.983179
H	2.522475	1.931433	5.011989
H	0.494759	3.767395	2.021469
H	5.927321	-3.803556	-2.490891
C	7.821813	-3.079201	-0.693037
C	8.670560	-2.633244	0.287410
C	7.039558	-0.992984	0.997671
C	8.270333	-1.581944	1.145152
H	9.647852	-3.090772	0.413767
H	8.115319	-3.893745	-1.350600
H	8.942758	-1.241553	1.927678
H	6.744247	-0.194286	1.670601
C	3.334409	4.193491	-1.772830
H	4.238360	3.779225	-2.231376
H	3.088030	5.111202	-2.320012
H	3.568557	4.465542	-0.740903
C	0.301954	1.194849	-4.466552
H	-0.488346	1.803215	-4.923722
H	1.136577	1.156473	-5.173127
H	-0.095514	0.182096	-4.341106
C	-1.658273	3.366812	3.755630
C	-1.617743	4.028695	2.378591
H	-2.533401	3.711428	4.317579
H	-0.774761	3.677988	4.332422
H	-2.563749	3.841521	1.854732
H	-1.513966	5.113488	2.483097
H	-2.724799	-0.955259	5.222394
O	-1.826794	-2.275005	3.040833
C	-2.742349	-3.077598	3.763638
H	-3.681450	-2.536210	3.935145
H	-2.326145	-3.363870	4.738506
C	-2.983870	-4.317987	2.925962
H	-3.562175	-5.047970	3.507392
H	-2.025069	-4.771709	2.658602

O	-3.647507	-4.032694	1.702884
C	-5.055098	-3.922218	1.826961
H	-5.450372	-3.698064	0.833001
H	-5.488210	-4.867880	2.178782
H	-5.347340	-3.118185	2.513929
H	-1.899811	-0.586204	0.052417
C	-6.058486	0.636683	-1.403113
C	-7.079424	1.559181	-1.216074
C	-6.733696	2.881538	-0.980244
C	-5.407857	3.284746	-0.946096
C	-4.420566	2.325881	-1.141091
C	-4.696018	0.969056	-1.361337
H	-8.117452	1.250071	-1.255652
H	-5.143368	4.321673	-0.774899
Cl	-2.771591	2.895575	-1.128675
Cl	-7.992298	4.063184	-0.731526
Cl	-6.541467	-1.015191	-1.705424
C	-3.608993	-0.064600	-1.555695
H	-2.674171	0.431565	-1.807481
H	-3.853609	-0.703318	-2.408290
N	-3.319861	-2.284958	-0.591420
C	-3.402835	-0.976193	-0.340899
C	-4.051051	-0.627766	0.968970
H	-3.812977	0.400934	1.246026
H	-5.138318	-0.720121	0.872995
H	-3.709786	-1.301412	1.752827
O	-2.577030	-2.620486	-1.700167
C	-3.020313	-3.831254	-2.396527
C	-4.489608	-3.715491	-2.773849
H	-4.781637	-4.586049	-3.370247
H	-5.128001	-3.686737	-1.885515
H	-4.667662	-2.815410	-3.370183
C	-2.128061	-3.823110	-3.630902
H	-1.071005	-3.884194	-3.351065
H	-2.364369	-4.685414	-4.262077
H	-2.286857	-2.910666	-4.215517
C	-2.753506	-5.061356	-1.533604
H	-3.080731	-5.957690	-2.070978
H	-1.684618	-5.168824	-1.322463
H	-3.299801	-5.028600	-0.584810
H	-3.180796	-2.912982	0.211611

TSB7f-1R

	Coordinates (Angstroms) X	Y	Z
Ir	-0.259968	0.518444	-0.253210
C	-1.031005	0.281610	-2.120282
N	0.028379	-1.526752	-0.603821
C	-1.579945	1.252714	-2.979315
C	-2.155008	0.890542	-4.197157
C	-2.149489	-0.437474	-4.630875
C	-1.565174	-1.425138	-3.856636
C	-1.027477	-1.041952	-2.611453
C	-0.248221	-3.433841	-2.198444
C	-0.390804	-2.010317	-1.738777
C	0.693405	-2.310937	0.379719
C	0.286107	-2.198965	1.707713
C	0.951192	-2.877006	2.725136
C	2.045139	-3.677894	2.377346
C	2.487685	-3.790933	1.055482
C	1.806793	-3.087141	0.062044
O	2.760580	-4.325287	3.364179
C	2.215549	-5.582882	3.746760
H	-2.599791	-0.687485	-5.588415
H	-0.189343	-4.118629	-1.348029
H	-0.526431	-1.525959	1.962393
H	2.174903	-3.116126	-0.958700
H	2.895092	-6.002978	4.491827
H	1.218271	-5.470484	4.189754
H	2.151167	-6.264553	2.889460
C	0.530317	2.568223	-0.379643
C	-0.211596	2.437079	0.821594
C	0.498488	1.513687	1.681021
C	1.630803	1.046803	0.981210
C	1.658227	1.678926	-0.322294
C	2.774905	1.682476	-1.330210
C	2.654974	0.081506	1.513378
C	3.415468	0.339208	-1.590058
C	4.023363	0.717728	1.514835
C	4.437736	1.470429	2.647933
C	5.643567	2.113475	2.678931
C	6.505801	2.062231	1.553827
C	6.111000	1.321587	0.403746
C	4.851936	0.638245	0.412860
C	2.990822	-0.423344	-2.723971

C	3.586121	-1.620480	-3.035702
C	4.628279	-2.132828	-2.217563
C	5.037101	-1.412945	-1.062597
C	4.415764	-0.150984	-0.780493
C	5.280533	-3.353339	-2.537617
C	6.291677	-3.839802	-1.745896
C	6.680683	-3.138781	-0.582498
C	6.062108	-1.959410	-0.245988
H	0.289236	3.201618	-1.220986
H	-1.101330	2.999206	1.066293
H	0.218208	1.217482	2.681555
H	2.379664	2.090652	-2.263398
H	3.542965	2.381908	-0.976970
H	2.361230	-0.221494	2.519304
H	2.676767	-0.818812	0.897854
H	3.291323	-2.186886	-3.912620
H	4.969115	-3.894073	-3.428281
H	6.787788	-4.770753	-2.006213
H	7.467153	-3.538630	0.051224
H	6.362457	-1.433706	0.654949
O	3.577753	1.558365	3.723242
C	3.819080	0.573366	4.723826
H	4.802560	0.717817	5.188475
H	3.038444	0.693127	5.478405
H	3.766851	-0.441021	4.307095
O	2.002418	0.149878	-3.450953
C	1.615191	-0.460605	-4.667763
H	1.240434	-1.477063	-4.507315
H	0.812592	0.160390	-5.067501
H	2.451230	-0.484669	-5.378064
H	0.713962	-3.513851	-2.725604
H	5.932584	2.677505	3.561768
C	7.753654	2.741081	1.549416
C	8.571696	2.697989	0.449367
C	6.976639	1.307862	-0.724466
C	8.174540	1.977135	-0.701182
H	9.523932	3.220836	0.455093
H	8.046859	3.299487	2.435097
H	8.822913	1.956674	-1.572733
H	6.680549	0.764746	-1.616603
H	-1.853534	0.093292	0.229120
C	-5.906660	-1.595973	0.985110
C	-6.952459	-2.306703	0.410433
C	-6.650483	-3.459231	-0.299607

C	-5.348827	-3.926007	-0.396915
C	-4.336094	-3.180309	0.195154
C	-4.558769	-1.964959	0.857716
H	-7.976494	-1.970474	0.524482
H	-5.124072	-4.852978	-0.911445
Cl	-2.732426	-3.874469	0.150789
Cl	-7.940575	-4.360571	-1.053395
Cl	-6.344177	-0.204099	1.948379
C	-3.429370	-1.151392	1.441175
H	-2.500553	-1.706387	1.341460
H	-3.591837	-1.017171	2.514048
N	-3.164189	1.231365	1.727148
C	-3.253270	0.249502	0.829478
C	-3.948482	0.636152	-0.452233
H	-3.818509	-0.150501	-1.198003
H	-5.018078	0.782544	-0.274082
H	-3.535560	1.567578	-0.844458
O	-2.372775	0.949705	2.817856
C	-2.665764	1.743680	4.011454
C	-4.163901	1.771041	4.282558
H	-4.347137	2.281336	5.233923
H	-4.702223	2.312981	3.498810
H	-4.566696	0.755687	4.356091
C	-1.915494	0.980223	5.096074
H	-0.848291	0.911073	4.857816
H	-2.015685	1.503418	6.052260
H	-2.319184	-0.031275	5.212073
C	-2.096695	3.150223	3.839171
H	-2.259878	3.722361	4.758975
H	-1.019264	3.114772	3.647851
H	-2.584689	3.688787	3.020762
H	-3.177399	2.215592	1.420727
S	-3.518432	4.819281	0.092850
C	-4.447970	6.279773	0.522519
H	-4.240003	6.540153	1.563139
H	-4.142432	7.097803	-0.134651
H	-5.513331	6.075504	0.392579
O	-3.862865	4.499142	-1.309079
O	-3.989424	3.767708	1.042177
O	-2.091944	5.162159	0.298659
C	3.691100	-4.630991	0.727926
H	4.559347	-4.332745	1.324774
H	3.503755	-5.691022	0.938283
H	3.950015	-4.538642	-0.329964

C	0.527978	-2.707841	4.159546
H	-0.163529	-3.500005	4.471933
H	1.388739	-2.738427	4.834859
H	0.014015	-1.750860	4.296469
C	-1.459536	-2.853525	-4.330606
H	-0.560332	-2.958845	-4.956121
H	-2.313466	-3.100241	-4.971666
C	-1.371388	-3.828980	-3.161342
H	-2.328405	-3.843075	-2.628031
H	-1.188710	-4.845147	-3.526031
H	-2.623685	1.641651	-4.825073
O	-1.552583	2.553094	-2.560763
C	-1.637165	3.577356	-3.542405
H	-1.119521	3.266889	-4.458612
H	-2.685033	3.794508	-3.782426
C	-0.974811	4.815214	-2.989352
H	-1.181431	5.663761	-3.660256
H	-1.382208	5.047197	-1.998461
O	0.419393	4.579343	-2.905373
C	1.093884	5.594689	-2.194306
H	0.680090	5.717767	-1.182652
H	2.143340	5.297136	-2.119652
H	1.030530	6.560233	-2.718251

TSB7f-1S

	Coordinates (Angstroms)		
	X	Y	Z
Ir	0.150988	-0.671086	-0.125618
C	0.867235	-0.747749	-2.030875
N	0.079587	1.333650	-0.712149
C	1.198694	-1.859475	-2.830681
C	1.798201	-1.698858	-4.078128
C	2.058306	-0.427643	-4.598817
C	1.689552	0.702735	-3.891421
C	1.083941	0.512401	-2.633176
C	0.742205	3.033220	-2.412170
C	0.601852	1.642859	-1.864123
C	-0.530224	2.299199	0.136881
C	-0.163977	2.343854	1.478267
C	-0.787191	3.215529	2.366875
C	-1.800680	4.043980	1.872856

C	-2.205681	4.000397	0.534044
C	-1.563075	3.112383	-0.327778
O	-2.472726	4.893344	2.728889
C	-1.809821	6.135397	2.939750
H	2.542789	-0.334613	-5.567660
H	0.775090	3.766836	-1.602166
H	0.587305	1.654495	1.845168
H	-1.894542	3.029728	-1.358634
H	-2.460283	6.734981	3.580643
H	-0.842452	5.995418	3.437471
H	-1.649848	6.662455	1.990696
C	-0.799785	-2.645029	0.106909
C	-0.042389	-2.361643	1.273646
C	-0.667509	-1.250375	1.949968
C	-1.771260	-0.832483	1.173336
C	-1.860650	-1.680423	0.002652
C	-2.973850	-1.755008	-1.008019
C	-2.726749	0.271217	1.538091
C	-3.494511	-0.419343	-1.488676
C	-4.134580	-0.268313	1.615119
C	-4.592415	-0.839356	2.834017
C	-5.842649	-1.382039	2.945609
C	-6.708108	-1.410549	1.822520
C	-6.264565	-0.865273	0.584489
C	-4.958191	-0.283986	0.506824
C	-2.994788	0.121708	-2.715604
C	-3.468599	1.312258	-3.208692
C	-4.465719	2.032864	-2.499753
C	-4.958725	1.528259	-1.266594
C	-4.454157	0.274960	-0.785038
C	-4.992886	3.249651	-3.008457
C	-5.967763	3.934377	-2.325685
C	-6.446051	3.443270	-1.090016
C	-5.946282	2.273994	-0.569979
H	-0.626431	-3.436332	-0.605979
H	0.799791	-2.933600	1.638499
H	-0.355435	-0.813399	2.888576
H	-2.617546	-2.341126	-1.857767
H	-3.800875	-2.317754	-0.557331
H	-2.421666	0.698444	2.494728
H	-2.682489	1.069174	0.795342
H	-3.109467	1.718485	-4.148111
H	-4.615149	3.627939	-3.955543
H	-6.369430	4.859207	-2.730770

H	-7.207789	3.996166	-0.547479
H	-6.315347	1.909447	0.383680
O	-3.721480	-0.866175	3.901522
C	-3.936998	0.174990	4.848287
H	-4.919086	0.075410	5.327226
H	-3.153559	0.079738	5.603204
H	-3.865085	1.162316	4.373971
O	-2.067393	-0.642848	-3.339021
C	-1.623044	-0.247842	-4.623685
H	-1.123919	0.726650	-4.592816
H	-0.909298	-1.009671	-4.937793
H	-2.457647	-0.212695	-5.335160
H	-0.151575	3.261930	-3.009956
H	-6.164541	-1.811611	3.890466
C	-8.001749	-1.992166	1.902984
C	-8.819848	-2.037298	0.803101
C	-7.133585	-0.937394	-0.538886
C	-8.377812	-1.506252	-0.431430
H	-9.806460	-2.486394	0.875019
H	-8.330625	-2.405797	2.853177
H	-9.028390	-1.553095	-1.300315
H	-6.802260	-0.540470	-1.493460
H	1.768517	-0.334609	0.295599
C	4.574072	2.969419	0.329809
C	5.625470	3.823621	0.640989
C	6.921451	3.366640	0.457851
C	7.174530	2.099045	-0.042397
C	6.092748	1.279247	-0.340861
C	4.757074	1.663295	-0.153340
H	5.432485	4.822502	1.014446
H	8.189058	1.753233	-0.202604
Cl	6.465444	-0.284593	-1.018339
Cl	8.260699	4.415547	0.846304
Cl	2.965989	3.627328	0.550389
C	3.593634	0.750729	-0.480493
H	3.794589	0.172453	-1.382805
H	2.723104	1.369373	-0.684832
N	3.706976	-1.434625	0.653113
C	3.228419	-0.191312	0.663914
C	3.112108	0.387011	2.053184
H	4.113997	0.538277	2.470183
H	2.608342	1.351533	2.013225
H	2.555220	-0.281408	2.712573
O	3.753301	-2.072161	-0.564469

C	4.161619	-3.473358	-0.497754
C	3.010540	-4.298748	0.072663
H	3.222508	-5.366469	-0.051669
H	2.864649	-4.116449	1.142016
H	2.087379	-4.056642	-0.460875
C	4.397104	-3.794082	-1.968169
H	5.241800	-3.220168	-2.363493
H	4.613123	-4.860150	-2.087998
H	3.508549	-3.550629	-2.556546
C	5.441732	-3.646164	0.314380
H	5.785395	-4.681454	0.215907
H	6.233075	-2.985895	-0.052540
H	5.282567	-3.452113	1.379423
H	3.528210	-2.030642	1.476802
S	2.539569	-3.028941	4.030791
C	3.254239	-3.500056	5.592765
H	3.811920	-2.653011	5.999003
H	2.452338	-3.782473	6.279112
H	3.924711	-4.347857	5.432834
O	1.810714	-4.213819	3.525765
O	3.701438	-2.665228	3.166535
O	1.657454	-1.869844	4.301833
C	-3.337206	4.870049	0.060059
H	-4.256044	4.662490	0.618717
H	-3.111146	5.933918	0.198484
H	-3.537572	4.700279	-1.001128
C	-0.400980	3.227209	3.821494
H	0.402360	3.948521	4.016583
H	-1.250819	3.498863	4.455134
H	-0.037594	2.241548	4.130506
C	1.907095	2.096735	-4.430828
H	1.080417	2.364369	-5.105782
H	2.821019	2.120930	-5.035057
C	1.981331	3.126760	-3.305580
H	2.883277	2.952960	-2.704041
H	2.056670	4.137734	-3.718581
H	2.083100	-2.569795	-4.660266
O	0.932990	-3.101713	-2.326617
C	0.776431	-4.184375	-3.227975
H	0.205611	-3.864755	-4.109391
H	1.749347	-4.570173	-3.560042
C	0.029324	-5.279913	-2.506801
H	0.010247	-6.181051	-3.139152
H	0.544898	-5.535202	-1.568595

O	-1.282144	-4.828083	-2.244740
C	-2.016114	-5.712514	-1.426612
H	-1.530862	-5.853104	-0.449342
H	-3.002597	-5.267670	-1.273462
H	-2.136635	-6.696023	-1.904686

E-2a-H$^+$

	Coordinates (Angstroms) X	Y	Z
C	-1.460601	-0.983032	-0.407979
C	-2.690551	-1.521105	-0.057387
C	-3.734715	-0.645472	0.202672
C	-3.570159	0.727979	0.112226
C	-2.321255	1.224443	-0.240850
C	-1.224295	0.394792	-0.508576
H	-2.828974	-2.593864	0.010181
H	-4.394027	1.402618	0.312657
Cl	-2.177847	2.965199	-0.351465
Cl	-5.292367	-1.286821	0.650711
Cl	-0.164010	-2.110009	-0.741899
C	0.136115	0.930448	-0.893441
H	0.082311	2.013927	-1.042418
H	0.459655	0.496740	-1.845748
N	2.330658	0.297158	-0.206263
C	1.172934	0.709038	0.170757
C	0.921378	0.955196	1.610953
H	0.003127	1.524578	1.755668
H	0.820825	-0.009677	2.124476
H	1.767111	1.482061	2.062082
O	3.304904	0.081295	0.704364
C	4.641060	-0.236335	0.138738
C	4.580774	-1.593252	-0.550414
H	4.169148	-2.348179	0.126582
H	3.985006	-1.577669	-1.469948
H	5.595494	-1.893314	-0.830189
C	5.477792	-0.286016	1.407444
H	6.511639	-0.533222	1.150134
H	5.468204	0.683648	1.914652
H	5.098978	-1.049114	2.094630
C	5.086860	0.889903	-0.783344
H	4.516072	0.922456	-1.718504

H	5.000294	1.857303	-0.278991
H	6.135572	0.730934	-1.051836
H	2.545504	0.098517	-1.184210

----

tAmylOH

	Coordinates (Angstroms) X	Y	Z
C	-2.151886	-0.002825	0.089531
H	-2.216302	0.924060	0.670456
H	-3.052760	-0.061054	-0.531709
H	-2.182862	-0.844304	0.790579
C	-0.904407	-0.035706	-0.788412
H	-0.922226	0.817181	-1.479531
H	-0.915959	-0.941339	-1.408965
C	0.436590	-0.000245	-0.041963
C	0.595300	1.276933	0.783809
H	-0.120458	1.314854	1.611671
H	1.602596	1.328517	1.215712
H	0.446698	2.162864	0.155832
C	0.636226	-1.239363	0.832286
H	-0.085781	-1.275983	1.654646
H	0.529211	-2.153000	0.236253
H	1.640261	-1.235104	1.274579
O	1.414537	-0.005282	-1.094712
H	2.290353	0.012801	-0.683332

E-2a-H^{+}-tAmylOH

	Coordinates (Angstroms) X	Y	Z
C	2.219636	0.319289	-0.988001
C	3.538725	0.567459	-1.341112
C	4.539136	0.086046	-0.509005
C	4.245663	-0.626301	0.644034
C	2.911285	-0.851370	0.958808
C	1.854824	-0.391810	0.162303
H	3.777658	1.121272	-2.241652
H	5.034148	-0.997479	1.288365
Cl	2.587893	-1.748330	2.426302

Cl	6.206210	0.388695	-0.921458
Cl	0.975849	0.940798	-2.049943
C	0.405027	-0.635943	0.502609
H	0.324078	-1.420286	1.262657
H	-0.144466	-0.990690	-0.372759
N	-1.440160	0.864604	0.628604
C	-0.273926	0.573561	1.084365
C	0.306755	1.357226	2.201423
H	1.379598	1.184695	2.289099
H	0.110510	2.424172	2.067376
H	-0.177527	1.047070	3.136064
O	-2.166322	1.832077	1.239593
C	-2.974547	2.672299	0.322767
C	-2.103284	3.162449	-0.823804
H	-1.182775	3.620911	-0.448149
H	-1.843668	2.352102	-1.512696
H	-2.654325	3.915776	-1.395184
C	-3.389618	3.806304	1.247367
H	-4.057626	4.485782	0.710087
H	-3.921638	3.417410	2.121549
H	-2.516735	4.372338	1.587650
C	-4.176150	1.867097	-0.154027
H	-3.878085	1.028224	-0.790577
H	-4.744833	1.484815	0.699571
H	-4.831941	2.512955	-0.746636
H	-1.898566	0.304886	-0.127425
C	-3.966993	-3.255586	1.125472
H	-3.432479	-4.195368	0.949538
H	-4.064653	-3.131197	2.209194
H	-4.977417	-3.356377	0.714746
C	-3.228865	-2.059867	0.530392
H	-2.235392	-1.983030	0.992342
H	-3.769088	-1.137777	0.781987
C	-3.041968	-2.094884	-0.991308
C	-2.171981	-3.266066	-1.440595
H	-2.682181	-4.221967	-1.286038
H	-1.943233	-3.182029	-2.509619
H	-1.227661	-3.285459	-0.884728
C	-4.372479	-2.075354	-1.738167
H	-4.918842	-3.012820	-1.596286
H	-5.004009	-1.250331	-1.389873
H	-4.202878	-1.951703	-2.814559
O	-2.333759	-0.865142	-1.283069
H	-2.250821	-0.750660	-2.240907

MsO⁻-tAmylOH

	Coordinates (Angstroms)		
	X	Y	Z
S	2.404436	0.131420	0.054846
C	3.491106	-1.279406	-0.052200
H	4.189792	-1.258716	0.787704
H	2.894551	-2.194362	-0.015522
H	4.043476	-1.235308	-0.993870
O	1.519324	0.037092	-1.139410
O	3.273880	1.328248	0.025293
O	1.663959	-0.025858	1.329493
C	-4.536713	-0.115033	0.472841
H	-4.454881	0.676154	1.226981
H	-5.544195	-0.054304	0.045589
H	-4.456465	-1.079639	0.986058
C	-3.489914	0.031920	-0.627664
H	-3.657712	0.972355	-1.169598
H	-3.615973	-0.776357	-1.360375
C	-2.022533	0.014375	-0.172999
C	-1.685766	1.224920	0.702333
H	-2.246143	1.218584	1.643921
H	-0.618747	1.222794	0.952474
H	-1.913402	2.155806	0.169715
C	-1.668246	-1.287243	0.551518
H	-2.201697	-1.381245	1.503896
H	-1.917450	-2.154327	-0.072210
H	-0.594392	-1.316028	0.770031
O	-1.279672	0.094935	-1.392818
H	-0.325275	0.089329	-1.175770

References

1. Frisch, M. J.; Trucks, G. W.; Schlegel, H. B.; Scuseria, G. E.; Robb, M. A.; Cheeseman, J. R.; Scalmani, G.; Barone, V.; Mennucci, B.; Petersson, G. A.; Nakatsuji, H.; Caricato, M.; Li, X.; Hratchian, H. P.; Izmaylov, A. F.; Bloino, J.; Zheng, G.; Sonnenberg, J. L.; Hada, M.; Ehara, M.; Toyota, K.; Fukuda, R.; Hasegawa, J.; Ishida, M.; Nakajima, T.; Honda, Y.; Kitao, O.; Nakai, H.; Vreven, T.; Montgomery Jr., J. A.; Peralta, J. E.; Ogliaro, F.; Bearpark, M.; Heyd, J. J.; Brothers, E.; Kudin, K. N.; Staroverov, V. N.; Keith, T.; Kobayashi, R.; Normand, J.; Raghavachari, K.; Rendell, A.; Burant, J. C.; Iyengar, S. S.; Tomasi, J.; Cossi, M.; Rega, N.; Millam, J. M.; Klene, M.; Knox, J. E.; Cross, J. B.; Bakken, V.; Adamo, C.; Jaramillo, J.; Gomperts, R.; Stratmann, R. E.; Yazyev, O.; Austin, A. J.; Cammi, R.; Pomelli, C.; Ochterski, J. W.; Martin, R. L.; Morokuma, K.; Zakrzewski, V. G.; Voth, G. A.; Salvador, P.; Dannenberg, J. J.; Dapprich, S.; Daniels, A. D.; Farkas, O.; Foresman, J. B.; Ortiz, J. V.; Cioslowski, J.; Fox, D. J. *Gaussian 09; Gaussian Inc.: Pittsburgh, PA, USA, 2010.*
2. Chai, J.-D.; Head-Gordon, M., Long-range corrected hybrid density functionals with damped atom-atom dispersion corrections. *Phys. Chem. Chem. Phys.* **2008,** *10*, 6615-6620.
3. Hay, P. J.; Wadt, W. R., Ab initio effective core potentials for molecular calculations. Potentials for k to au including the outermost core orbitals. *J. Chem. Phys.* **1985,** *82*, 299-310.
4. Ditchfield, R.; Hehre, W. J.; Pople, J. A., Self‐consistent molecular‐orbital methods. Ix. An extended gaussian‐type basis for molecular‐orbital studies of organic molecules. *J. Chem. Phys.* **1971,** *54*, 724-728.
5. Hehre, W. J.; Ditchfield, R.; Pople, J. A., Self—consistent molecular orbital methods. Xii. Further extensions of gaussian—type basis sets for use in molecular orbital studies of organic molecules. *J. Chem. Phys.* **1972,** *56*, 2257-2261.
6. Weigend, F.; Ahlrichs, R., Balanced basis sets of split valence, triple zeta valence and quadruple zeta valence quality for h to rn: Design and assessment of accuracy. *Phys. Chem. Chem. Phys.* **2005,** *7*, 3297-3305.
7. Neese, F., Software update: The orca program system, version 4.0. *WIREs Comput. Mol. Sci.* **2018,** *8*, e1327.
8. Neese, F.; Wennmohs, F.; Becker, U.; Riplinger, C., The orca quantum chemistry program package. *J. Chem. Phys.* **2020,** *152*, 224108.
9. Mardirossian, N.; Head-Gordon, M., Ωb97m-v: A combinatorially optimized, range-separated hybrid, meta-gga density functional with vv10 nonlocal correlation. *J. Chem. Phys.* **2016,** *144*, 214110.
10. Marenich, A. V.; Cramer, C. J.; Truhlar, D. G., Universal solvation model based on solute electron density and on a continuum model of the solvent defined by the bulk dielectric constant and atomic surface tensions. *J. Phys. Chem. B* **2009,** *113*, 6378-6396.
11. Hratchian, H. P.; Schlegel, H. B., Accurate reaction paths using a hessian based predictor-corrector integrator. *J. Chem. Phys.* **2004,** *120*, 9918-9924.
12. Grimme, S., Supramolecular binding thermodynamics by dispersion-corrected density functional theory. *Chem. Eur. J.* **2012,** *18*, 9955-9964.
13. Lu, T.; Chen, Q., Shermo: A general code for calculating molecular thermochemistry properties. *Comput. Theor. Chem.* **2021,** *1200*, 113249.
14. Bryantsev, V. S.; Diallo, M. S.; Goddard, W. A., Calculation of solvation free energies of charged solutes using mixed cluster/continuum models. *J. Phys. Chem. B* **2008,** *112*, 9709-9719.
15. Lu, T.; Chen, F., Multiwfn: A multifunctional wavefunction analyzer. *J. Comput. Chem.* **2012,** *33*, 580-592.

16. Lu, T.; Chen, Q., Independent gradient model based on hirshfeld partition: A new method for visual study of interactions in chemical systems. *J. Comput. Chem.* **2022**, *43*, 539-555.
17. Stephens, P. J.; Devlin, F. J.; Chabalowski, C. F.; Frisch, M. J., Ab initio calculation of vibrational absorption and circular dichroism spectra using density functional force fields. *J. Phys. Chem.* **1994**, *98*, 11623-11627.
18. Grimme, S.; Antony, J.; Ehrlich, S.; Krieg, H., A consistent and accurate ab initio parametrization of density functional dispersion correction (DFT-D) for the 94 elements H-Pu. *J. Chem. Phys.* **2010**, *132*, 154104.
19. Adamo, C.; Barone, V., Toward reliable density functional methods without adjustable parameters: The pbe0 model. *J. Chem. Phys.* **1999**, *110*, 6158-6170.
